# High‐Throughput Isolation of Nanomouse‐Derived VHH Domains: A Practical Guide from Immunization to Nanobody Expression

**DOI:** 10.1002/cpz1.70432

**Published:** 2026-07-21

**Authors:** Tessa J. Casselman, Asa W. Huffaker, Kristie C. Mitchell, Jamie E. Schnarrs, Sylvia Ni, Mary E. Skinner, Matthias C. Truttmann

**Affiliations:** ^1^ Department of Molecular & Integrative Physiology University of Michigan Ann Arbor Michigan; ^2^ Geriatrics Center University of Michigan Ann Arbor Michigan; ^3^ These authors contributed equally to this work

**Keywords:** nanobody, nanomouse, phage panning, single domain antibodies, VHH

## Abstract

Nanobodies are small but specific heavy chain–only antibody fragments. Their small size, relative stability, and ability to access difficult to reach deep‐tissue antigens makes them valuable research, diagnostic, and therapeutic tools. Nanobodies are derived from the variable heavy (VH) domain of heavy chain–only antibodies that are unique to camelids, including alpacas, llamas, and camels. The approaches employed to produce nanobodies, have been evolving and expanding since the initial discovery of heavy chain–only antibodies 30 years ago. Traditional nanobody development involves camelid immunization with a soluble, purified protein, followed by blood collection and processing, enrichment for potent nanobody sequences, and eventual expression and purification of candidate nanobodies for testing and validation. Alternative nanobody generation strategies aim to identify novel nanobodies utilizing synthetic or animal‐derived naïve nanobody libraries in combination with phage‐, yeast surface‐, or ribosome displays for nanobody selection. This article outlines a novel protocol series for nanobody production using a commercially available transgenic “nanomouse”, engineered to produce heavy chain–only antibodies containing camelid VH domains from alpacas, dromedaries, and Bactrian camels. These protocols will cover the following aspects: (1) Nanomouse breeding, genotyping, and colony establishment; (2) Nanomouse immunization and tissue collection; (3) RNA extraction from nanomouse immune cells isolated from blood and tissues; (4) Generation and amplification of VHH DNA from nanomouse cDNA; (5) Digestion of VHH DNA and ligation into a phagemid expression vector; (6) Preparation of a screenable *Escherichia coli* TG1‐based phagemid library; (7) Antigen‐driven VHH selection using phage display; (8) Single colony VHH ELISA screening; (9) Sequencing of ELISA hits and candidate VHH sequence identification; (10) Geneblock design of candidate VHH and Gibson Assembly into a nanobody expression vector; and (11) Nanobody over‐expression and purification. We provide a comprehensive toolkit to facilitate nanobody development and make it more accessible to the greater research community. © 2026 The Author(s). *Current Protocols* published by Wiley Periodicals LLC.

**Basic Protocol 1**: Nanomouse breeding, genotyping, and colony establishment

**Basic Protocol 2**: Nanomouse immunization and tissue collection

**Basic Protocol 3**: RNA extraction from nanomouse immune cells isolated from blood and tissues

**Basic Protocol 4**: Generation and amplification of VHH DNA from nanomouse cDNA

**Basic Protocol 5**: Digestion of VHH DNA and ligation into a phagemid expression vector

**Basic Protocol 6**: Preparation of a screenable *E. coli* TG1‐based phagemid library

**Basic Protocol 7**: Antigen‐driven VHH selection using phage display

**Basic Protocol 8**: Single colony VHH enzyme‐linked immunosorbent assay (ELISA) screening

**Basic Protocol 9**: Sequencing of ELISA hits and candidate VHH sequence identification

**Basic Protocol 10**: Geneblock design of candidate VHH and Gibson Assembly into a nanobody expression vector

**Basic Protocol 11**: Nanobody over‐expression and purification

## INTRODUCTION

Nanobodies are small antibody fragments derived from heavy chain–only antibodies. Unique to camelids, heavy chain–only antibodies bind to their antigens using the Variable Heavy chain domain of the Heavy chain–only antibody (VHH), rather than a heavy/light chain hetero‐oligomer as employed by conventional antibodies (Hamers‐Casterman et al., 1993). This VHH domain, referred to as single domain antibody or a nanobody, can be isolated and amplified synthetically through a series of molecular biology techniques and purified from *Escherichia coli* over‐expression cultures (Muyldermans, 2013). The structural stability, low production costs, practical applicability in various research approaches, and potential for use in diagnostic and therapeutic applications make nanobodies appealing tools for a multitude of disciplines (Muyldermans, 2013). Interest in nanobody generation and usage has progressively increased as exemplified by the growing number of nanobody‐centric academic publications and patents (https://pubmed.ncbi.nlm.nih.gov/?term=nanobody&sort=date&sort_order=asc). This protocol collection establishes an accessible resource to start working in the single‐domain antibody space.

The generation of nanobodies typically requires the immunization and blood collection from immunized camelids, creating a bottleneck in the applicability of this technology due to housing constrains, limited availability of animals, and restrictions in permissible antigens (Muyldermans, 2021; Zimmermann et al., 2018). To overcome these restrictions, alternative approaches, including in silico nanobody design and fully synthetic nanobody libraries have been developed (McMahon et al., 2018; Moutel et al., 2016; Zimmermann et al., 2018); however, these systems fail to capture the complexity of biologically relevant immune responses (Moutel et al., 2016). Transgenic mice that produce heavy chain–only antibodies, e.g., the nanomouse (Xu et al., 2021), offer an appealing new avenue for nanobody generation. These mice can be bred in great numbers, safely inoculated with more precarious antigens (Devasani et al., 2025), and are readily accessible to the larger research community.

These protocols will focus on approaches using the transgenic nanomouse established by Xu et al. (2021). The nanomouse, available from The Jackson Laboratory (strain no. 036538), carries a deletion of the CH1 exons from both the µ and γ1 constant regions of immunoglobulins and is lacking the mouse variable heavy domain (VH) locus. In addition, this mouse strain contains a VHH minigene, consisting of 30 nanobody ORFs from alpaca, dromedary, and Bactrian camels, along with regulatory elements. Homozygous nanomice thus produce heavy chain–only IgG and IgM antibodies carrying a camelid VHH domain.

Conceptually, nanobody development using nanomice is indifferent from the process used to generate llama‐derived nanobodies. Post immunization, RNA is extracted from isolated immune cells, VHH‐encoding domains are amplified, libraries are created and screened to identify candidate nanobodies for validation (Muyldermans, 2020). The protocols described here are derived from standard nanobody generation protocols (Schmidt et al., 2016; Sosa et al., 2014; Truttmann et al., 2015), repurposed and optimized for compatibility with the nanomouse model. The limitations of the proposed strategy include the need for an animal immunization step (e.g., as opposed to computationally designed and/or fully synthetic libraries) and the reliance on soluble proteinaceous antigens for immunization and nanobody identification. The key advantage of this approach is the pairing of a biologically relevant mammalian immune response with the commercial accessibility of the transgenic mouse model.

Basic Protocols 1 and 2 cover the mouse handling portion of the nanobody pipeline, detailing the breeding strategies, immunization procedures, and tissue collection from nanomice. Basic Protocol 3 outlines extraction of the RNA from nanomouse immune cells from blood, bone marrow, and spleen. Basic Protocols 4 and 5 cover the molecular biology techniques involved post‐RNA extraction, where mRNA is converted to cDNA and VHH sequences are amplified, digested, and ligated into a phagemid expression vector. Basic Protocol 6 details the preparation of a diverse *E. coli* TG1‐based phagemid library. Basic Protocol 7 describes the selection of antigen‐specific VHH via phage display through two rounds of phage panning against the antigen. Basic Protocol 8 describes a single colony enzyme‐linked immunosorbent assay (ELISA) for the identification of VHH that bind strongly to the antigen. Basic Protocol 9 describes how to sequence and analyze the hits identified in the single colony ELISA. Basic Protocol 10 explains how to convert candidate VHH sequences into geneblocks that are compatible with Gibson Assembly into a nanobody expression vector. Finally, Basic Protocol 11 details how to purify candidate nanobodies in mg quantities from *E. coli* over‐expression cultures for labeling, validation, functional assays, and practical application.

*NOTE*: All animal procedures were performed at the University of Michigan facilities according to National Institutes of Health guidelines and approved by the Animal Care and Use Program, the Unit for Laboratory Animal Medicine, and the Institutional Animal Care and Use Committee guidelines at the University of Michigan.

## Basic Protocol 1 NANOMOUSE BREEDING, GENOTYPING, AND COLONY ESTABLISHMENT

The innovation of a nanomouse model offers an alternative to camelid injections that allows for antigen targeting using an in vivo immune response without the limitations of large animal work. While breeding homozygous mice and/or littermates is often an effective strategy for maintaining mouse strains, we find that in the case of the nanomice, there is a reduction in breeding efficiency when either approach is employed. This protocol describes an empirically determined strategy to efficiently breed nanomice for nanobody generation. The protocol details how to purchase and breed nanomice, genotype them via PCR, and establish a nanomouse breeding colony.

### Materials

2 to 4 homozygous male nanomice, 8‐ to 12‐weeks old (The Jackson Laboratory, strain no. 036538)
4 to 8 wild‐type female C57BL/6J mice, 8‐ to 12‐weeks old (The Jackson Laboratory, strain 000664)
Tail digestion buffer (TDB) with proteinase K (see recipe)
Genotyping primers (IDT, 25 nmol, standard purification, diluted to 10 µM; see recipe):
  Wild type: AGGTGAAGGAAATGGTGCTG
  Common: ACGTTCCTGTGGCTAGAAGG
  Nanomouse: GTTTAGACTTGCGTGGTGCAG
Taq DNA polymerase (1000 U) (Qiagen, cat. no. 201205)
dNTP mix, 1 ml, 40 mM (10 mM of each) (Invitrogen, cat. no. 18427088)
UltraPure nuclease‐free water (Invitrogen, cat. no. 10977015)
Agarose gel, 1.8% (see recipe)
NEB 100 bp ladder working stock (see recipe)

1.5‐ml microcentrifuge tubes
10‐, 20‐, 200‐, and 1000‐µl filtered pipette tips and single channel pipettes
Heat block for microcentrifuge tubes
PCR strip tubes (VWR, cat. no. 76318‐800)
Thermocycler
Gel electrophoresis system
UV imager for DNA

#### Nanomouse colony breeding, genotyping, and colony establishment

**Figure 1:**
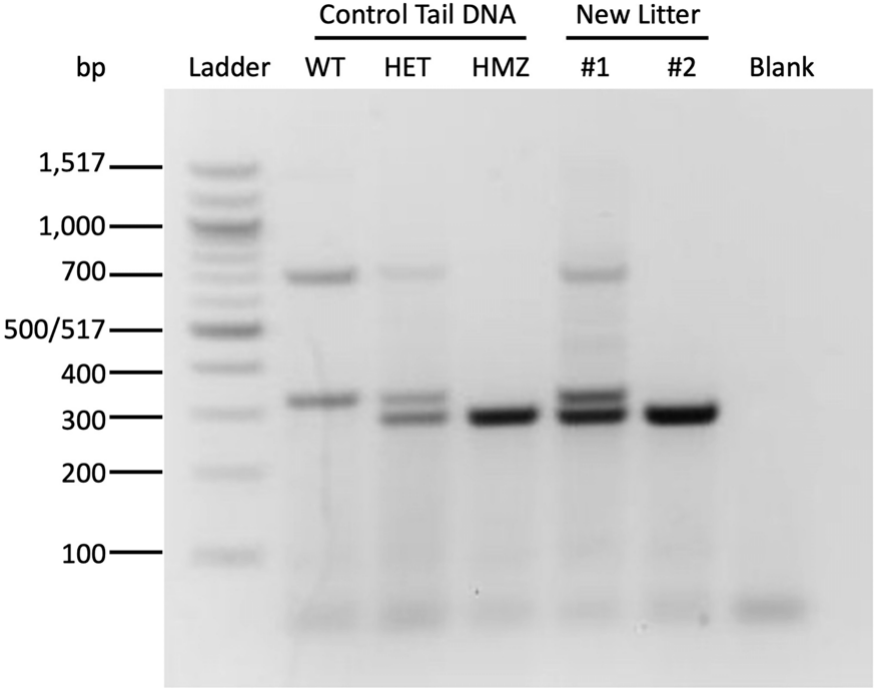
Agarose gel (1.8%) of PCR amplicons from nanomouse genotyping. Controls for wild‐type (329 bp), heterozygous (329/290 bp), and homozygous (290 bp) mice are all represented. Two lanes represent genotyping reactions for tail DNA PCR from new nanomouse litters (#1 and #2). WT (wild type); HET (heterozygous); HMZ (homozygous).

1 Obtain homozygous male nanomice and wild type female C57BL/6J mice, all at 8‐ to 12‐weeks old from The Jackson Laboratory. Keep tail snips to serve as PCR controls when there are litters of interest.
2 Breed each male mouse with 1 to 2 female mice. Breeding homozygous nanomice with wild‐type mice will result in litters that are 100% heterozygous.
  Take non‐littermate heterozygous offspring for the next generation of breeding. Collect tail snips from the heterozygous mice to serve as PCR controls when there are litters of interest. Genotype the offspring from the heterozygous crosses.
3 Prepare DNA from the tail snips. Conduct an overnight extraction using tail digestion buffer (TDB) with proteinase K.
  a. Use ∼2 mm tail snip per 1.5‐ml tube.
  b. Add 200 µl of tail digestion buffer with proteinase K.
  c. Incubate at 56°C overnight.
  d. Boil for 10 min to inactivate the proteinase K.
  e. Use DNA‐containing supernatant for PCR.
    Crude lysate using the DNA‐containing supernatant of the tail digests is sufficient and does not interfere with the PCR reactions in this protocol. The insoluble material (e.g., hair and bone) will settle to the bottom of the tube.
4 Make a PCR mix. Include a sample for wild type, heterozygous, and homozygous controls. For each reaction, a add to PCR strips or make the reaction in the strips:
  2 µl of tail DNA‐containing supernatant
  1 µl of each 10 µM primer (use all three primers listed in the Materials list)
  2.5 µl of 10× red buffer (provided with the Qiagen Taq)
  0.5 µl dNTP mix
  0.5 µl Qiagen Taq
  Bring to 25 µl final volume with UltraPure water.
5 Run the reaction in a thermocycler:
  94°C for 5 min
  35 cycles of 94°C for 30 s, 55°C for 30 s, and 72°C for 30 s
  72°C for 5 min
  Hold at 4°C.
6 Run the reaction products on a 1.8% agarose gel at 110 V for 60 min. Use 10 µl of NEB 100 bp ladder working stock to assess DNA band sizes.
7 Visualize on a UV imager. The expected PCR product sizes will be very close in size, ensure that a doublet can be seen in the heterozygous control. If the doublet is not observed, run the gel for a few minutes longer. PCR products will be 329 bp for wild type, 290 bp for nanomouse, and a doublet of each band for the heterozygous samples (Fig. 1).

8 Once multiple litters have been genotyped from different sets of parents, breed non‐littermate heterozygous by homozygous mice for strain maintenance and experimental groups.
  It is not recommended to breed homozygous mice or littermates together; these litters yield fewer pups than average. Breeding a homozygous mouse with a heterozygous mouse will provide typical mouse litter sizes yielding litters that are ¾ homozygous. This approach works best when the female breeder is heterozygous and the male breeder is homozygous. It is not recommended to continue breeding after 6 to 7 months of age. Refresh the breeding colony frequently.
9 As litters are obtained and genotyped from the crosses, group homozygous nanomice by sex in sets of three. Keep them as age matched as possible for experimental groups.

## Basic Protocol 2 NANOMOUSE IMMUNIZATION AND TISSUE COLLECTION

Largely aligned with the immunization strategies outlined in Xu et al. (2021), this protocol will go step by step through the process of immunization of nanomice and collection of immunogenic tissues from these mice. We propose some quality control steps to consider prior to starting an immunization campaign when working with purified protein and an optional assessment of serum immunogenicity via western blot after terminal blood collection. This protocol covers the quality assessment of purified antigens, subsequent intraperitoneal (IP) nanomouse immunization, periodical serum analysis from tail bleeds, and terminal cardiac puncture as well as spleen and bone marrow extraction. The deliverables of this protocol are frozen spleen, bone marrow, and whole blood from immunized mice ready for processing in Basic Protocol 3.

### Materials

1 ml of 1 mg/ml of purified protein in phosphate‐buffered saline (PBS) (this should be the protein that you wish to generate nanobodies against)
2× Laemmli buffer (see recipe)
12% SDS‐PAGE acrylamide gel (see recipe)
Precision Plus Protein Kaleidoscope prestained protein standard (Bio‐Rad, cat. no. 1610375)
1× running buffer (see recipe)
dH_2_O
Coomassie staining solution (see recipe)
Coomassie destaining solution (see recipe)
Homozygous nanomice 8‐ to 12‐weeks old (see Basic Protocol 1)
CFA: Freund's Adjuvant, Complete (MP Biomedicals cat. no. INC642851)
IFA: Freund's Adjuvant, Incomplete (MP Biomedicals cat. no. INC642861)
1× PBS (see recipe)
100% ethanol (Fisher, cat. no. 04‐355‐222)
2‐ml tubes containing RNAlater (obtained from the Mouse RiboPure kit; see Basic Protocol 3)
Liquid nitrogen
Isoflurane (MWI, cat. no. 501017)
70% ethanol (see recipe)
Methanol, histological (Fisher, cat. no. A433P)
1× transfer buffer (see recipe)
Ponceau S solution (see recipe)
5% milk in TBST (see recipe)
1× TBST (see recipe)
VHH‐HRP secondary antibody cocktail for western blot (see recipe)
Immobilon western chemiluminescent HRP substrate (MilliporeSigma, cat. no. WBKLS0500)

10‐, 20‐, 200‐, and 1000‐µl filtered pipette tips and single channel pipettes
1.5‐ml microcentrifuge tubes
Heat block
Criterion Cell (Bio‐Rad, cat. no. 1656001)
Orbital rocker (Genesee Scientific, cat. no. 31‐438)
Beadsmith case (Amazon, cat. no. B08633RNLW)
Flatbed color scanner (any compatible model will work)
Air‐Tite sterile syringes with needles, Luer Slip, 25‐G (Fisher, cat. no. 14‐817‐125)
Red Tailveiner restrainer for mice, standard id (BrainTree Scientific, cat. no. TV‐RED 150‐STD)
Kimtech Science Kimwipes delicate task wipers (Fisher, cat. no. 06‐666)
Razor blades (Garvey, cat. no. 091461)
Refrigerated microcentrifuge, 4°C
0.5‐ml flat‐cap PCR tubes (Fisher, cat. no. 14‐230‐200)
18‐G BD Precision Glide needle (BD, cat. no. 305195)
Sterile mouse dissection tools and pins
Liquid nitrogen dewar
Drop Jar or other container for mouse anesthesia
Spray bottle for 70% ethanol
Styrofoam lid or rectangular pad (we use the backside of 50‐ml Falcon tube Styrofoam racks)
Terumo syringe and needle, 1‐ml, removable needle, 27‐G, Tuberculin (Med Vet International, cat. no. SS‐01T2713)
Ice container with ice
Immobilon‐PSQ PVDF membranes (MilliporeSigma, cat. no. ISEQ00010)
Lab gloves
Criterion blotter system (Bio‐Rad, cat. no. 1704070)
15‐ml conical tube
Chemiluminescent imager

#### Mouse immunization and test bleeding

**Figure 2:**
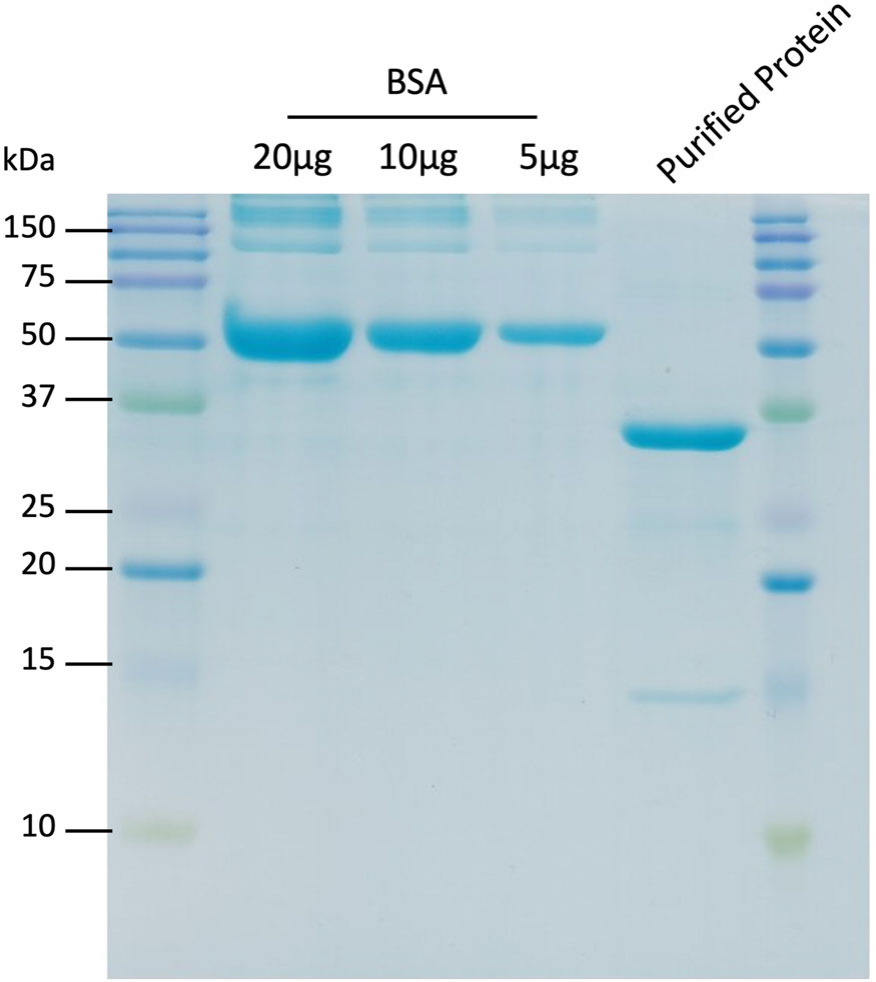
Protein quality control SDS‐PAGE‐Coomassie stained gel. A dose curve of known concentrations of BSA protein and subsequently followed by a purified protein, all loaded onto a 12% SDS‐PAGE acrylamide gel. Typical results for Coomassie staining, followed by destaining are represented here.

1 Confirm that the purified protein that has been selected to immunize the mice with has not degraded:
  a. Take 10 µg of the 1 mg/ml purified protein in PBS and add 10 µl of 2× Laemmli buffer to it in a 1.5‐ml tube.
  b. Boil the sample for 5 min in a heat block.
  c. Load the entire sample into one lane of a 12% SDS‐PAGE acrylamide gel and load 10 µl of Precision Plus Protein Kaleidoscope prestained protein standard into the ladder wells.
    If there are spare wells in the gel, prepare several BSA concentrations in Laemmli Buffer as was done for the purified protein to serve as standards for comparison from 1 to 20 µg (e.g., 1 µg, 2.5 µg, 5 µg, 10 µg, 20 µg). Fill any empty wells with 20 µl of 1× Laemmli buffer.
  d. Run the 12% SDS‐PAGE acrylamide gel in SDS‐PAGE 1× running buffer for 65 min at 150 V in a Criterion Cell.
  e. Remove the 12% SDS‐PAGE acrylamide gel from its cassette and gently rinse with deionized water.
  f. Submerge the gel in Coomassie staining solution and gently rock overnight at room temperature.
    Any container that is approved for gel staining will work. Beadsmith boxes are preferred as they seem to provide the best seal, minimizing evaporation reagents.
  g. Remove the Coomassie staining solution and rock the gel in Coomassie destaining solution, replacing the destaining solution every hour for ∼4 hr at room temperature or until clear resolution of protein bands can be seen.
  h. Scan the gel on a flatbed scanner and evaluate the quality of the protein, making sure it is the expected size, concentration, and not degraded (Fig. 2).

2 Confirm that the appropriate animal protocol approvals are in place for immunizing the mice with the protein of interest.
3 Identify a cohort of three homozygous nanomice that are 8‐ to 12‐weeks old for the primary injection group. Ensure the mice have unique identifiers (e.g., ear‐tags, notches, tattoos, etc.)
  Both sexes of nanomice work for immunization campaigns. Females are preferred for immunization due to ease of handling, robust immune responses, and cost‐savings associated with cohabitation.
4 Prepare a 400 µl solution for injection: 200 µl of CFA mixed with 200 µl of the 1 mg/ml purified protein stock in PBS. This solution is very heterogeneous; mix well before loading the syringes.
5 Load three 25‐G syringes with 100 µl of the solution for injection.
  An extra 100 µl of solution is made in step 10 as some solution loss will occur. This is a viscous solution and should be loaded slowly into the syringe.
6 After loading, point the syringe vertically with the tip of the needle facing upwards. Pull back on the syringe for 300 to 500 µl pulling in air and forcing the stock solution down in the syringe. Push the plunger back up the syringe until the solution has the air purged from it. Verify there is still 100 µl of solution in the syringe.
7 Inject 100 µl of the solution into each of the three mice via intraperitoneal injection, where the needle is inserted bevel up in line with the natural extension of the hip and angled at ∼45° to the abdomen, penetrating the peritoneal space. The 45° angle will be sharp enough to ensure that the injection is not subcutaneous. This equates to 50 µg of purified protein injected per mouse and is the Day 0 injection.
8 On Days 14, 28, 42, 56, and 59, repeat steps 5 to 7, but for these injections make the stock solution for injection with 200 µl IFA, 100 µl PBS, and 100 µl of the 1 mg/ml purified protein stock. This will equate to 25 µg purified protein per mouse for each booster injection.
9 On Days 16, 30, and 44, take tail bleeds from each mouse:
  a. Confirm that tail bleeding is an approved protocol for your laboratory.
  b. Use a Red Tailveiner restrainer or alternative restraint device to position the mouse in the device with the tail exposed.
  c. Wipe the tail with a Kimwipe soaked in 100% ethanol and dry the tail with a Kimwipe.
  d. Use a sterile razor blade to nick end of the tail.
  e. Hold the tail over an open microcentrifuge tube to collect 15 to 50 µl of blood dripping from the tail.
  f. Take a dry Kimwipe and elevate the mouse tail, pinching it firmly with the Kimwipe to stop the bleeding. Hold in place for 1 to 2 min or until the nick has stopped bleeding and return the mouse to its cage.
  g. Close the microcentrifuge tube without agitating it. Allow the blood to clot in the tube for 1 hr at room temperature.
  h. Centrifuge the clotted blood for 15 min at 2000 × *g*, 4°C.
  i. Collect the clear upper layer of serum with a pipette, taking care not to touch the bottom layer. Transfer the serum to a fresh tube and store at –20°C.

#### Mouse tissue collection

10 On Day 62 prepare for terminal bleeding and tissue dissection of the nanomice.
  a. Label 5 microcentrifuge tubes for each mouse. One tube for each of the following: whole blood, serum, spleen, bone marrow, and tail.
  b. Puncture a 0.5‐ml individual flat‐cap PCR tube with an 18‐G needle, one punctured tube for each mouse.
  c. Label one 2‐ml tube per mouse of RNAlater, which is provided in the Mouse RiboPure RNA kit used for RNA processing from blood listed in Basic Protocol 3 of this article.
  d. Sterilize the dissection tools and pins.
  e. Get a dewar of liquid nitrogen.
  f. Prepare the isoflurane drop jar.
11 Place one mouse in the isoflurane drop jar. Observe the mouse until breathing has stopped and conduct a toe pinch to ensure the mouse is completely sedated or overdosed with anesthesia. Once a complete depression in breathing is observed and the mouse is not responsive to pain (e.g., toe pinch) move quickly to step 12. The longer the interval after death, the less successful the cardiac puncture procedure.
12 Spray the mouse with 70% ethanol and pin the mouse in supine position on a dissecting board by gently extending the limbs and affixing them to the board using dissecting pins placed through the distal extremities ensuring that the animal remains immobilized with the body adequately splayed for optimal tissue exposure.
13 Make a midline incision using sterile fine scissors from the lower abdomen to the mandible and reflect the skin laterally to expose the abdominal musculature.
14 Incise the abdominal wall musculature to expose the diaphragm and ribs. Carefully incise the diaphragm and incise the rib cage laterally to expose the heart.
15 Using a sterile tuberculin syringe, carefully insert the needle into the left ventricle of the heart and gently aspirate to collect 500 to 700 µl of blood. It is critical that this is performed swiftly to prevent clotting. If the aspiration stops drawing blood, gently turn the needle to refresh the flow.
16 Eject ∼100 µl of blood from the syringe to the microcentrifuge tube labeled whole blood and eject the remaining 400 to 600 µl of blood into the 2‐ml tube of RNAlater. Immediately put the tube of blood/RNAlater in a –20°C freezer and allow the microcentrifuge tube containing 100 µl of whole blood to clot at room temperature for 1 hr.
17 Remove the spleen and take a section of tail from the mouse. Freeze both tissues in liquid nitrogen in their corresponding microcentrifuge tubes.
18 Extract the bone marrow from both femurs of the mouse. Any method of extraction will be sufficient. The centrifugation method performed in Zhu et al. (2019), which will require a punctured 0.5‐ml flat‐cap PCR tube to be nested in a 1.5‐ to 1.7‐ml microcentrifuge tube is preferred.
19 Freeze the bone marrow in liquid nitrogen.
20 Process the clotted blood as was done for the tail bleeds in steps 9h‐i.
21 Frozen blood and tissue can then be processed for RNA as outlined in Basic Protocol 3 or stored at –80°C.
22 Frozen serum can be stored at –20°C until it is time to proceed with analysis.

#### Sera analysis by western blot (optional)

**Figure 3:**
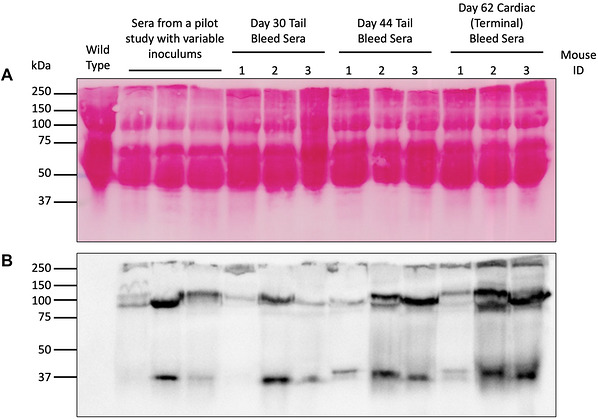
Western blotting of nanomouse serum over time post‐inoculation. (**A**) Ponceau S staining of the post‐transfer PVDF membrane that is used to assess total protein loading. (**B**) Developed western blot using VHH‐HRP secondary antibody cocktail to visualize anti‐camelid proteins in the mouse serum via chemiluminescent signal. Non‐reduced heavy chain–only antibodies run at 90 to 100 kDa. Reduced heavy chains lacking CH1 run 35 to 45 kDa.

23 Thaw frozen serum from the tail bleeds and terminal bleeds on ice.
24 Prepare microcentrifuge tubes, one tube for each sample, by pipetting 5 µl of 2× Laemmli buffer into each tube.
25 Pipette 5 µl of serum into the 5 µl of 2× Laemmli Buffer, non‐reducing, and boil the sample for 5 min.
  If the serum is pinkish red, it can be centrifuged again to attempt to pellet the red blood cells or hemolysis components before taking out 5 µl. Samples with too much heme contamination will coagulate when boiled and can run poorly on the SDS‐PAGE, causing issues with transfer onto a PVDF membrane.
26 Load 6 µl of boiled sample per well on a 12% SDS‐PAGE acrylamide gel.
  a. Load the samples in order of the date that they were collected.
  b. Load 10 µl of Precision Plus Protein Kaleidoscope prestained protein standard into the ladder wells of the gel.
    A Criterion midi‐size 12‐well gel is ideal for a complete set of sera from an antigen group of three mice.
27 Run the 12% SDS‐PAGE acrylamide gel in SDS‐PAGE 1× running buffer for 65 min at 150 V in a Criterion Cell.
28 Remove the gel from the cassette and trim off the top portion of the gel containing the wells and the thick bottom portion of the gel ∼2 mm from the bottom.
  This trimming helps provide a smooth transfer of gel products on to the PVDF membrane. Do not trim the gel too much on the top or the bottom and retain the area that is within the ladder coverage.
29 Cut a rectangle of PVDF that is roughly the size of the acrylamide gel. Wear gloves to not introduce proteins from your hands on to the membrane. Activate the PVDF membrane by submerging it in pure methanol.
30 Rinse the PVDF membrane in 1× transfer buffer.
31 Soak the foam pads and a few pieces of filter paper in 1× transfer buffer; both the pads and the paper are provided with the Criterion Blotter system.
32 Assemble the Criterion blotter system cassette for transfer of the gel onto a membrane per the manufacturer's instructions (see Internet Resources)
33 Transfer the 12% gel onto a PVDF membrane in 1× transfer buffer at 35 V overnight at 4°C.
34 Remove PVDF membrane from the transfer and gently rinse it in deionized water.
35 Submerge the membrane in Ponceau S solution and gently rock for 15 min.
36 Rinse off the Ponceau S solution with deionized water until bands are resolved.
37 Scan the membrane on a flatbed scanner and save the image as a reference for serum loading (Fig. 3A).

38 Block the membrane by submerging it in 5% milk in TBST and gently rock it for 30 min.
39 Rinse the membrane a few times with 1× TBST.
40 Prepare a VHH‐HRP secondary antibody cocktail for western blot.
41 Remove the membrane from blocking and incubate it in the VHH‐HRP secondary antibody cocktail for 1 hr with gentle rocking, making sure that the rocking allows for the secondary antibody mixture to wash over the whole membrane surface.
42 Wash the membrane with 1× TBST three times for 10 min each.
43 Mix 5 ml of each Immobilon western chemiluminescent HRP substrate in a 15‐ml conical tube (1:1 ratio).
44 Remove the TBST from the membrane and incubate the membrane in the HRP substrate mixture for 1 min.
  Letting the PVDF membrane incubate in the HRP substrate for too long can have detrimental effects on the experiment. Depending on the intensity of the signal, the HRP substrate can burn into the membrane, making the signal uninterpretable. Confirm the imaging system is available for use and the bulbs in the chemiluminescent imager are cooled and ready for exposing the blot. Do not add the HRP substrate to the membrane until the machine is ready to use for imaging.
45 Image the membrane on a chemiluminescent imager.
  The resulting images provide a visual of how reactive the mice were to the immunization campaign (Fig. 3B). A wild‐type mouse serum sample will yield no signal, validating that what is detected are heavy chain–only antibodies. A range of band sizes from 75 to 110 kDa, consistent with predicted molecular weights of antibodies lacking light chains should be observed. An increase in signal intensity may be observed with later timepoints in the immunization campaign, but occasionally loss of signal occurs. Increased signal intensity does not have a direct correlation with increased chances of identifying on‐target VHH sequences but does inform the best candidates for testing crude serum for antigen specific binding (e.g., whole cell immunofluorescence, flow cytometry).

## Basic Protocol 3 RNA EXTRACTION FROM NANOMOUSE IMMUNE CELLS ISOLATED FROM BLOOD AND TISSUES

Basic Protocol 3 describes how to extract RNA from isolated whole blood, spleen, and bone marrow. The protocol details sample‐specific considerations and required materials for cell lysis, RNA isolation, and DNase treatment. Completion of this protocol will deliver purified RNA from relevant tissues of injected nanomice, ready for reverse transcription to produce cDNA in Basic Protocol 4.

*CAUTION*: Wear long sleeves, non‐porous shoes, cryo‐gloves, and safety glasses when working with liquid nitrogen to avoid contact of this extremely cold, volatile substance with skin or eyes.

*CAUTION*: Beta‐mercaptoethanol is a highly toxic compound that should only be handled in a fume hood while wearing gloves. It is a mutagen, can cause organ damage if inhaled, and is combustible and corrosive. It is also a potent environmental toxin and should be disposed of per OSEH regulations.

### Materials

RNase‐Zap (MilliporeSigma, cat. no. R2020)
Blood, spleen, and bone marrow samples (from Basic Protocol 2)
UltraPure nuclease‐free water (Invitrogen, cat. no. 10977015)
Invitrogen Mouse RiboPure‐Blood RNA isolation kit (Fisher, cat. no. AM1951)
100% ethanol (Fisher, cat. no. 04‐355‐222)
DNase I Recombinant, RNase‐free, with 10× buffer (Roche, cat. no. 04716728001)
Zymo RNA Clean & Concentrator kit (Zymo, cat. no. R1018)
Dry ice
Liquid nitrogen
14.3 M Beta‐mercaptoethanol (MilliporeSigma, cat. no. 444203)
Qiagen RNeasy Plus Mini kit (Qiagen, cat. no. 74134)
Wet ice
Qiagen QiaShredder kit (Qiagen, cat. no. 79654)
70% ethanol (see recipe)

Falcon brand 15‐ml conical tubes (Corning, cat. no. 352097)
Vortex
Microcentrifuge
10‐, 20‐, 200‐, and 1000‐µl filtered pipette tips and single channel pipettes
Non‐microbial centrifuge with a rotor suited for 15‐ml conical tubes
Incubator or heat block for 1.5‐ and 2‐ml microcentrifuge tubes
Microcentrifuge tubes
Nanodrop or similar equipment to measure nucleic acid concentration
5‐ml tubes (Eppendorf, cat. no. 0030119401)
Ceramic mortars and pestles
Metal spatula with rounded end
Dry ice and liquid nitrogen compatible buckets
Liquid nitrogen dewar
Sonicator for tissue lysis with standard probe (e.g., Fisherbrand Model 120 Sonic Dismembrator, cat. no. FB120110)

*NOTE*: Both sections in this protocol are heavily reliant on the multiple kits listed in the materials section; have all required kits and reagents on hand before starting.

#### RNA extraction from blood

1 Spray all work surfaces, including pipettes, centrifuges, and gloves, with RNase‐Zap or another similar RNase treatment.
2 Aliquot enough nuclease‐free water to have 100 µl per blood sample from Basic Protocol 2 and incubate it at 80°C; this will be used in a later step.
3 Prepare two 15‐ml conical tubes for each blood sample, labeling as “set 1” and “set 2”.
4 Thaw blood samples that were frozen in RNAlater at room temperature, vortex to mix, then spin in a microcentrifuge for 3 min at maximum speed, room temperature.
5 Pipette off and discard the supernatant.
  Steps 6 to 26 involve use of the Invitrogen Mouse RiboPure Blood RNA isolation kit. All reagents referenced in these steps come from the kit except for nuclease‐free water. Before beginning, ensure that there is enough of each reagent and that the appropriate volume of 100% ethanol has been added to Wash Solution 1 and Wash Solution 2/3 concentrates. All centrifugations in these steps should be done at room temperature.
6 Add 700 to 1000 µl of Lysis Solution to the blood pellet, enough to almost fill the 2‐ml tube, leaving a ∼1 cm gap at the top.
7 Vortex vigorously to resuspend the blood pellet and lyse the cells. The sample should be completely dislodged and broken apart by pipetting, if necessary, in addition to vortexing before proceeding to the next step.
8 Add 200 µl of 3 M sodium acetate to each 15‐ml conical tube in set 1.
9 Transfer each lysed blood sample into its respective 15‐ml tube and vortex again for 5 s.
10 Rinse the 2‐ml tube with 1 ml of Lysis Solution and add this to the lysate in the 15‐ml tube.
11 Add 1.5 to 2 ml more Lysis Solution to the 15‐ml tube; the total volume should be ∼3.8 ml. Vortex for 5 s.
12 Pipette 1.5 ml of solution from the bottom layer of the bottle of Acid‐Phenol:Chloroform, add it to the same 15‐ml tube of cell lysate, then shake the tube vigorously for 30 s to mix.
13 Incubate the mixture for 5 min at room temperature.
14 In a non‐microbial centrifuge, spin the 15‐ml tubes for 10 min at 2000 × *g*.
15 Transfer up to 4.2 ml of the upper aqueous layer to its corresponding “set 2” 15‐ml conical tube. Discard the bottom organic phase.
16 Add 2.4 ml of nuclease‐free water to the aqueous phase isolated in the previous step, then vortex for 5 s.
  This may cause the sample to become opaque.
17 Add 7.7 ml of 100% ethanol, for a total volume of 13 to 15 ml, then shake the tube vigorously until the solution becomes clear. If the sample remains cloudy or shows signs of secondary phase separation, continue adding nuclease‐free water in 300 µl increments and shaking until homogeneity and clarity are reached.
18 Add 700 µl of the solution from the previous step to a filter cartridge assembly and centrifuge 5 to 10 s at ≥8000 × *g*. Discard the flowthrough.
19 Repeat the previous step until all ∼15 ml of solution has been passed through the filter cartridge.
20 Wash the filter cartridge with 750 µl of Wash Solution 1, spinning 5 to 10 s at ≥8000 × *g*. Discard the flowthrough.
21 Repeat the step 20 wash once.
22 Wash the filter cartridge with 750 µl of Wash Solution 2/3, spinning 5 to 10 s at ≥8000 × *g*. Discard the flowthrough.
23 Repeat step 22 once.
24 Spin the filter assembly for 1 min at maximum speed, room temperature, to remove any residual wash buffer, then transfer the filter cartridge to a new collection tube.
25 Add 100 µl of the previously prepared 80°C nuclease‐free water to the filter and incubate at room temperature for 1 min.
26 Centrifuge for 1 min at maximum speed, room temperature, to elute the RNA.
27 Using the Roche DNase I recombinant enzyme kit, set up the following 150‐µl reaction for each RNA sample to remove any DNA from the sample:
  100 µl RNA
  15 µl of 10× reaction buffer
  1 µl Roche DNase I
  33 µl nuclease‐free water.
28 Incubate the reaction for 30 min at room temperature, then proceed directly to the Zymo RNA Clean & Concentrator kit to remove the DNase and concentrate the RNA sample.
  It is not necessary to quantify RNA until after using the Clean & Concentrator kit; however, if an alternative kit is being used, confirm the binding capacity of the columns in the kit is appropriate for the RNA yield.
29 Reapply RNase‐Zap to all work surfaces.
30 Working with the RNA Clean & Concentrator kit, add 300 µl RNA Binding Buffer to each 150 µl RNA sample and mix by pipetting.
31 Add 450 µl of 100% ethanol and mix again.
32 Prepare one collection tube and column for each sample, as well as one microcentrifuge tube.
  The following centrifugation steps should all be carried out at room temperature for 30 s at 15,000 × g unless otherwise specified. Before using the RNA Wash Buffer, ensure that the appropriate volume of 100% ethanol has been added to it; see bottle.
33 Transfer 700 µl of each sample to its respective column and spin, then discard the flowthrough. Repeat with the remaining 200 µl.
34 Add 400 µl of RNA Prep Buffer to the column, spin, and discard the flowthrough.
35 Add 700 µl of RNA Wash Buffer to the column, spin, and discard the flowthrough.
36 Add 400 µl of RNA Wash Buffer to the column and spin for 1 min.
37 Transfer the column from the collection tube to the prepared microcentrifuge tube.
38 Pipette 50 µl of nuclease‐free water onto the column, incubate at room temperature for 1 min, then centrifuge to elute the concentrated, DNA‐free RNA.
39 Quantify samples using a Nanodrop or similar equipment for measuring RNA concentration, then store at –80°C until ready to proceed to cDNA synthesis.
  RNA yields will vary by mouse and by volume of blood initially collected, with an expected yield of 0.3 to 1 µg/µl of RNA per mouse when eluting in 50 µl.

#### RNA extraction from spleen or bone marrow

40 Retrieve tissue samples from –80°C and keep on dry ice to prevent thawing and tissue degradation. Add one 5‐ml tube per sample, or per group of tissues if combining, to the dry ice; ground samples will be stored in these tubes.
  Pooling tissues by antigen is optional and will depend on whether there are multiple mice per antigen; pooling three of the same tissue type per antigen greatly increases time and reagent usage efficiency without sacrificing yield.
41 Place one mortar and pestle per tissue, or one set per group of tissues if working with multiple mice for the same antigen, into a clean ice bucket along with a metal spatula. Wearing appropriate PPE for handling liquid nitrogen (see Caution above), cool the implements by pouring liquid nitrogen into the mortar and filling ∼1‐in. of the ice bucket.
  Once bubbling and evaporation have slowed, the instruments will have been sufficiently cooled to begin tissue grinding. If working with multiple tissues and mortars, keep all mortars and pestles except the one in use in a second ice bucket containing liquid nitrogen to stay cold until needed. Attempting to wash and reuse the same mortar and pestle is not recommended because they need to be thawed, washed, and refrozen, taking at least 30 min.
42 Fill the mortar about halfway with liquid nitrogen and add one spleen or bone marrow into the liquid nitrogen. Press the pestle down onto the tissue gently but firmly and twist slightly to grind, keeping the pestle vertical to avoid accidentally launching brittle tissue out of the mortar; this is primarily an issue with bone marrow.
43 Once the tissue(s) has been ground to a fine powder, use the metal spatula to transfer it into a 5‐ml tube, or larger if needed for pooled tissues.
  Add enough liquid nitrogen that it will finish evaporating just as grinding is complete. This will prevent tissue thawing while also limiting transfer of liquid nitrogen into the sample tube. If pooling multiple tissues, tissues of the same type can be ground all at once. However, this increases the risk of some sample exiting the mortar or all the liquid nitrogen evaporating before grinding is complete.
44 If grinding like tissues from the same antigen one at a time and then pooling them, repeat this process for the remaining tissues using the same mortar and pestle. Otherwise, repeat for other tissues using a clean, pre‐cooled mortar and pestle set. Once all tissues have been ground and are in 5 ml (or larger) tubes on dry ice, proceed to the next step.
45 In a fume hood, combine beta‐mercaptoethanol with RLT Plus buffer from the Qiagen RNeasy Plus Mini kit at a ratio of 1:100 to make a lysis buffer. 2400 µl of lysis buffer will be needed per spleen or 600 µl per bone marrow, scaling up as needed for pooled tissues.
46 Still working in the fume hood, resuspend each tube of ground tissue in the appropriate volume of lysis buffer. For example, for a pool of three spleens, 7.2 ml of lysis buffer is needed. Incubate on wet ice for 20 min.
47 Precool the sonicator probe by submerging it in a beaker of wet ice.
48 Sonicate each tube of lysate as follows: constant pulse for 30 s at 50% amplitude using a 120 W sonicator, rest on ice for 30 s, then repeat for a total of two rounds of sonication. In between rounds, submerge the sonicator probe in the beaker of ice to prevent it from warming.
49 Transfer the sonicated tissue lysate to QIAshredder columns 600 µl at a time. Use four columns per spleen and one column per bone marrow, distributing the solution evenly between columns. Centrifuge 2 min at 16,000 × *g*, room temperature. Keep the flowthrough; this is the filtered tissue lysate. Keep the tube on ice.
  Optional pause point: the lysate can now be stored at –80°C if needed. Otherwise, proceed to the next step.
50 Spray down all work surfaces, including a microcentrifuge, with RNAse‐Zap.
  Steps 51 to 59 will follow the Qiagen RNeasy Plus Mini kit. Before starting, add the appropriate volume of 100% ethanol to the RPE Buffer; see reagent bottle. Carry out all centrifugation steps at room temperature.
51 Begin by transferring the lysate from the QIAshredder columns to gDNA eliminator spin columns, using one gDNA column per QIAshredder column used. Centrifuge 30 s at 10,000 × *g*. Keep the flowthrough; this is nucleic acid from which gDNA has now been removed.
52 Add 550 µl of freshly made 70% ethanol to the flowthrough in each collection tube and mix by pipetting.
53 Transfer the lysate‐ethanol mixture onto RNeasy columns 700 µl at a time, combining the flowthrough onto two RNeasy columns per spleen or one RNeasy column per bone marrow. Centrifuge 15 s at 10,000 × *g*, then discard the flowthrough.
54 Repeat step 53 until all the lysate has been run through the RNeasy columns.
55 Discard the flowthrough and wash each column by adding 700 µl of RW1 Buffer and spinning 15 s at 10,000 × *g*.
56 Discard the flowthrough and wash the column again, this time with 500 µl of RPE Buffer, spinning 15 s at 10,000 × *g*.
57 Discard the flowthrough and wash again with 500 µl RPE Buffer but spin 2 min at 10,000 × *g*.
58 Remove the flowthrough and spin 1 min at 16,000 × *g*, to remove any remaining buffer.
59 Transfer each RNeasy column onto a new 1.5‐ml microcentrifuge tube, add 50 µl of RNase‐free water by carefully pipetting it onto the column near the center of the filter disc, and then incubate for 5 min at room temperature. Spin 1 min at 10,000 × *g*, to elute the RNA. Place on ice immediately after spin is complete to prevent RNA degradation. A set of three spleens requires the use of six separate columns for elution; pool these samples together for a total volume of 300 µl.
60 Run each sample through DNase treatment and the Zymo RNA Clean & Concentrate kit as described in steps 27 to 38 of this protocol, using 100 µg of RNA per tissue pool. Store excess RNA at –80°C.
  RNA concentration ranges vary by mouse with a typical yield of 1 to 2.5 µg/µl of RNA for three pooled spleens and 0.5 to 1 µg/µl of RNA for three pooled bone marrows when eluting in 50 µl.
61 Quantify the cleaned and concentrated RNA, then store at –80°C until ready to move on to cDNA synthesis.
  The RNA concentration should remain very close to 100 µg total because the input was 100 µg. However, up to a 20% loss is possible and still provides enough material for subsequent protocols.

## Basic Protocol 4 GENERATION AND AMPLIFICATION OF VHH DNA FROM NANOMOUSE cDNA

Basic Protocol 4 describes how to amplify VHH regions of heavy chain–only antibodies from cDNA. First, mRNA from the total RNA pool extracted in Basic Protocol 3 is reverse transcribed into cDNA. The resulting cDNA is then amplified using 30 VHH‐specific forward primers and four VHH‐specific reverse primers for a total of 120 primer combinations that capture the diversity of VHH sequences generated by the immunized nanomice. PCR products are assessed on an agarose gel and cleaned using the PureLink PCR purification kit. At the end of this protocol, the amplicons from the purified PCR reactions will be ready for digestion and ligation in Basic Protocol 5.

*CAUTION*: When operating a UV gel imager, take care to protect eyes and skin from direct interaction with UV radiation. Wear gloves and long sleeves with no gap at the wrist that would expose skin, and wear eyeglasses or a face shield to prevent eye damage.

### Materials

RNase‐Zap (MilliporeSigma, cat. no. R2020)
RNA samples (from Basic Protocol 3)
QuantaBio qScript XLT cDNA SuperMix (VWR, cat. no. 10142‐788)
UltraPure nuclease‐free water (Invitrogen, cat. no. 10977015)
Library‐specific VHH forward and reverse primers (IDT, 25 nmol, standard purification, diluted to 10 µM, see recipe) that are compatible with cloning into the phagemid vector being used in Basic Protocol 5
NEB 5× Phusion buffer (NEB, cat. no. M0530L)
NEB Phusion DNA polymerase (NEB, cat. no. M0530)
dNTP Mix, 1 ml, 40 mM (10 mM of each) (Invitrogen, cat. no. 18427088)
Agarose gel, 1.8% (see recipe)
NEB 100 bp ladder working stock (see recipe)
NEB 6× DNA loading dye (NEB, cat. no. B7024)
Invitrogen PureLink PCR purification kit (Fisher, cat. no. K310002)
100% isopropanol
100% ethanol (Fisher, cat. no. 04‐355‐222)

Microcentrifuge
Ice container with ice
10‐, 20‐, 200‐, and 1000‐µl filtered pipette tips and single channel pipettes
PCR strip tubes (VWR, cat. no. 76318‐800)
Thermocycler
96‐well, non‐skirted PCR plates (Thermo Scientific, cat. no. AB‐0600)
Adhesive PCR plate seals (Thermo Scientific, cat. no. AB‐0558)
Gel electrophoresis system
UV imager for DNA gels
Microcentrifuge tubes
Nanodrop or similar equipment that can measure nucleic acid concentration

#### cDNA extraction

1 Spray gloves and all work surfaces with RNase‐Zap.
2 Thaw RNA on ice, microcentrifuge 30 s at 10,000 × *g*, 4°C, and determine the volume needed for 3 µg of RNA per cDNA reaction.
  Set up six cDNA reactions of 3 µg RNA each per RNA sample and then pool them together for use in VHH PCRs. If there is <18 µg of RNA, scale down to 1 to 2 µg of RNA per reaction.
3 Spin down a tube of qScript XLT cDNA SuperMix and immediately put on ice; it will already be liquid and needs to be kept cold, or it will degrade.
4 Prepare the following reaction (Table 1) on ice, in PCR strip tubes:
  4 µl qScript cDNA XLT SuperMix
  3 µg RNA
  Nuclease‐free water to bring the total reaction volume to 20 µl.
    Scale up as needed depending on how many samples are being amplified and desired cDNA amount to be generated per sample. Add the water to each tube first, followed by qScript, and finally RNA. If preparing several reactions from the same RNA sample, make a master mix of the RNA and water to minimize pipetting very small volumes.

**Table 1:** cDNA Synthesis Reaction Used in Basic Protocol 4, Step 4

Component	Volume (µl)
qScript cDNA XLT SuperMix	4
Nanomouse RNA	3 µg
Nuclease‐free water	16 – RNA
Total reaction volume	20

5 Place PCR strip tube(s) in a thermocycler and run the following program (Table 2):
  25°C for 5 min
  42°C for 1 hr
  85°C for 5 min
  Hold at 4°C.

**Table 2:** cDNA Thermocycler Program Used in Basic Protocol 4, Step 5

Temp (°C)	Time (min)
25	5
42	60
85	5
4	∞

6 Pool all cDNA reactions made from the same RNA sample and store at –20°C if proceeding to the next step in 1 to 2 days, or at –80°C for long term storage.

#### PCR to amplify VHH region

This section uses 30 forward primers and 4 reverse primers described by Xu et al. (2021) from their published nanomouse Phage Library Nest PCR. These primers were modified by replacing the plasmid overhangs for compatible ligation into our specific phagemid vector (Table 3). The restrictions sites used in these methods differ from the ones in the paper due to overhang modification. Design primers to be specific to your procured phagemid vector. Run four sets of 30 PCR reactions, with each set containing one of the reverse primers in the master mix, and a different forward primer in each reaction.

**Table 3:** Nanomouse Specific Primer Regions to be Modified for VHH PCR Used in Basic Protocol 4, Step 7

Phage library nest PCR ID	Nest PCR primers*^b^*	Required translation in phagemid plasmid	Primer ID in our VHH PCR
phage_SfiI_F_Exon_1	CAAGTTCAGCTTGTAGAGTCAGGCG	QVQLVESG	Forward #1
phage_SfiI_F_Exon_3	CAAGTTCAGCTTGTCGAGAGTGGTG	QVQLVESG	Forward #2
phage_SfiI_F_Exon_4	CAGGTCCAATTGGTGGAATCTGGG	QVQLVESG	Forward #3
phage_SfiI_F_Exon_7	CAGGTTCAACTCGTTGAGTCAGGC	QVQLVESG	Forward #4
phage_SfiI_F_Exon_8	CAAGTTCAGTTGGTGGAAAGTGGG	QVQLVESG	Forward #5
phage_SfiI_F_Exon_17	CAGGTCCAACTGGTAGAAAGTGGG	QVQLVESG	Forward #6
phage_SfiI_F_Exon_23	CAGGTGCAATTGGTAGAGTCTGGAG	QVQLVESG	Forward #7
phage_SfiI_F_Exon_28	CAAGTTCAACTCGTAGAATCTGGCGG	QVQLVESG	Forward #8
phage_SfiI_F_Exon_20	CAAGTGCAATTGGTTGAATCTGGTGG	QVQLVESG	Forward #9
phage_SfiI_F_Exon_16	CAGCTTCAACTGGTAGAGAGTGGTGG	QLQLVESG	Forward #10
phage_SfiI_F_Exon_9	CAAGTAAAACTTGAAGAATCAGGTGGTGG	QVKLEESGG	Forward #11
phage_SfiI_F_Exon_15	CAAGTAAAACTCGAAGAGAGCGGG	QVKLEESG	Forward #12
phage_SfiI_F_Exon_22	CAGGTGCAGGTTGTGGAAAGC	QVQVVES	Forward #13
phage_SfiI_F_Exon_2	CAGGTTCAGCTGGTGGAGTCC	QVQLVES	Forward #14
phage_SfiI_F_Exon_5	CAAGTGCAACTTGTCGAGAGCG	QVQLVES	Forward #15
phage_SfiI_F_Exon_10	CAAGTCCAGCTCGTCGAGAGTG	QVQLVES	Forward #16
phage_SfiI_F_Exon_11	CAGGTCCAATTGGTGGAAAGCG	QVQLVES	Forward #17
phage_SfiI_F_Exon_18	CAGGTCCAACTGGTAGAGTCAGG	QVQLVES	Forward #18
phage_SfiI_F_Exon_24	CAAGTGCAGTTGGTTGAGAGCG	QVQLVES	Forward #19
phage_SfiI_F_Exon_25	CAGGTTCAACTCGTCGAATCCGG	QVQLVES	Forward #20
phage_SfiI_F_Exon_26	CAAGTTCAGCTTGTGGAGAGCG	QVQLVES	Forward #21
phage_SfiI_F_Exon_30	CAGGTCCAGCTTGTCGAATCCG	QVQLVES	Forward #22
phage_SfiI_F_Exon_6	GACGTACAACTTGTGGAATCAGGTGG	DVQLVESG	Forward #23
phage_SfiI_F_Exon_12	GAGGTCCAGGTCGTCGAATCAGG	EVQVVES	Forward #24
phage_SfiI_F_Exon_14	GAAGTGCAGGTTGTCGAGTCCG	EVQVVES	Forward #25
phage_SfiI_F_Exon_19	GAGGTCCAACTCGTCGAATCTGG	EVQLVES	Forward #26
phage_SfiI_F_Exon_13	GAGGTACAACTTGTGGAAAGCGGTG	EVQLVESG	Forward #27
phage_SfiI_F_Exon_27	GAAGTTCAGCTTGTTGAATCAGGCG	EVQLVESG	Forward #28
phage_SfiI_F_Exon_29	GAAGTTCAACTTGTCGAAAGCGGAGG	EVQLVESG	Forward #29
phage_SfiI_F_Exon_21	GAAGTCCAACTTGTAGAGAGTGGG	EVQLVESG	Forward #30
phage_JH1_SfiI_R	GAGGAGACGGTGACCGTGGT	TTVTVSS	Reverse #1
phage_JH2_SfiI_R	GAGGAGACTGTGAGAGTGGT	TTLTVSS	Reverse #2
phage_JH3_SfiI_R	GCAGAGACAGTGACCAGAGT	TLVTVS	Reverse #3
phage_JH4_SfiI_R	GAGGAGACGGTGACTGAGGT	TSVTVSS	Reverse #4

^*a*^ From Xu et al. (2021), Supplemental Table 4.

^*b*^ Conserved region of the nest PCR primers. Overhangs are added to be compatible with our phagemid vector (overhangs not shown).

**Figure 4:**
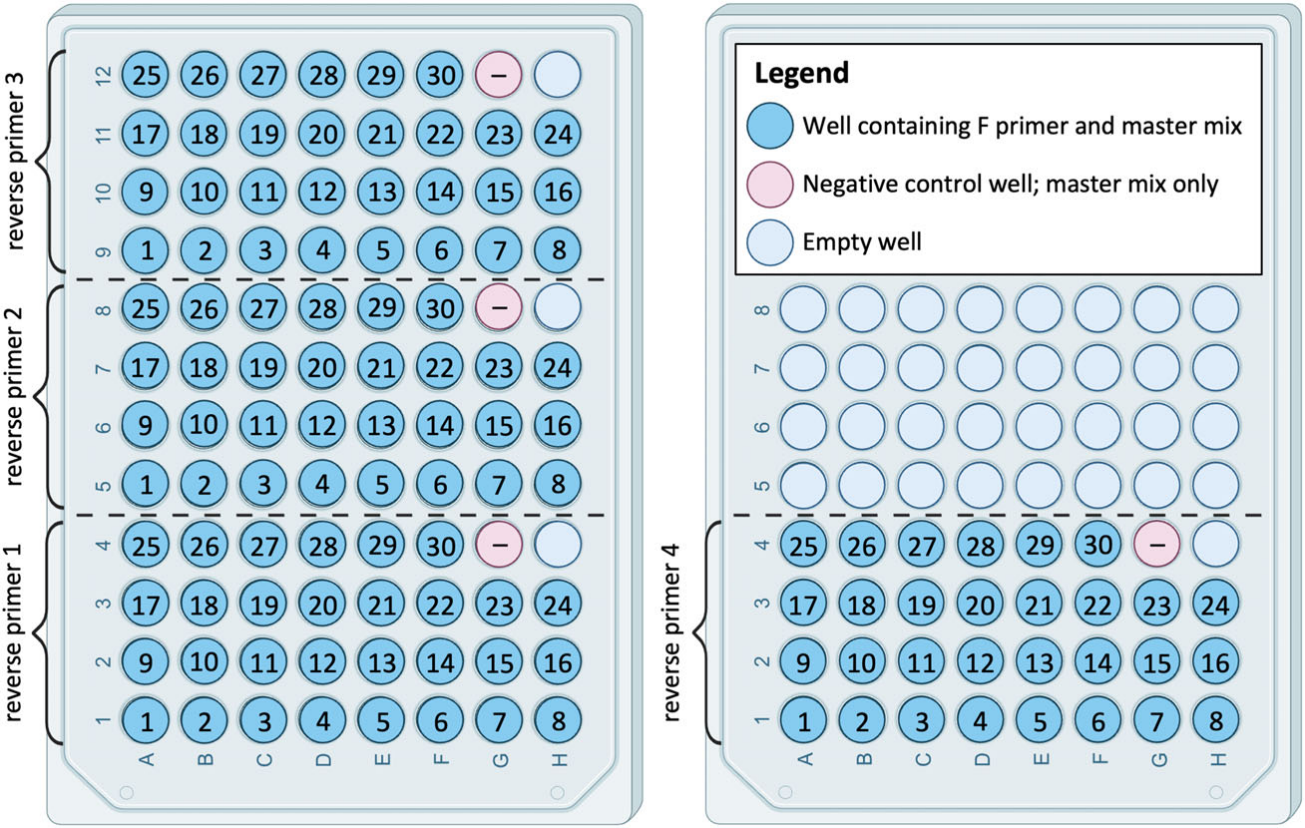
Suggested plate layout and strategy for VHH PCRs. Setup for four sets of 30 PCR reactions (blue) and one negative control (pink) in two 96‐well plates. Each set contains all 30 forward primers and one reverse primer. Step 1: Add 2.5 µl of forward primer to each well; different F primer per well. Step 2: Add 47.5 µl of master mix containing reverse primer and cDNA to each well; same in all wells within one set of 30 reactions plus negative control.

7 In a 96‐well plate, set up 30 PCR reactions, 50 µl per reaction. Begin by adding 2.5 µl of 10 mM forward primer to each well, with a different F primer in each of the 30 wells. See Figure 4 for suggested plate setup.

8 Make the following master mix using the NEB Phusion Polymerase kit and one of the reverse primers (Table 4). Repeat for the other three reverse primers for a total of four master mixes; these do not all need to be made at once. Divide the total volume of the cDNA sample to be amplified by four and add that ¼ volume to each of the four reverse primer master mixes.
  330 µl of 5× Phusion buffer
  82.5 µl of 10 µM reverse primer
  33 µl of 10 mM dNTP mix
  33 µl Phusion DNA polymerase
  ¼ of the total volume of the cDNA sample to be amplified
  Nuclease‐free water equivalent to 1089 µl minus the volume of ¼ of the cDNA.

**Table 4:** VHH PCR Reaction Setup Used in Basic Protocol 4, Step 8

Component	Volume (µl)
UltraPure water	1089 – cDNA volume
5× Phusion buffer	330
10 µM reverse primer	82.5
10 mM dNTP mix	33
Phusion DNA polymerase	33
cDNA	¼ sample total

9 Add 47.5 µl of master mix to each of the 30 wells containing F primer, plus an extra well to serve as a negative control. If there is leftover master mix, distribute it amongst non‐control wells rather than throwing it out, as it contains cDNA.
10 Seal the plate with adhesive PCR seals and run the PCR on a thermocycler using the following program (Table 5):
  98°C for 5 min
  35 cycles of 98°C for 10 s, 60°C for 30 s, and 72°C for 50 s
  72°C for 10 min
  Hold at 4°C.

**Table 5:** VHH PCR Thermocycler Program Used in Basic Protocol 4, Step 10

Step	Temperature (°C)	Time (min:s)	Cycles
Initial denaturation	98	05:00	1
Denaturation	98	00:10	35 cycles of denaturation, annealing, and extension
Annealing	60	00:30	
Extension	72	00:50	
Final extension	72	10:00	1
Hold	4	∞	1

**Figure 5:**
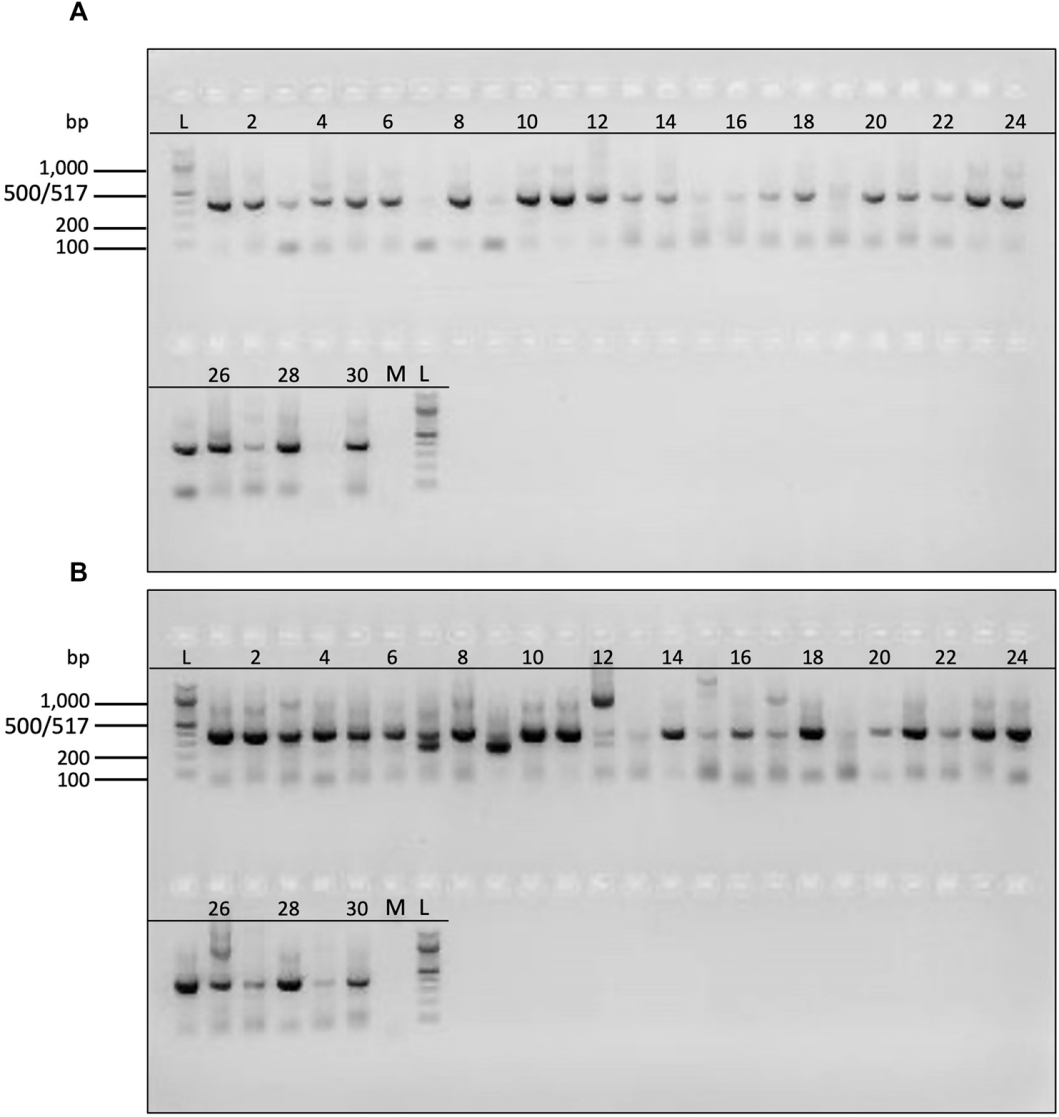
Example of VHH‐specific verification gels. (**A**) Agarose gel (1.8%) of PCR amplicons of VHH regions using 30 unique camelid forward primers and one conserved reverse primer based on camelid VHH sequences, visualized using gel electrophoresis. (**B**) VHH amplification products for the same 30 forward primers on the same DNA sample used in (**A**), but with a different reverse primer. The band(s) around 340‐440 bp are the VHH amplicons; any other bands are off‐target amplifications or primer dimers. The differences between (**A**) and (**B**) highlight the diversity in VHH generation captured by different primer combinations even within the same DNA sample. Even‐numbered primer wells are labeled, along with the ladders (L) and the master mix (M).

11 Visualize the PCR products on a 1.8% agarose gel as follows:
  a. Combine 5 µl of each sample with 1 µl of 6× DNA loading dye.
  b. Load each sample into individual wells.
  c. Include a well in each row containing 6 µl of NEB 100 bp ladder working stock to assess DNA band sizes.
  d. Run at 110 V for 30 min.
  e. Visualize on a UV imager.
    Depending on primer modifications, the VHH amplicon bands should appear around 440 bp. There will be variation in PCR products between reactions, with some 440 bp bands much more pronounced and many reactions containing off‐target bands (Fig. 5). A few lanes might be empty except for some primer dimers at the bottom; it is likely the VHH sequence(s) corresponding to a combination of primers was not produced by that nanomouse. It is not expected that the mouse will make antibodies corresponding to all 120 possible combinations.

12 Pool the remaining 45 µl from all 30 reactions made with the same reverse primer into one 5‐ml tube. This can be stored at –20°C until ready to proceed to the next step.
  The negative control does not contain any PCR product and should be discarded. Final volume should be 1 to 1.3 ml per pool of 30 reactions.

#### PCR purification

To increase efficiency, it is recommended wait to conduct the PCR purification until all four PCR sets for one tissue type from one antigen group have been completed. The following steps are carried out at room temperature.

13 Use the Invitrogen PureLink PCR purification kit. Follow the protocol provided, start by adding 4 ml of B2 buffer to each 5‐ml tube containing a PCR pool. Ensure the appropriate volume of 100% isopropanol has been added to the B2 buffer before use and that 100% ethanol has been added to the wash buffer.
  Per Invitrogen, the binding capacity of the columns in this kit is 40 µg. One column is sufficient per PCR pool.
14 Once the entire PCR pool has been run through the column and the wash step is complete, elute the purified DNA in 35 µl of nuclease‐free water in a new 1.5‐ml tube. Incubate at room temperature for 5 min before the elution spin to give the DNA more time to elute off the column.
  Use nuclease‐free water rather than the elution buffer provided for optimal activity in downstream assays.
15 Quantify each DNA sample using a Nanodrop or similar equipment that can measure nucleic acid concentration.
16 These samples can be stored at –20°C until ready for the next step.
  Set‐up for the PCR digest in the next protocol is brief, but the digest requires overnight incubation. It is recommended to proceed directly to Basic Protocol 5. The eluted DNA from this protocol can be kept on ice with the digestion enzymes during set‐up for Basic Protocol 5.

## Basic Protocol 5 DIGESTION OF VHH DNA AND LIGATION INTO A PHAGEMID EXPRESSION VECTOR

Basic Protocol 5 describes how to digest the VHH PCR products obtained in Basic Protocol 4, use gel purification to isolate the VHH regions, removing any off‐target amplification from the digested PCR and ligate the gel‐purified product into a phagemid plasmid. By the end of this protocol, all the VHH amplicons generated in Basic Protocol 4 will be combined and ligated into a phagemid vector.

*CAUTION*: When operating a UV gel imager, take care to protect eyes and skin from direct interaction with UV radiation. Wear gloves and long sleeves with no gap at the wrist that would expose skin, and wear lab safety glasses or a face shield to prevent eye damage.

*NOTE*: This protocol contains two overnight steps, first for the digest and then for the ligation. It is recommended to set up the digest reaction immediately following the PCR purification completed in Basic Protocol 4 to avoid delaying the proceeding steps with an 18‐hr wait step.

### Materials

Purified VHH PCR products (from Basic Protocol 4)
NEB 10× CutSmart buffer or best performance buffer indicated for your restriction enzyme
Restriction digest enzymes compatible with cloning into chosen phagemid vector
*The vector we use is compatible with NotI (NEB, cat. no. R3189) and AscI (NEB, cat. no. R0558)*.
UltraPure nuclease‐free water (Invitrogen, cat. no. 10977015)
Phagemid vector plasmid (e.g., Antibody Design Laboratories, cat. no. PD0105)
Agarose gel, 1% (see recipe)
NEB 6× DNA loading dye (NEB, cat. no. B7024)
NEB 100 bp ladder working stock (see recipe)
GeneRuler 1 kb DNA ladder (Thermo Scientific, cat. no. SM0311)
Zymoclean Gel DNA Recovery kit (Zymo Research cat. no. D4008)
NEB T4 DNA ligase (NEB, cat. no. M0202)
NEB 10× T4 DNA ligase buffer
Invitrogen PureLink PCR purification kit (Fisher, cat. no. K310002)

10‐, 20‐, 200‐, and 1000‐µl filtered pipette tips and single channel pipettes
Microcentrifuge tubes
Incubator or heat block for 1.5‐ and 2‐ml microcentrifuge tubes
50°C incubator or heat block that can accommodate 50‐ml conical tubes
Gel electrophoresis system
UV imager for DNA gels
Gel releasers (Bio‐Rad, cat. no. 1653320)
Falcon brand 50‐ml conical tubes (Corning, cat. no. 352098)
Microcentrifuge
Nanodrop or similar equipment that can measure nucleic acid concentration
Ice bucket and ice
5‐ml tubes (Eppendorf, cat. no. 0030119401)

#### Restriction digest of VHH PCR products

**Figure 6:**
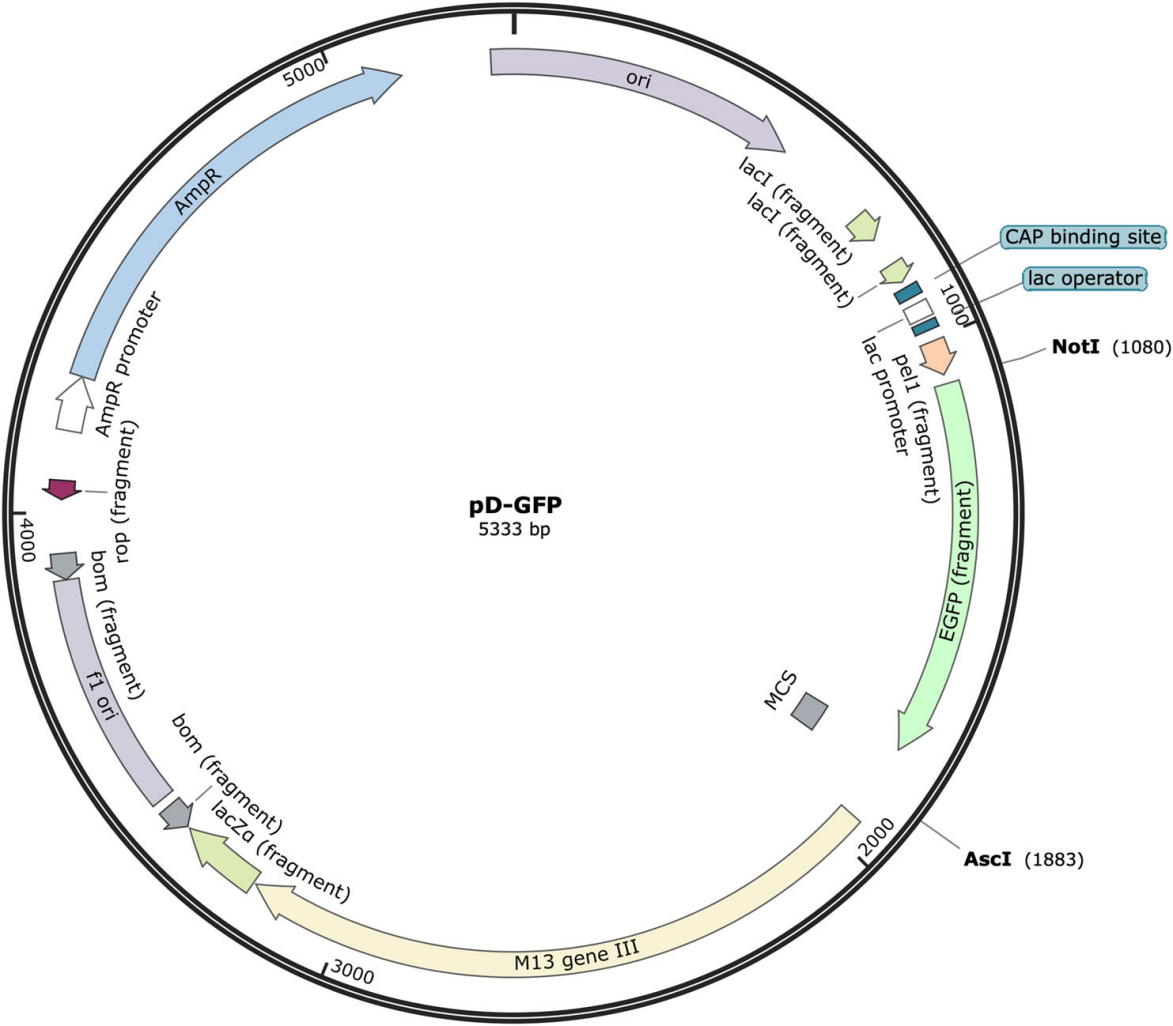
Schematic overview of the phagemid vector used for VHH amplicon ligation and downstream phage display. Plasmid map of pD‐GFP generated using SnapGene software (Dotmatics).

The VHH PCR products will be digested for ligation into a phagemid vector. The phagemid vector used here is not commercially available. A schematic of this vector (Fig. 6) highlights the restriction sites AscI and NotI described in this protocol; it retains the signature properties of standard phagemid vectors available for purchase like the example listed in the materials section. Use different restriction enzymes as necessary to be compatible with the chosen phagemid vector and VHH primer sequences used in Basic Protocol 4.

1 Set up the following 375 µl digest reaction, on ice, of the VHH amplicons (Table 6):
  ≤30 µg of PCR product
  37.5 µl NEB CutSmart buffer
  7.5 µl each of AscI and NotI
  Nuclease‐free water equivalent to 322.5 µl minus the volume of the PCR product.
    Keep reverse primer sets separate; there will be four digest reactions per antigen. If the sample exceeds 30 µg of DNA, scale up reaction size as needed.

**Table 6:** VHH Restriction Digest Reaction Used in Basic Protocol 5, Step 1

Component	Volume (µl)
PCR product	≤30 µg
UltraPure water	322.5 – PCR product volume
10× NEB CutSmart buffer	37.5
AscI	7.5
NotI	7.5

2 Incubate overnight in a heat block at 37°C. The following morning, heat to 80°C for 20 min to inactivate the restriction enzymes, then proceed to gel purification or store samples at –20°C.

#### Restriction digest of phagemid vector plasmid

3 The reaction for plasmid digestion involves the same enzymes and buffer as PCR amplicon digestion but can accommodate up to 75 µg of plasmid DNA. Set up as follows:
  ≤75 µg of PCR product
  37.5 µl NEB CutSmart buffer
  7.5 µl each of AscI and NotI
  Nuclease‐free water equivalent to 322.5 µl minus the volume of the plasmid.
4 Incubate overnight in a heat block at 37°C. The following morning, heat to 80°C for 20 min to inactivate the restriction enzymes, then proceed to gel purification or store samples at –20°C.

#### Gel purification

**Figure 7:**
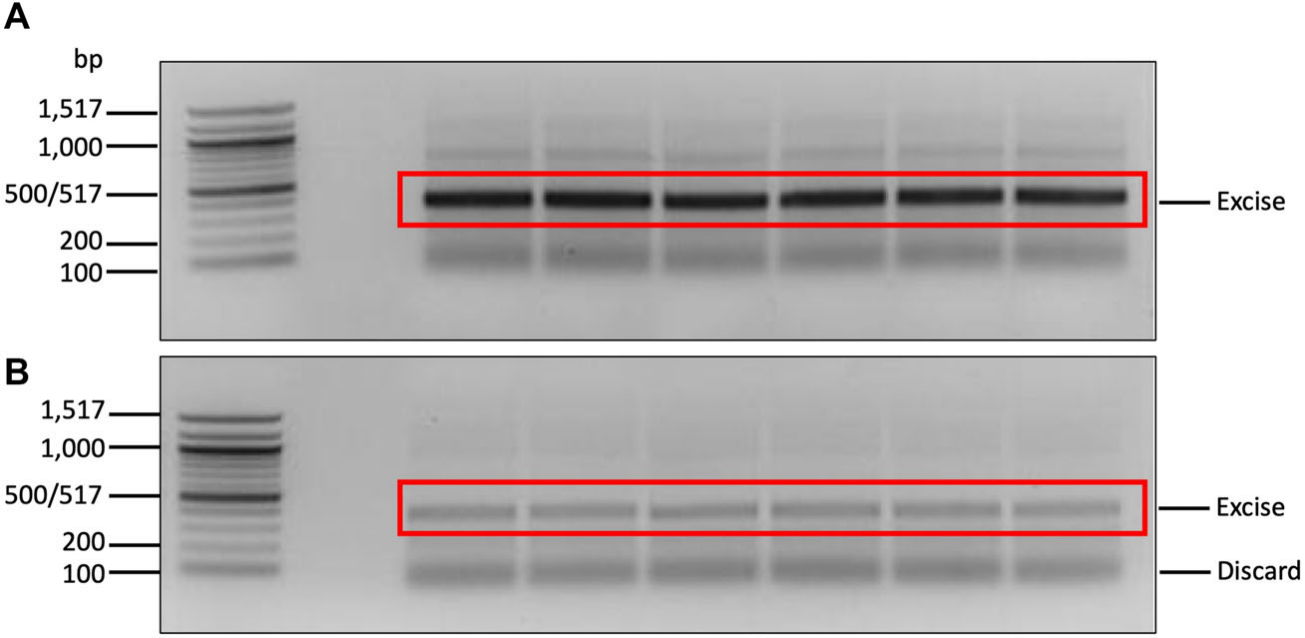
Example of digested VHH PCR. (**A**) Example of a gel digest in which the VHH amplicons, ∼440 bp in size, are the main product and show up clearly on the UV imager. Excised bands boxed in red. (**B**) Example of the same DNA sample run with the same forward primers and a different reverse primer; here, there is an equally strong band around 100 bp, but this must be discarded.

5 This section can be applied to digested PCR products or digested plasmid. Thaw the samples to be purified at room temperature. Meanwhile, set a heat block that can accommodate 50‐ml conical tubes or an incubator to 50°C.
6 Make a 100 ml of 1% agarose gel using two combs with as few large wells as possible, e.g., combs with ≤8 wells. Alternatively, tape can be used to cover parts of the comb to create larger wells. To purify all four digested PCR samples or four plasmid digests at once, make two of these gels; one 375 µl sample should fit into one gel row, spread across as many wells as needed with one well reserved for the ladder.
7 Add NEB 6× DNA loading dye or an equivalent loading dye to each tube of digest, load samples into the gel, and run for 60 min at 110 V. The PCR products can be run for longer to increase band resolution if needed, whereas the linearized plasmid should only need 30 min to reach a clear distinction between the large band to be retained and the small band to be discarded. Use the NEB 100 bp ladder working stock for PCR digests and the GeneRuler 1 kb DNA Ladder for the plasmid digest.
8 View the gel on a UV imager to confirm that the band of interest is present and excise the band of interest using a plastic cutting tool such as a Bio‐Rad gel releaser to avoid scratching the UV imager. For VHH amplicons, this will be ∼440 bp; for the plasmid it will be ≥4000 bp depending on the plasmid. Keep as little empty gel as possible to maximize DNA yield and quality and to minimize reagents and time needed to purify the sample. Chop up the gel band of interest finely to reduce dissolving time during the next step. Transfer the gel fragments into a 50‐ml conical tube. Repeat this for all four PCR digest sets, putting each in a separate tube.
  For VHH digests, the band at 440 bp will be fuzzy due to the heterogeneity of VHH amplicons and may be very faint (Fig. 7). Faint bands may appear above or below 440 bp, or a stronger band around 100 bp; these are off‐target amplicons and primer dimers, respectively, and should be discarded. For plasmid digests, the plasmid band will be clear and distinct from the much smaller insert fragment, unless there is no insert to be removed from the selected phagemid plasmid, then, there will only be one clear linear product.

9 Weigh each tube of gel fragments, then add 3× volume of ADB buffer from the Zymoclean Gel DNA Recovery kit to each tube. For example, if the gel in one tube weighs 2 g, add 6 ml of ADB to that tube. Incubate the gel–ADB mixture at 50°C until gel fragments are fully dissolved.
  Depending on the volume of gel being dissolved, this will take 30 to 60 min. Briefly agitate the tube(s) every 10 min to help dissolution occur.
10 Run each sample through Zymoclean Gel DNA Recovery purification columns 800 µl at a time, spinning for 1 min at 15,000 × *g*, room temperature.
  The binding capacity of these columns is 5 µg; use an appropriate number of columns for the approximate DNA concentration. Four columns are enough for one 375 µl digest reaction.
11 Wash the columns twice with 200 µl of wash buffer, spinning each time 1 min at 15,000 × *g*, room temperature.
12 Elute in 20 to 30 µl nuclease‐free water. After adding water to the column, incubate at room temperature for 5 min to maximize DNA recovery, then spin to elute for 1 min at 15,000 × *g*, room temperature.
13 Quantify the eluted DNA using a Nanodrop or equivalent device.
  Expect to see much lower DNA yields than those measured at the end of the VHH PCR. 260/280 and 260/230 ratios may also be poor at this stage; this will be resolved during the next cleanup step. Poor ratios after gel purification do not interfere with ligation of DNA into the phagemid vector plasmid.

#### Ligation of VHH amplicon into phagemid vector plasmid

14 On ice, set up a reaction to ligate the VHH amplicons into the linearized phagemid vector plasmid using NEB T4 DNA ligase and buffer. Scale to the nearest µg of VHH DNA (Table 7). For every 1 µg of gel purified PCR digest:
  1.75 µg of linearized phagemid vector plasmid
  25 µl NEB 10× T4 DNA ligase buffer
  12.5 µl NEB T4 DNA ligase
  Nuclease‐free water equivalent to 212.5 µl minus the total volume of the gel purified PCR digest and the linearized plasmid.
    Use discretion with rounding up or down. For example, for a yield of 2.4 µg with good 260/280 and 260/230 ratios, scale up to the reagent volumes for 3 µg, but for 2.4 µg with poor ratios, scale down.

**Table 7:** VHH Ligation Reaction Used in Basic Protocol 5, Step 14

Component	Volume (µl)
Gel purified VHH PCR digest	X (∼1 µg)
Linearized phagemid plasmid	Y (∼1.75 µg)
UltraPure water	212.5 – X – Y
10× T4 DNA ligase buffer	25
T4 DNA ligase	12.5

15 Incubate at 16°C overnight. This incubation temperature can be achieved using a heat block in a cold room. Proceed directly to the cleanup step or store ligated products at –20°C.
16 Use the Invitrogen PureLink PCR Purification kit to clean up the ligation reactions. Pool the four ligation reactions that correspond to the same tissue and antigen and divide the pool onto two purification columns. The DNA binding capacity for each column is 40 µg; use more columns if total pooled DNA would exceed this limit. The samples will be combined after elution.
17 Transfer pooled samples to a 5‐ml tube, or larger if necessary, and add B2 buffer equivalent to 4× sample volume.
18 Following the kit protocol, run total volume through purification column(s) 650 to 700 µl at a time with spins of 1 min each at 13,000 × *g*, room temperature.
19 Wash with 650 µl wash buffer and spin 1 min at 13,000 × *g*, room temperature, remove supernatant, and spin again for 3 min at maximum speed, room temperature.
20 Elute in 35 µl of nuclease‐free water, or less if a higher DNA concentration is desired, by centrifuging 1 min at maximum speed, room temperature. Let water sit on column for 5 min before spinning to maximize DNA elution.
  Avoid eluting in the E5 buffer provided with the kit because it may interfere with electroporation in Basic Protocol 6. However, E5 may be appropriate for samples that are difficult to elute or long‐term storage.
21 Pool the elutions corresponding to one set of ligation reactions, then quantify. Keep samples at 4°C for short term storage or move to –20°C for longer term storage. These samples are now ready for transformation into a phage‐competent *E. coli* library. For one antigen, using three mice, there should now be five sets of purified ligation reactions: three for blood, one for spleen, and one for bone marrow.

## Basic Protocol 6 PREPARATION OF A SCREENABLE *E. coli* TG1‐BASED PHAGEMID LIBRARY

In Basic Protocol 5, VHH amplicons were isolated via gel purification and ligated into phagemid vectors. In Basic Protocol 6, these phagemids will be transformed into TG1 *E. coli* to make a phagemid library that can then be screened for antigen specific VHH binding by panning via phage display. This is done by electroporation of the purified ligation reactions into TG1 *E. coli* cells. These electrocompetent and phage‐competent cells are ideal for this process because they have high transformation efficiency, which maintains high library diversity, an F’ episome that enables M13 phage infection, and amber stop suppression, which is permissive for fusion and display (Barderas et al., 2006). Following electroporation, the cells will be plated on antibiotic‐selective medium and incubated overnight, after which the colonies that grow will be harvested and preserved in SOC medium and glycerol for safe storage at –80°C. Collectively, these *E. coli* libraries should contain all the VHH sequences generated by the nanomice in response to the antigen of interest and can be pooled and screened via the phage‐based panning method described in Basic Protocol 7.

### Materials

SOC medium (see recipe)
Phagemids (from Basic Protocol 5)
TG1 electroporation‐competent *E. coli* cells (Agilent, cat. no. 200123)
2xYT/2% glucose agar plates (see recipe; antibiotic will be plasmid‐dependent)
50% (v/v) glycerol solution (see recipe)

Ice bucket
0.1‐cm electroporation cuvettes (Bio‐Rad, cat. no. 1652089)
10‐, 20‐, 200‐, and 1000‐µl filtered pipette tips and single channel pipettes
MicroPulser electroporator (Bio‐Rad, cat. no.)
Falcon brand 50‐ml conical tubes (Corning, cat. no. 352098)
200‐µl gel‐loading tips (Fisher, cat. no. 02‐707‐181)
Shaking incubator
Microcentrifuge tubes
Cell lifters (Corning, cat. no. 3008)
Screw cap microtubes (Sarstedt, cat. no. 72.694.306)

*NOTE*: If working with three mice per antigen, repeat this protocol for all three blood ligation samples and for the spleen and bone marrow ligation samples, for a total of five TG1 libraries per antigen. Alternatively, all three blood samples can be combined into one TG1 library, but the number of cuvettes, plates, and reagent volumes will need to be tripled.

#### Transformation of vector ligated VHH amplicons into a TG1 phage‐competent E. coli library

1 Thaw a minimum of 15 ml of SOC medium by warming to 37°C. Around 13 ml will be needed on day 1, and ∼50 ml the following day, depending on how many plates are used for the library; 1 ml per plate will be needed on day 2.
2 To a large bin of ice, add 11 electroporation cuvettes. Thaw 900 µl of TG1 *E. coli* cells and the DNA ligation sample from one tissue type from the end of Basic Protocol 5 on the same ice.
  The volume of cells and number of cuvettes needed will depend on the volume and concentration of DNA eluted at the end of ligation cleanup. This protocol will assume eluted volume is 68 µl after combining two columns of ligation reactions eluted in 35 µl each and using 2 µl for quantification. Scale up or down as required. If pooling all three blood samples into one TG1 library, scale up the volumes of all reagents, cuvettes, and plates accordingly.
3 Working on ice, add 72 µl of TG1 cells to each cuvette by pipetting. Mark one cuvette as the negative control. If there are leftover thawed TG1 cells, distribute them amongst some cuvettes, excluding the negative control.
4 Add 6 µl of DNA to each positive cuvette, rounding down from 68 µl/11 cuvettes to account for pipetting error. If there is any leftover DNA at the end, divide it amongst a few cuvettes. Do not add any DNA to the negative control cuvette.
  For optimal transformation of plasmids into E. coli cells, a minimum cell:DNA ratio of 10:1 is needed. This protocol uses a ratio of 12:1, providing some extra room if a few µl of leftover DNA needs to be distributed. Adding a few extra µl of TG1 cells will further increase the ratio and thus not hinder transformation efficiency. Do not exceed the 100 µl maximum capacity of the electroporation cuvette.
5 Starting with the negative control, pulse each cuvette one at a time using the *E. coli* setting; if using the Bio‐Rad MicroPulser electroporator, use “Ec 1”. Wipe any ice or water off each cuvette prior to electroporation.
6 Immediately after electroporating, add 900 µl of SOC medium directly into the cuvette to facilitate cell recovery before proceeding to electroporate the next cuvette.
7 Transfer the contents of the negative control cuvette into one 50‐ml conical tube and combine the contents of the positive cuvettes into a second 50‐ml conical tube.
  Gel‐loading tips can fit into the 1‐mm gap of the cuvettes, making it convenient to retrieve the final 100 to 150 µl over traditional pipette tips.
8 Record the total volume recovered from the positive cuvettes; this value will be needed later to estimate the number of colony forming units (CFU) per ml.
9 Incubate both sets of cells at 37°C for 1 hr, shaking at 200 to 250 rpm. In the meantime, warm up 2xYT/2% glucose agar plates made with antibiotics, also at 37°C. A few min before the hour has elapsed, transfer the plates to a lab bench and label them as is relevant to the samples.
  For one negative control plate, six diluted plates (for step 10), and ∼11 ml of positive TG1 library, 43 plates are needed. Add or remove three plates for every 1 ml above or below 11 ml, respectively. This can be scaled accordingly for larger diameter plates.
10 To estimate the number of CFUs in the library, make stepwise dilutions of the positive cells in SOC medium. Six 2‐fold serial dilutions are sufficient for estimating the growth rate of this library overnight. Begin by adding 300 µl of fresh SOC medium to each of six 1.5‐ml microcentrifuge tubes. Add 300 µl of the positive TG1 cells to the first tube, mix by pipetting, then transfer 300 µl into the second tube. Repeat for the remaining tubes.
11 Pipette 300 µl of the negative control TG1 cells onto one clearly labeled plate. Distribute evenly on the plate using a cell spreader, then discard any remaining negative cells.
12 Plate 300 µl of each serial dilution on to their respective labeled plates in the same manner as the negative control plate, starting with the least concentrated and working up to the most concentrated.
13 Plate the rest of the positive TG1 library at 300 µl per plate.
  For all plates, avoid spreading the cell‐SOC solution all the way to the edge of the plate to facilitate colony collection the following day.
14 Leave the plates on the benchtop for 15 to 30 min to dry, then flip over to prevent condensation from accumulating directly on the cells. Incubate all plates at 37°C overnight.
15 To calculate CFUs/ml, count the number of colonies on each dilution plate, multiply that number by the dilution factor, then divide by the volume of cells pipetted onto each plate.
  For example, if 10 colonies grew on the 2^−6^ dilution plate with 300 µl pipetted onto each plate, 10 colonies × 2^6^ ÷ 0.3 ml = 2133 or 2.133 ×  10^3^ CFUs/ml. To get total CFUs, multiply by the volume of cells in SOC prior to plating. For 11 ml plated, 10 colonies × 2^6^ ÷ 0.3 ml × 11 ml = 23,467, or 2.35 × 10^4^ CFUs. Repeat for all countable plates to get an average CFU for the library.
  If the undiluted plates have low enough growth for colonies to be countable, count three undiluted plates instead and take the average of those numbers multiplied by the total library volume. However, it is common for only the 3 to 4 most diluted plates to be countable.
16 To each plate apart from the negative control, add 0.5 to 1 ml of SOC medium depending on colony density, scrape the colonies using a cell lifter, and pipette the suspension into a new 50‐ml tube.
17 Once the cells have been collected from all plates, add 50% (v/v) glycerol solution to the tube to a final concentration of 15% to 20%, mix with the cells via gentle pipetting, then aliquot into as many screw cap microtubes as needed. This is the VHH amplicon TG1 library. Store at –80°C until ready to proceed to phage panning or other analysis steps.

## Basic Protocol 7 ANTIGEN‐DRIVEN VHH SELECTION USING PHAGE DISPLAY

**Figure 8:**
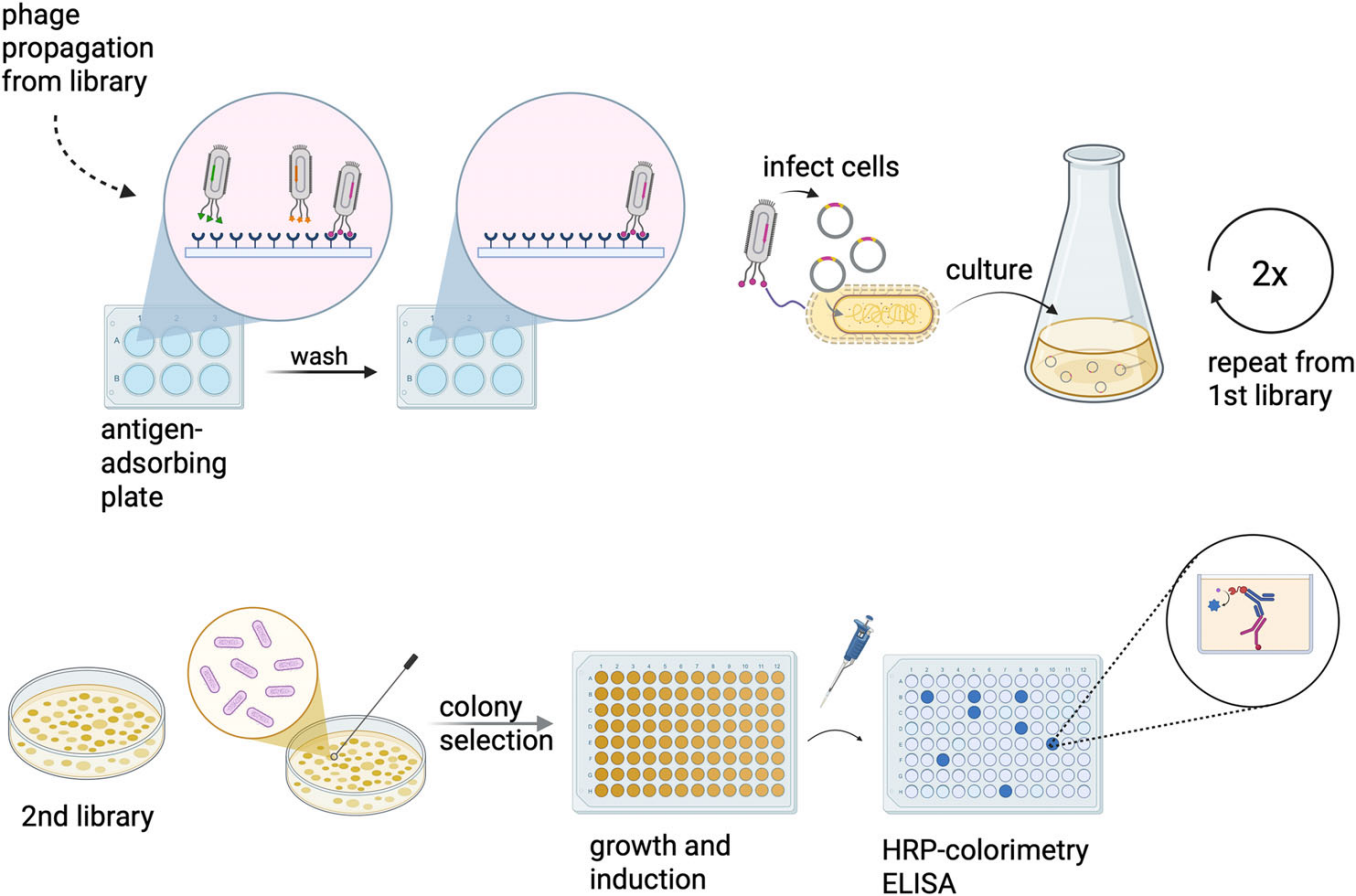
A graphical overview depicting key concepts in the phage biopanning (Basic Protocol 7) and ELISA selection (Basic Protocol 8) assays. Phage libraries are amplified twice against sequentially decreased antigen load; colonies plated from second‐round panning are picked to be evaluated in ELISA. Created in BioRender; A. Huffaker (2026).

The library created in Basic Protocol 6 encodes all VHH sequences isolated from immunized nanomice. To begin identifying antigen‐specific nanobodies in Basic Protocol 7, the VHH phagemid library in amber‐suppressor *E. coli* (TG1) is grown to mid‐log phase and infected with VCSM13 helper phage. This facilitates the assembly of bacteriophages displaying VHH‐pIII fusion proteins (Ledsgaard et al., 2018). These phages are precipitated and applied to immobilized antigen, washed to remove weak and non‐specific binders, and the strongly bound phages are acid eluted. The eluted phages then infect fresh ER2738 cells to enrich the functional VHH pool. This biopanning process is performed twice with sequentially decreasing antigen concentrations to select for high‐affinity candidates. The resulting enriched libraries are subsequently processed for single‐colony ELISA screening in Basic Protocol 8 (Fig. 8).

*CAUTION*: Phages can contaminate other bacteria. Keep all phage‐containing cultures separate; incubators should not be shared in this protocol. Use germicidal bleach (10%) to disinfect all used glassware and materials before washing or autoclaving for reuse. Use filtered tips on pipettes.

### Materials

SOC medium (see recipe)
Carbenicillin 1000× stock solution (see recipe)
TG1 VHH library (created in Basic Protocol 6), 0.1–1 × 10^6^/ml CFUs, 200 to 500 µl total
2xYT growth medium, 0.1% (w/v) glucose supplemented with carbenicillin (1×) and kanamycin (1×), 100 ml total (see recipe)
Kanamycin solution (1000×) (Sigma, cat. no. K0254‐20ML)
VCSM13 helper phage (Agilent, cat. no. 200251)
4 ml of 10 µg/ml of antigen protein of interest in PBS
1× PBS (see recipe)
1% (w/v) BSA solution in 1× PBS (see recipe)
20% (w/v) PEG in 2.5 M NaCl solution (see recipe)
2xYT growth medium with 2% (w/v) glucose and tetracycline (see recipe)
1× PBS, sterile (Invitrogen, cat. no. 10010049)
ER2738 *E. coli* cells (Amid BioSciences, cat. no. ER‐20)
1% (w/v) BSA solution in PBST (0.1% Tween 20) (see recipe)
1× PBST (0.1% Tween 20) (see recipe)
0.2 M glycine solution, pH 2.2 (see recipe)
1 M Tris solution, pH 9.1 (see recipe)
2xYT agar growth plates, 2% (w/v) glucose, antibiotic (see recipe)
50% (v/v) glycerol solution (see recipe)

Five to six 250‐ml Erlenmeyer flasks
10‐, 20‐, 200‐, and 1000‐µl filtered pipette tips and single channel pipettes
Shaking incubator
Spectrophotometer at 600 nm and appropriate cuvettes
Centrifuge with 50‐ml conical tube rotor, 4°C
6‐well Nunc treated plate (Fisher, cat. no. 14‐832‐11)
Parafilm (Amcor, cat. no. HS234526B)
Ice container and ice
5‐, 10‐, and 25‐ml serological pipettes
Pipette controller or pipette gun
1.5‐ml microcentrifuge tubes
Benchtop centrifuge, 4°C
Benchtop rocker
Vacuum trap flask
Cell spreader (Fisher, cat. no. 14‐665‐231)
Cell lifter (Corning, cat. no. 3008)
Screw cap microtubes (Sarstedt, cat. no. 72.694.306)

#### Phage biopanning, round 1, day 1

##### Amplification of VHH E. coli library

1 Prepare 100 ml of SOC medium supplemented with 100 µg/ml (1×) carbenicillin in a 250‐ml Erlenmeyer flask.
  a. Add 100 µl carbenicillin 1000× stock solution to 100 ml SOC medium. Mix to combine.
  b. Remove 1 ml as a blank for the spectrophotometer.
2 Add 200 to 500 µl of VHH TG1 library to the SOC medium.
  The VHH TG1 library is assumed to be homogenous, meaning the full range of diversity is present in each aliquot. This library contains all amplified VHH sequences present in the animal, including those not generated by immunization. The phagemid vector in this protocol contains a carbenicillin resistance gene; this may differ depending on the vector used. At this stage, samples from all TG1 libraries created for a given antigen are used.
3 Grow the culture to an OD600 between 0.4 and 0.7 at 37°C with shaking. This will take 90 to 120 min.
4 While growing, prepare 100 ml of 2xYT/0.1% glucose growth medium supplemented with kanamycin and carbenicillin (1×).
  Opting to remove a small amount and grow as a separate overnight culture to extract library DNA can be useful if evaluating entire library sequence datasets and will reduce bias in sequence selection. If doing this, begin second culture for DNA extraction before adding phages.
5 Add 250 to 500 µl VCSM13 helper phage at a minimum of 0.5 × 10^11^ pfu; extra phage is better.
  This culture should be grown away from other cultures to avoid phage contamination. The vendor‐supplied phages are ∼10^11^ pfu/ml. These phages can be propagated to increase pfu/ml; the phage stocks used in this protocol were stocked at 10^14^ pfu/ml. To propagate phage stocks, infect a naive F+ E. coli strain, such as ER2738, with phages. Follow the precipitation steps as outlined in this panning protocol; scale up volume as needed. Check the infectivity of any prepared stocks by performing a plaque‐forming unit assay; refer to NEB's protocol for detailed instructions (see Internet Resources).
6 Incubate the culture at 37°C for 30 min without shaking. After 30 min have elapsed, resume shaking for an additional 90 min.
7 In the meantime, cool a rotor to 4°C.
8 Pellet the infected bacterial culture for 10 min at 8000 × *g*, 4°C. While the culture is being centrifuged, cool an incubator to 30°C.
9 Dispose of the supernatant and resuspend the pellet in 100 ml of 2xYT/0.1% glucose/carbenicillin (1×)/kanamycin.
10 Grow the resuspended culture overnight at 30°C with shaking to allow the phages to continue infection and propagation.
  The phagemid library is resistant to carbenicillin (phagemid plasmid‐dependent) and the helper phage confers kanamycin resistance. The double antibiotic medium ensures that the only cells able to grow are cells infected with phage that are also holding a copy of the VHH library. These infected cells will grow and propagate phage virions to precipitate the following day.

###### Nunc plate coating

11 Dilute the antigen protein of choice to 10 µg/ml in 1× PBS or alternative coating buffer.
  Do not use a Tris‐based buffer, as Tris can inhibit the enzymatic amine‐amine conjugation required for the ELISA signal.
12 Add 4 ml of this dilution to one well on the Nunc plate.
13 Add 4 ml of 1% (w/v) BSA solution in 1× PBS to one well. This will be a negative selection well used to deplete the phage pool of off‐target binders. Store the Nunc plate at 4°C overnight.
  Alternatively, non‐specific phage virions can be functionally reduced in quantity by incubating in a blocking solution of milk or BSA, then transferring the blocked virion suspension to the antigen‐containing well. This may reduce the loss of effectively binding phages that may have been bound and immobilized to the BSA‐containing well. Refer to Chapter 8, subheading 3.8 of Patrick Chames (ed.), Antibody Engineering: Methods and Protocols, Second Edition, Methods in Molecular Biology, vol. 907, for a detailed protocol (Vincke et al., 2012).
14 Prepare a 20% (w/v) PEG in 2.5 M NaCl solution as described in recipes. Store at 4°C.
15 Set aside ∼10 ml of sterilized 1× PBS to cool at 4°C.

###### ER2738 culture

16 Prepare 100 ml of 2xYT/2% glucose growth medium supplemented with tetracycline (1×) and add 200 µl of ER2738 competent cells. Grow overnight at 37°C with shaking.

#### Phage biopanning, round 1, day 2

Remove the ER2738 overnight culture from the incubator the next morning and set aside.

##### Isolation and precipitation of phage virions

17 Cool a rotor and centrifuge to 4°C to pellet 100 ml of infected culture.
18 Transfer the infected bacterial culture into tubes or bottles that can withstand centrifugation at high speeds.
  For example, Falcon brand 50‐ml conical tubes can withstand high speeds, while some other brands will break. Reusable, autoclavable bottles also work well.
19 Balance the tubes or bottles and seal the lids with Parafilm. Pellet culture 10 min at 8000 × *g*, 4°C. Retain the supernatant; this contains soluble and differentiated phage virions.
20 Divide the supernatant amongst four new 50‐ml tubes, chilled on ice. Each tube should have ∼25 ml supernatant.
21 Add ∼15 ml of cold 20% (w/v) PEG in 2.5 M NaCl solution to each tube and gently invert several times to mix. Balance the tubes and wrap the lid with Parafilm.
22 Incubate on ice for 1 to 2 hr without agitation. A faint precipitate may appear, or the liquid may become cloudy.
  The supernatant does not always change appearance; this is not reason for concern.
23 Spin down the supernatant/PEG mixture for 20 min at 20,000 × *g*, 4°C. Align the tubes in the rotor so the clear side is facing out.
24 Gently decant the supernatant.
  A feathery, whitish pellet should be seen alongside the wall of the tube. These are the virions, precipitated by PEG.
25 Resuspend the virion pellets in 4 ml of sterile, cold PBS. Residual liquid will add ∼1 ml, for a final volume of ∼5 ml.
  Resuspend the virions by washing down the sides of the tubes with PBS. The suspension will be hazy and whitish yellow. Keep all materials on ice where possible. Use a 5‐ml serological pipette or P1000 pipette with filtered tips.
26 Transfer the PBS‐resuspended virions into five 1.5‐ml microcentrifuge tubes.
27 Add 200 µl of cold 20% (w/v) PEG in 2.5 M NaCl solution to each tube and invert to mix.
28 Incubate on ice at 4°C for 1 hr.
29 While the virions are incubating, cool a benchtop microcentrifuge to 4°C.
30 Pellet the virions by centrifugation for 10 min at 17,000 × *g*, 4°C.
31 Carefully remove and dispose of the supernatant with a P1000 pipette with filtered tips. Do not disturb the pellet on the bottom and sides of the tubes. The pellet will likely be slightly tan with a white streak up the side of the tube.
32 Spin 1 min at 17,000 × *g*, 4°C, then gently remove any residual liquid.
33 Resuspend all virion pellets in cold, sterile PBS for a total volume of 1 ml.
  These pellets can be difficult to resuspend. Resuspending each tube individually with 200 µl PBS per tube and then pooling is effective. After removing the suspension, briefly spin down each tube to collect any remaining liquid.
34 Pool the virions into one tube and centrifuge 5 min at 17,000 × *g*, 4°C.
  Balance with another 1.5‐ml tube containing an equivalent volume of water.
35 Transfer the supernatant into a fresh tube.
  These are the phage virion and should represent all sequences from the TG1 library. Any remaining pellet in the tube is bacteria that has persisted and can be safely discarded. The virion suspension will likely have a slightly hazy, white‐yellow appearance. Virions can be stored at 4°C for several months; longer term storage is recommended at –20° to –80°C with up to 50% glycerol (Modafferi et al., 2025). To measure library‐specific virion infectivity using a colony‐forming unit assay, begin by serially diluting the prepared phage library. Next, incubate the diluted library with mid‐log phase ER2738 cells in LB medium supplemented with tetracycline at 37°C for 30 min without shaking. Following incubation, plate the mixture onto carbenicillin‐containing agar plates and allow the cells to grow overnight. Finally, calculate the colony‐forming units per milliliter as described in Basic Protocol 6.

###### Panning

36 Remove the 1% (w/v) BSA solution in 1× PBS from the Nunc plate negative selection well and discard.
37 Add 500 µl of phages into the negative selection well.
  The remaining ∼500 µl of phages can be stored and used for panning other antigens in the same library. These have not been depleted of non‐specific binding sequences.
38 Remove the antigen coating solution from the positive selection well using a pipette and discard. Avoid touching the surface of the plate.
39 Block the positive well with 4 ml of fresh 1% (w/v) BSA solution in PBST (0.1% Tween 20).
40 Rock the plate at room temperature for 1 to 2 hr.
41 Remove the blocking solution from the positive well and discard.
42 Transfer 200 µl of virions from the negative selection well into the positive selection well. Add 3.2 ml of % (w/v) BSA solution in 1× PBS to the positive selection well with virions.
  The remaining ∼300 µl of virions in the negative well can be removed and kept at 4°C for short term or –80°C for long‐term storage, to be used in panning for an additional antigen of the same library.
43 Incubate on a rocker at room temperature for 30 min to 1 hr.
44 Remove the supernatant from the positive well and discard.
45 Using a serological pipette and vacuum flask setup, wash the positive well 15 times with 4 ml of 1× PBST (0.1% Tween 20).
46 Add a final 4 ml of 1× PBST (0.1% Tween 20) to the positive well and incubate with gentle agitation for 15 min at 37°C.
47 Remove the 1× PBST (0.1% Tween 20) and wash the positive well once with 4 ml of 1× PBS to remove residual detergent, then discard.
48 Add 1 ml of 0.2 M glycine solution, pH 2.2, to the Nunc plate positive well and incubate at room temperature with rocking or gentle agitation for 10 min. While the plate is incubating, prepare a 1.5‐ml microcentrifuge tube with 150 µl of 1 M Tris, pH 9.1.
49 Transfer the glycine solution from the Nunc plate into the tube containing 150 µl of 1 M Tris, pH 9.1, to neutralize.
50 Pool this neutralized solution with 1 ml of ER2738 culture. The Nunc plate can be discarded.
51 Incubate for 15 min at 37°C.
52 Plate bacterial incubation using cell spreaders on 2xYT/2% glucose agar growth plates supplemented with 1× tetracycline and 1× carbenicillin.
  ER2738 cells carry an F’ episome maintained by tetracycline. Carbenicillin is used to select for plasmid‐containing cells; the appropriate selection antibiotic may differ depending on phagemid vector used.
53 Plate 300 µl per plate; include 10‐fold serial dilutions up to 10^−5^ in triplicate. This will require 15 plates for dilutions and 7 to 8 plates for the remaining undiluted library for a total of ∼22 plates.

#### Phage biopanning, round 1, day 3

54 Calculate CFUs as described in Basic Protocol 6, step 15.
55 Using a cell lifter, scrape all bacterial growth from the medium. Use a P1000 pipette and 0.5 to 1 ml of SOC medium to rinse down the agar and remove the medium and bacteria from the plate. Final volume may vary here but should be ∼10 ml. Add 3/7 the suspension volume of a 50% (v/v) glycerol solution for 15% final glycerol concentration.
56 Aliquot the suspension into clearly labeled screw cap microtubes and freeze at –80°C. This is the first‐round panning library.
  It is possible to begin a second‐round panning culture before storing the library; this timing may not always be feasible. Be very precise in labeling of tubes. Include animal, date, antigen, and panning round number. This library will be used to begin the starting culture for the second round of panning. Ideally, this panned library contains fewer sequences than the previous library and the sequences translate to nanobodies with proven binding ability.
  If starting a culture for second‐round panning on the same day as storing the first‐round library, start this culture only after the entire library has been scraped from the plates and pooled; do not take a scrape from one plate. Use the pool for the second‐round starting culture.

#### Phage biopanning, round 2, day 1

##### Amplification of first‐round panning library

57 As was done in round 1 (Basic Protocol 7, step 1), prepare 100 ml of SOC medium supplemented with 1× carbenicillin and remove 1 ml as blank for spectrophotometer.
58 Inoculate the SOC medium with 500 µl of the first‐round panning library and grow until OD600 = ∼0.6.
  Initial OD after inoculation should be in the 0.15 to 0.3 range; expect 90 to 120 min for growth. This may be slower than expected when compared to other E. coli growth rates.
59 Add 250 µl of fresh VCSM13 helper phage at a minimum 0.5 × 10^11^ pfu/ml. More is better.
  This should be a fresh aliquot of phages, not from phages used at any point during the first round. The goal is to introduce new helper phages that can propagate only the selected sequences retained in the library from the first round, not confound the second‐round screening with sequences that did not survive the first round of screening. Incorporation of antibody fragment to the endogenous pIII viral capsid protein is inefficient (Hoogenboom et al., 1998); having an excess of phage may help display coverage. Phages from the vendor are ∼10^11^ pfu/ml. The phage stocks used in this protocol are at ∼10^14^ pfu/ml.
60 Incubate the infected culture for 30 min at 37°C without shaking.
61 Prepare 100 ml of 2xYT/0.1% glucose growth medium supplemented with 1× carbenicillin and kanamycin. The appropriate antibiotics may vary depending on the type of vector and phage used.
62 After 30 min have elapsed, resume shaking for 90 min at 37°C.
63 Pellet the culture for 10 min at 8000 × *g*, 4°C, then discard the supernatant. Set an incubator to 30°C.
64 Resuspend the pellet in the prepared 100 ml of 2xYT/0.1% glucose growth medium with 1× carbenicillin and kanamycin.
65 Grow overnight at 30°C with shaking in a 250‐ml Erlenmeyer flask.

###### Antigen coating on Nunc plate

66 Coat one well of a 6‐well Nunc plate with 1 ml PBS containing 1 µg/ml antigen of interest. This is the positive selection well.
67 Coat another well with 1% (w/v) BSA solution in 1× PBS. This is the negative selection well.
  See note in basic protocol 7, step 13 for an alternative negative selection strategy. Using a different blocking solution than used in round 1 panning may additionally reduce cross‐reactive candidates while maintaining a high level of sequence diversity.
68 Store at 4°C overnight with the accompanying lid.

###### ER2738 culture

69 Prepare 100 ml of 2xYT/2% glucose growth medium with 1× tetracycline.
70 Add an aliquot of ER2738 competent cells to the prepared 2xYT medium and grow overnight at 37°C with shaking.

#### Phage biopanning, round 2, day 2

Remove ER2738 overnight culture from incubator the next morning and set aside.

##### Phage virion isolation and precipitation

71 Spin down the infected bacterial culture for 10 min at 8000 × *g*, 4°C.
72 Divide the supernatant among four 50‐ml conical Falcon tubes, on ice. Each tube should have ∼25 ml supernatant.
73 Add 15 ml of 20% (w/v) PEG in 2.5 M NaCl solution to each tube. Balance the tubes and seal with Parafilm.
74 Incubate on ice at 4°C for 1 to 2 hr.
  A faint precipitate or cloudiness may appear. The supernatant may not change appearances; often it does not. We usually do not notice any visual change; this has not correlated to poor phage yield.
75 Spin down the tubes for 20 min at 20,000 × *g*, 4°C. Arrange tubes so that the clear side is facing out.
76 Carefully remove the supernatant and discard.
  The tubes should have a faint white streak down the wall of the tube; these are precipitated phage virions.
77 Resuspend the virion pellet in 4 ml total volume cold, sterile 1× PBS.
78 Transfer the suspension into four to five 1.5‐ml tubes and add 200 µl of 20% (w/v) PEG in 2.5 M NaCl solution per ml.
79 Incubate on ice at 4°C for 1 hr.
80 In the meantime, cool a benchtop centrifuge to 4°C.
81 Pellet the virions by centrifugation for 10 min at 17,000 × *g*, 4°C.
82 Gently remove the supernatant and discard.
83 Spin the tubes for 1 min at 17,000 × *g*, 4°C, and remove any residual liquid.
84 Resuspend the virions in 1 ml total volume of cold, sterile PBS.
  Individually resuspending each tube with 200 µl PBS then pooling is most effective.
85 Pool the suspensions together and centrifuge for 5 min at 17,000 × *g*, 4°C.
86 Transfer the supernatant into a new tube. These are the phage virions amplified from the first library.
  As described in the comments on step 36, there may be a small pellet present after the final 5‐min spin. This is pelleted bacteria. The phage virions are soluble and retained in the supernatant. As with first‐round panning, the second‐round panning will take several hours. If not possible to pan the same day, store phages at 4°C for short to medium term, and at –80°C supplemented with 15% to 20% glycerol for longer term storage. Measure virion infectivity by performing a plaque‐forming unit assay on antibiotic‐free top agar plates, or colony‐forming unit assay on carbenicillin agar plates. Solubilization of virions reduces infectivity. New England Biolabs has detailed protocols on plaque‐formation assays for a similar phage strain (see Internet Resources).

###### Panning

87 Remove the blocking solution 1% (w/v) BSA solution in 1× PBS from the negative well of the Nunc plate and discard.
88 Add 500 µl of phage virions to the negative selection well.
  The remaining ∼500 µl phages can be used to repeat second‐round panning against only the same library and antigen, if necessary. They will need to be depleted against a negative antigen first.
89 Remove the antigen solution from the positive selection well and discard.
90 Add 2 ml of 1% (w/v) BSA solution in PBST (0.1% Tween 20) to the positive selection well.
91 Rock the plate at room temperature for 1 hr.
92 Remove the blocking solution (BSA solution in PBST) from the positive selection well.
93 Add 500 µl of 1% (w/v) BSA solution in 1× PBS to the positive well of the Nunc plate.
94 Transfer 20 µl of phage virions from the negative selection well to the positive selection well.
  The remaining ∼480 µl of phage virions can be reused for the same library and antigen. These have already been enriched against a specific antigen and depleted of nonspecific sequences.
95 Incubate the phages in the positive selection well for 10 min at room temperature while rocking.
96 Remove the supernatant from the positive selection well and discard.
97 Wash the positive selection well 10 times with 3 ml of 1× PBST (0.1% Tween 20) using a serological pipette and vacuum flask. Avoid touching the surface of the plate.
98 Add 2.5 ml of 1× PBST (0.1% Tween 20) to the antigen‐positive well and incubate for 1 hr at 37°C with gentle agitation.
99 Remove 1× PBST (0.1% Tween 20) and wash once with 3 ml PBS to remove residual Tween 20.
100 Add 500 µl of 0.2 M glycine solution, pH 2.2, to the positive selection well and incubate at room temperature with gentle rocking for 10 min.
101 Meanwhile, add 75 µl of 1 M Tris, pH 9.1, to a 1.5‐ml centrifuge tube and set aside.
102 Transfer the glycine solution from the Nunc plate to the tube containing Tris, pH 9.1, to neutralize.
103 Mix and pool the neutralized glycine/Tris solution with 500 µl ER2738 culture.
104 Incubate for 15 min at 37°C with gentle shaking.
105 Plate 300 µl bacteria on 2xYT/2% glucose/tetracycline/carbenicillin agar medium plates.
  a. Include serial dilutions up to 10^−5^ in triplicate by preparing five 1.5‐ml tubes with 900 µl growth medium.
  b. Transfer 100 µl of culture into the first dilution tube and mix by pipetting.
  c. Sequentially transfer 100 µl from the first tube into the second, the second into the third, and so on. The undiluted culture should be ∼1 ml; each dilution tube will have 900 µl total.
  d. This will take 18 to 20 plates in total, including dilutions.
106 Grow plates overnight at 37°C.

#### Phage biopanning, round 2, day 3

107 Calculate CFUs of second round library.
  Calculate CFUs/ml by counting the number of colonies, dividing by the volume plated (ml), and multiplying by the dilution factor. Multiply this number by the total culture volume to calculate total CFUs. Example: a 10^−5^ dilution plate has 45 colonies from 300 µl plated culture. The CFUs/ml would be (45 × 10^5^)/(0.3)= 1.5 × 10^7^ CFUs/ml. Before plating, if the total volume of the culture was 1.5 ml, the total CFUs would be (1.5)(1.5 × 10^7^) = 2.25 × 10^7^. Count plates that have between 20 to 200 colonies; counts outside of this range may not accurately reflect the library. Count all three plates for the appropriate dilution and average.
108 Collect all bacterial lawns by adding SOC medium and scraping. Use a P1000 pipette to add 0.5 to 1 ml SOC to the growth plates, scrape down all growth with a cell lifter and use the pipette to transfer the suspended cells to a 50‐ml tube. Total volume of the final suspension should be ∼10 ml.
109 Set aside any plates with discrete colonies; these will be picked for future ELISA screens. Store upside down at 4°C.
  Plates that are fully picked of colonies may be discarded; plates that are too dense to pick colonies from should be scraped and stored as described in steps 109 to 112. Plates that are partially picked may be scraped and stored in the same manner.
110 Aliquot into screw cap microtubes and supplement with 50% (v/v) glycerol solution for a final concentration of 15%, as described in Basic Protocol 7, step 58.
111 Store the libraries at –80°C.
  Successful conclusion of this protocol will result in two sequentially enriched VHH libraries stored in ER2738 E. coli and several agar plates containing discrete colonies, each representing one genotype, to pick and evaluate in ELISA. Expected CFU for second‐round panning libraries is ∼10^7^ CFU/ml ± one order of magnitude.
  Label all tubes accurately and include as much information as possible to avoid mixing up libraries and samples, such as organism, date, antigen, and panning round. Colonies on diluted plates will be individually selected and used to inoculate 96‐well plates to evaluate binding at an individual sequence level. Positive results from downstream ELISAs may be good candidates for cloning and expression.
  The dilution plates should have numerous discrete colonies. This is required to proceed with Basic Protocol 8. If there are not discrete colonies (i.e., lawns) or a prohibitively low number of discrete colonies, grow a small culture of second‐round panning library in 2xYT with 2% glucose, 1× tetracycline and 1× carbenicillin to OD600 ∼0.6; serially dilute this and plate as described in step 110. This should result in appropriate colonies to proceed with Basic Protocol 8.

## Basic Protocol 8 SINGLE COLONY VHH ELISA SCREENING

In Basic Protocol 8, enriched TG1 clones from the phage panning libraries made in Basic Protocol 7 will be tested to identify discrete sequences of specific candidate nanobodies. The expectation is that after two rounds of phage panning, the diversity of the remaining VHH sequences decreases whereas sequences of antigen‐specific nanobodies become enriched. However, the panned libraries created in Basic Protocol 7 still contain thousands of sequences with variable binding capabilities. Basic Protocol 8 describes an enzyme‐linked immunosorbent assay (ELISA) using the antigen as plate‐immobilized bait protein, while nanobodies expressed and secreted from selected ER2738 clones containing a phagemid vector serve as primary antibodies. VHH‐antigen binding is then detected using a cocktail of anti‐VHH horseradish peroxidase (HRP)‐coupled secondary antibodies in a colorimetric assay. Conclusion of this protocol results in qualitative evaluation of individual nanobodies as expressed by distinct ER2738 clones. Promising clones are expected to proceed to sequence determination and functional testing.

***CAUTION***: Exercise caution and wear appropriate PPE when using hydrochloric acid; this is a highly corrosive substance that can damage skin on contact and can create irritating fumes. Thoroughly rinse all labware in contact with hydrochloric acid with water before washing or autoclaving.

### Materials

Terrific broth (TB), supplemented with 2% glucose, tetracycline, and carbenicillin (see recipe)
Dilution plates (from panning round 2 of Basic Protocol 7)
50% (v/v) glycerol solution (see recipe)
TB, supplemented with tetracycline, carbenicillin, and 10 mM IPTG (see recipe)
1 µg/ml of antigen protein of interest in PBS, 4°C
4% milk in PBST (1% Tween 20) (see recipe)
1× PBST (1% Tween 20) (see recipe)
VHH‐HRP secondary antibody cocktail for ELISA (see recipe)
TMB (3,3’,5,5’‐tetramethylbenzidine) HRP substrate (VWR, cat. no. TMSK‐1000‐01)
1 M HCl to quench reaction (see recipe)

Generic 96‐well plate (Fisher, cat. no. FB012931)
2‐ to 200‐µl multichannel pipettes
Reagent reservoirs (Fisher, cat. no. 4871)
Sterilized toothpicks to pick colonies (Amazon, ASIN‐B0BR4QFGVR)
Breathable plate seals (Southern Labware, cat. no. BS‐25)
Shaking incubator
Deep‐well, V‐bottom 96‐well plate (Fisher, cat. no. AB‐0765)
96‐well ELISA treated plate (Fisher, cat. no. 21‐377‐203)
Deep‐well plate centrifuge
PCR film 96‐well plate seals (Thermo, cat. no. AB‐0558)
Black 15‐ml conical tubes (Fisher, cat. no. HS4428)
10‐, 20‐, 200‐, and 1000‐µl filtered pipette tips and single channel pipettes
96‐well plate reader at 450 nm

#### Single‐colony ELISA selection

This protocol utilizes three distinct types of 96‐well plates. It is critical to use the proper plates; pay close attention to which plate is being used and referenced at each step.

##### Single‐colony ELISA selection day 1

1 Prepare TB/2% glucose growth medium supplemented with 1× carbenicillin and 1× tetracycline and fill 150 µl in each well of a generic 96‐well plate using a multichannel pipette and reagent reservoirs. This is the master plate. Each plate will require ∼17 ml medium (slight excess).
2 Using sterile toothpicks, carefully pick colonies from second round panning growth plates into single wells of the master plate. Pick the most discrete colonies and do not cross‐contaminate. Leave wells E12 to H12 blank; these will serve as negative controls.
  Establish a system that allows for tracking progress through the plate to avoid double‐inoculation of a well; e.g., inoculate all wells in a row and leave the toothpicks in the wells of the row. Once the row has been completed, remove all but the last toothpick in the row and use that as a guide to start the next row.
3 Cover the plate with a breathable seal and incubate overnight at 37°C with shaking.
4 Repeat as needed based on how many colonies are planned for screening.
  It is not advisable to exceed eight plates per person conducting this protocol.

###### Single‐colony ELISA selection day 2

5 Prepare TB/2% glucose growth medium with 1× carbenicillin and 1× tetracycline and add 288 µl per well in a deep‐well, v‐bottom plate. This is the induction plate.
  This will require ∼30 ml per plate. Each master plate needs a corresponding induction plate.
6 Using a multichannel pipette, inoculate each well of the induction plate with 3.2 µl of cultures from the corresponding master plate.
  Do not reuse tips, do not cross‐contaminate. Match well ID from master plate to induction plate. Successful hits must be able to be properly identified on the master plate. If working from a frozen master plate, use a multichannel pipette or a 96‐well replicator to stab the frozen wells and use these tips to inoculate a deep‐well induction plate. Avoid completely thawing the master plate.
7 Cover the induction plate with a new breathable seal and shake at 37°C for 4 hr.
8 While the induction plates are growing, prepare the master plates for storage by adding 60 µl sterile 50% (v/v) glycerol solution to each well.
  Seal the plate with a PCR film and store at –80°C. Label the plate with the antigen, date, and plate number.
9 After the induction plate has grown for 4 hr, add 128 µl TB/10 mM IPTG growth medium supplemented with 1× carbenicillin and 1× tetracycline to each well of the induction plate. Set incubator temperature to 30°C.
  Final IPTG concentration per well should be ∼3 mM. Prepare ∼15 ml per plate.
10 Seal the induction plate with a fresh breathable seal. If there is a lid available, place that over the seal.
11 Incubate the induction plate overnight at 30°C while gently shaking (∼100 rpm).
12 Coat each well of an ELISA plate with 100 µl of the protein of interest in PBS at 1 µg/ml.
  Each induction plate should have a corresponding ELISA‐treated plate.
13 Store the ELISA plates overnight at 4°C.

###### Single‐colony ELISA selection day 3

14 Discard the contents of the ELISA plate and block each well with 200 µl of 4% milk in PBST (1% Tween 20) at 37°C for 2 hr.
  Each plate will require ∼25 ml, plus an additional 10 to 12 ml.
15 While the ELISA plate is blocking, retrieve the induction plate from the incubator and lyse the cells by several rounds of freezing and thawing. This can be done in a –80°C freezer and incubator; freeze for 15 to 20 min and thaw 30 to 40 min.
  Make note of any failed (wells without growth) or contaminated blank wells.
16 Pellet the cell debris by spinning the induction plate in a deep‐well plate centrifuge for 10 min at 832 × *g*, room temperature.
17 Remove the blocking solution from the ELISA plate and discard. Add 100 µl fresh blocking solution to each well.
18 Transfer 100 µl of supernatant from each well of the induction plate to the corresponding well on the ELISA plate. Use a multichannel pipette. Do not cross contaminate.
  At this point, the induction plate can be disposed of or cleaned and reused if autoclavable. The corresponding cultures are stored on the master plate if they need to be re‐seeded.
19 Seal the ELISA plate with a PCR film and rock at room temperature for 1 hr.
20 Wash each well of the ELISA plate three times with 1× PBST (1% Tween 20).
  Use a plate washer if available or a tray filled with 1× PBST (1% Tween 20). It is efficient to dip the plate in a tray and dump in a sink for the washes but ensure that each well gets filled and no air bubbles are blocking the well. The tradeoff for this step is that significantly more PBST (1% Tween 20) will be used than would be used with alternative washing approaches like a plate washer or manual pipette washing.
21 Wash each well of the ELISA plate three times with 1× PBS to remove Tween 20 residue.
22 Add 100 µl of VHH‐HRP secondary antibody cocktail to each well of the ELISA plate.
23 Seal the plate with a PCR film or similar and rock for 1 hr at room temperature. Near the end of this hour, set aside 6 to 7 ml TMB substrate per ELISA plate in a black tube and allow to equilibrate to room temperature.
24 Remove the antibody cocktail and wash each well of the ELISA plate three times with 1× PBST (1% Tween 20).
25 Wash each well of the ELISA plate three times with PBS.
26 Add 50 µl of TMB substrate to each well of the ELISA plate.
  TMB is light sensitive. Keep the plate in the dark after adding substrate. The reaction should not be allowed to exceed 30 min; check every 5 min. If wells are turning blue quickly, then proceed with the next steps. Wells that reach saturation early will make it difficult to determine relative intensities; this may confound appropriate selection of wells for future analysis.
27 Quench the reaction by adding 100 µl of 1 M HCl to each well.
  This will cause any blue wells to turn yellow. This should be stable for up to 30 min.
28 Read each ELISA plate on a plate reader set to 450 nm.
  Determine criteria for positive results and make note of well number and plate ID. Determine absorbance by subtracting the average absorbance of the negative wells from each test well. Criteria may change depending on results and antigen. An ideal result is several wells with relatively high absorbance at 450 nm (0.5 to 1.0) and a consistent baseline absorbance for non “hit” wells. Some plates will have no “hits”; this is also a common result. Negative wells should be around the same value. Plot signal normalized to negative controls vs well ID to visualize which wells correspond to strong signal.

## Basic Protocol 9 SEQUENCING OF ELISA HITS AND CANDIDATE VHH SEQUENCE IDENTIFICATION

Basic Protocol 9 will describe how to sequence hits that were identified as binders in the ELISAs conducted in Basic Protocol 8 and determine which sequences are optimal for cloning into a nanobody expression vector suitable for bulk purification. Phagemid DNA from positive ELISA hits will be purified and sequenced. The sequences will then be evaluated for enrichment to determine which ones are the best candidates for cloning, expression, and purification.

### Materials

LB with carbenicillin and tetracycline (see recipe)
ELISA master plate(s) (from Basic Protocol 8)
50% (v/v) glycerol solution (see recipe)
QIAprep Spin Miniprep kit (Qiagen, cat. no. 27106)
UltraPure nuclease‐free water (Invitrogen, cat. no. 10977015)
Phagemid vector‐specific forward primer (IDT, 25 nmol, standard purification, diluted to 10 µM; see recipe)
  This should be designed to correspond to the vector used in Basic Protocol 5 for ligation of VHH amplicons; the primer should start roughly around 100 bases before insertion of the VHH either in the promoter or the leader peptide (if present).

5‐ml round‐bottom test tubes (Falcon, cat. no. 352058)
10‐µl non‐filtered pipette tips
10‐, 20‐, 200‐, and 1000‐µl filtered pipette tips and single channel pipettes
Shaking incubator
Screw cap microtubes (Sarstedt, cat. no. 72.694.306)
Nanodrop or similar equipment that can measure nucleic acid concentration
1.5‐ml microcentrifuge tubes
Parafilm (Amcor, cat. no. HS234526B)
UGENE (https://ugene.net/) or a similar program

#### Culturing ELISA hits for Minipreps

1 Inoculate 2.5 ml of LB containing carbenicillin and tetracycline with a stab from the ELISA master plate. Use a 5‐ml round‐bottom tube and a 10‐µl non‐filtered sterile pipette tip for the stab, leaving the tip in the culture.
2 Incubate overnight (16 to 18 hr) with shaking (∼200 rpm) at 37°C.
3 The next day, combine 750 µl of sterile 50% glycerol and 750 µl of overnight culture in a screw cap microtube to make a glycerol stock and store at –80°C.
4 Miniprep 2 ml of the culture using the QIAprep Spin Miniprep kit, according to the manufacturer's instructions (see Internet Resources).
5 Determine the plasmid DNA concentration using a Nanodrop and send to your preferred sequencing vendor for Sanger sequencing using the forward primer specific to the cloning vector.
6 Sequencing reactions should contain 5 to 25 ng of plasmid DNA diluted in up to 10 µl of nuclease‐free water, plus an additional 10 µl of nuclease‐free water, and 5 µl of phagemid vector‐specific forward primer.
  a. Wrap Parafilm around the microcentrifuge caps to prevent sample leaks.
  b. Include the ELISA master plate number and the well ID in the sequence name when placing the order.

#### Sequence alignment and selection for cloning

7 After sequencing results are returned, open the FASTA files (.seq file) in UGENE (see Internet Resources) as follows.
8 Click on “File” and select “Open” from the drop‐down menu.
9 Select multiple FASTA files and click “Open”.
10 A window with the heading “Multiple Sequence Reading Mode” will pop up showing the sequences to be opened. The sequences can only be opened from within the program to display the multiple alignments appropriately.
11 Click the “Join sequences into alignment” radio button. Save the alignment at this point.
12 Click “OK” and the multiple alignment of the sequences will be visible.
13 Starting with the first FASTA sequence, find the first “ATG” nucleotide sequence.
  This should be present if contained in the design of the primer that was created to correspond to the vector used in Basic Protocol 5 for ligation of VHH amplicons; the primer should start around 100 bases before insertion of the VHH either in the promoter or before the leader peptide (if present).
14 Click and hold on the nucleotide right before “A”, then drag the mouse all the way back to the beginning to select those bases.
15 Right‐click the selection and choose “Edit”, then choose “Remove selection”.
  Now this FASTA sequence should start with “ATG”.
16 Repeat this process with the rest of the FASTA sequences.
  Once this is complete, all the sequences should look identical for the first few dozen bases. This is because this initial part of the sequence belongs to the cloning vector.
17 Find the end of the nanobody‐encoding sequence.
  The amino acid sequence at the end of a nanobody is TVSS or TVSA and there several nucleotide combinations that can code for these sequences; however, the following combinations are the most observed:
  a. ACCGTCTCCTCA.
  b. ACAGTCTCCTCG.
  c. ACGGTCTCCTCG.
    Because the middle two nucleotide triplets are the same in each combination, the sequences can be searched using those 6 nt.
18 To search for the nanobody end sequence, click on the tab on the right‐hand side of the window labeled “ACG”, type in “GTCTCC”, and press enter. The sequence will be highlighted. It is usually located around 400 to 450 bp.
19 Starting with the last base of the nanobody end sequence, select all the bases from that point backwards and “Ctrl+C” to copy.
20 Click anywhere in the window to deselect it and “Ctrl+V” to paste.
21 Scroll right until the entire ATG + nanobody sequence has been captured.
22 Repeat for the rest of the FASTA sequences.
  Once finished, select the names of the original imported sequences on the left side of the window (which will select the entire nucleotide sequence) and delete as was done in step 15. This will clean up the window and help ensure that only the correct sequence is being worked with.
  Save this nucleotide sequence alignment file (the tab above the alignment shows as “Multiple alignment [merged_document.aln]”).
23 Select all the sequences then click on the “Actions” drop down menu. Move the cursor over the “Export” selection, then choose “Export amino acid translated alignment”.
24 A pop‐up box with the heading “Export Amino Translation” will appear. Click on the “Export” button at the bottom right of the box. This pop‐up box allows for changing the file format, type of translation, the reading frame, and the export range. Use the default options.
  There are nanobody amino acid signatures that signal both the beginning and the end of a nanobody sequence. There are seven possible starting signatures in the nanomouse (QVQLVES, QLQLVES, QVKLEES, QVQVVES, DVQLVES, EVQVVES, or EVQLVES) and two possible end signatures (TVSS or TVSA). These signatures should be identifiable in the exported translation.
25 Scan the amino acid sequence for premature stop codons. The sequences that come before the nanobody start signature can be disregarded.
  If there are any stop codons within the nanobody amino acid sequence, this nanobody may not be a good candidate for further cloning.
26 Starting from the beginning, delete all amino acids before the starting signature.
27 Scroll across the sequence and this time, look for those that are identical. If any are found, click and hold on the name and drag it right below its matching sequence.
  Save this amino acid sequence alignment file (the tab above the alignment shows as “merged _document_transl [merged_document_transl.aln]”).
  If multiple ELISA hits turn out to be the same sequence, that is indicative that it may be immunologically important. These are excellent candidates for cloning. Unique sequences that were strong ELISA hits are also good candidates. Choose several sequences. VHH sequences that were weak ELISA hits are less desirable, but do not rule them out completely if they are found to be enriched/repeated in the sequencing results. It is not uncommon for nanobodies to have a synergistic effect when combined with others (Mast et al., 2021).

## Basic Protocol 10 GENEBLOCK DESIGN OF CANDIDATE VHH AND GIBSON ASSEMBLY INTO A NANOBODY EXPRESSION VECTOR

This protocol outlines the subcloning of candidate nanobody sequences (the small subset of clones that were identified in Basic Protocol 9) into an expression vector for large batch expression. To expedite the transition from sequence to protein, we use synthetic Open Reading Frames (ORFs) to facilitate rapid, one‐step Gibson Assembly. This strategy bypasses the labor‐intensive constraints of traditional restriction‐digest‐based cloning. The pHEN expression vector employed here was developed from the M13 cloning vector pUC119 (Vieira & Messing, 1987), modified with a PelB leader sequence to enable soluble periplasmic expression and nanobody cloning (Hoogenboom et al., 1991) and a multiple cloning site (MCS), resulting in pHEN4 (Arbabi Ghahroudi et al., 1997), and eventually pHEN6, which further integrated a C‐terminal His_6_‐tag to streamline downstream purification (Conrath et al., 2001). Although the specific pHEN construct used here is not commercially available, numerous functionally equivalent derivatives are accessible. Upon successful assembly, these recombinant vectors are prepared for protein expression as detailed in Basic Protocol 11.

### Materials

pHEN or alternate nanobody expression vector (e.g., Addgene, cat. no. 194591)
Restriction digest enzymes with buffers compatible with cloning into your nanobody expression vector, e.g.:
  10× rCutSmart buffer (New England Biolabs, cat. no. B6004S)
  BstEII‐HF restriction enzyme (New England Biolabs, cat. no. R3162S)
  PstI restriction enzyme (New England Biolabs, cat. no. R0140S)
UltraPure nuclease‐free water (Invitrogen, cat. no. 10977015)
Agarose gel, 1% (see recipe)
NEB 6× DNA loading dye (NEB, cat. no. B7024)
GeneRuler 1 kb DNA ladder (Thermo Scientific, cat. no. SM0311)
Zymoclean Gel DNA Recovery kit (Zymo Research, cat. no. D4008)
Gibson Assembly master mix (New England Biolabs, cat. no. E2611L)
SOC medium (see recipe)
LB agar plates with carbenicillin (see recipe)
MAX Efficiency DH5α competent cells (Invitrogen, cat. no. 18258012)
LB growth medium with carbenicillin (see recipe)
50% (v/v) glycerol solution (see recipe)
QIAprep Spin Miniprep kit (Qiagen, cat. no. 27106)

ApE, A plasmid Editor (https://jorgensen.biology.utah.edu/wayned/ape/) or a similar program
10‐, 20‐, 200‐, and 1000‐µl filtered pipette tips and single channel pipettes
1.5‐ml microcentrifuge tubes
Ice container with ice
Heat block or water bath for microcentrifuge tubes
Gel electrophoresis system
UV imager for DNA gels
Nanodrop or similar equipment that can measure nucleic acid concentration
Microcentrifuge
Vortex
5‐ml tubes
Shaking incubator
UGENE (https://ugene.net/) or a similar program

#### Geneblock design and ordering

Make a new ApE file for each new geneblock to be ordered. This will provide a reference sequence against which to compare whole plasmid sequencing data after Gibson Assembly. If there is a construct with a nanobody sequence already inserted (which is strongly recommended over an empty vector), it serves as a guide to where to modify the construct and insert the new nanobody sequence. We have listed a reference pHEN‐nanobody vector in the materials section that is commercially available. The geneblock and the new ApE file will be created in parallel in this protocol.

**Figure 9:**
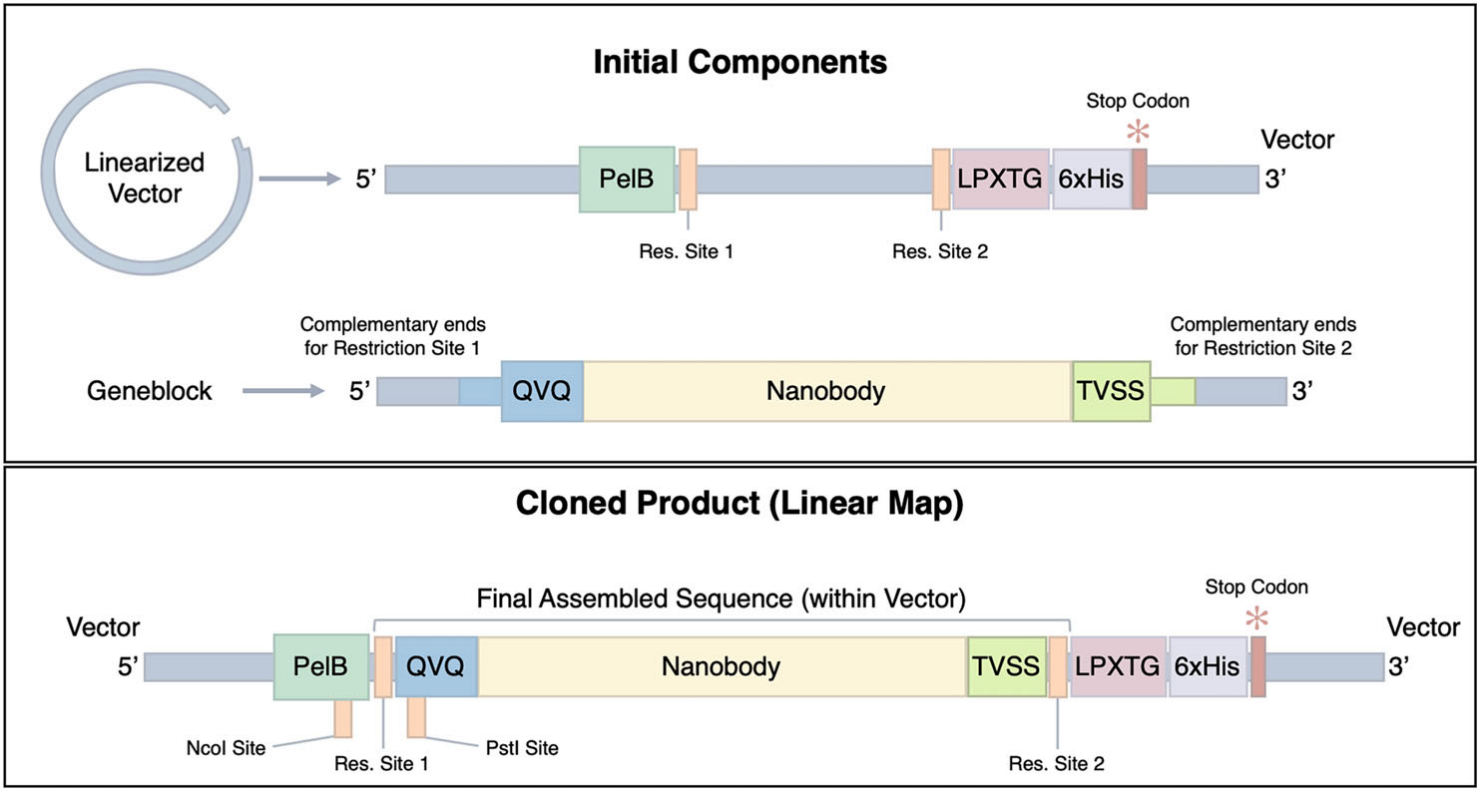
A schematic of the linearized expression vector, a geneblock harboring a nanobody sequence for cloning, and the assembled product upon completion of Gibson Assembly. NcoI is a useful restriction site that is present in PelB and PstI is a site that can be utilized in Frame 1. LPXTG is a Sortase A recognition sequence commonly used for the site‐specific bioconjugation of nanobodies.

1 Open the nanobody expression vector in ApE, preferably a GenBank (.gbk) file (see Internet Resources).
  Obtain the whole plasmid sequence file of the expression vector for this activity.
2 Highlight the PelB sequence in the nanobody expression vector. This should be annotated already in ApE when using a GenBank file, but if it is not, search for the following sequence (command F):
  ATGAAATACCTATTGCCTACGGCAGCCGCTGGATTGTTATTACTCGCGGCCCAGCCGGCCATGGCC
3 If it is not pre‐annotated, annotate it now by highlighting the PelB sequence with the mouse cursor and then go to the features section, select “new feature” and name it PelB.
4 While the PelB is highlighted, starting at the ATG of the PelB, use “command T” or “control T” to translate it into a single amino acid sequence and this sequence should be seen:
  MKYLLPTAAAGLLLLAAQPAMA
5 The M start of the PelB should be visible as well as the MA that is the start for a nanobody; if the MA is not visible, highlight the PelB sequence plus 20 more bases to the right and translate again. If working with a plasmid that already has a nanobody sequence entered, which is recommended, locate an MAQVQ or other combination of the MA‐nanobody starting signature.
6 Use the “Enzymes” pull down menu to initiate “Enzyme Selector” when the selector window is open, select for all unique enzymes, and perform action highlight. Then close the window.
7 All the unique enzymes should be highlighted on the ape file. Find a unique enzyme that is near the end of the PelB sequence and before where the new nanobody sequence will be inserted. Some pHEN vectors have multiple unique sites in this region for ease of cloning.
8 Highlight this restriction site and name it as a new feature e.g., “first cloning restriction site”.
9 Go back to the enzyme selector tool, deselect all, and close.
10 When returned to the sequence window, the only restriction enzyme site highlighted should be the new feature that was made.
11 Find the restriction site that is near the end of the nanobody sequence. Use the enzyme selector tool to highlight all the unique restriction sites and locate the one that is present in or near a TVS ending signature, it likely will be 300 to 500 bases downstream of the PelB sequence.
12 To tell if this has been located, highlight the sequence starting with the ATG of the PelB and cover the spread of bases all the way to the putative restriction site, then use the translate tool again to assure that the PelB translation is correct, a nanobody sequence can be seen as well as the TVS ending signature. If this is accurate, then annotate this restriction site as well for cloning.
13 The new nanobody needs to fit between the restriction sites where appropriate (Fig. 9). The following steps will describe this process. Keep the full vector ApE file open while working on the next steps.

14 Open a blank word processor document. While working through these steps, include as many details as possible in this document.
15 Paste the new nanobody nucleotide sequence into the word document. This was identified in Basic Protocol 9.
  Make notes in the document about what antigen this sequence targets, and any relevant plasmid or glycerol stocks that correspond to this sequence.
16 Translate the nucleotide sequence from the word document by pasting it into a new DNA file in ApE and using the translation feature.
17 Cut and paste this translated sequence into the word document and label it as such.
18 Confirm that there are no amino acids in front of the nanobody starting signature, e.g., only QVQLVES, QLQLVES, QVKLEES, QVQVVES, DVQLVES, EVQVVES, or EVQLVES, and the ending signature is only TVSS or TVSA.
  If this is inaccurate, trim the nucleotide sequence, include the trimmed version in the word document as well and annotate it as a trimmed sequence. Use the ApE translate tool again to check it.
19 Depending on where the restriction site occurs near the end of the nanobody, modify the sequence further. The restriction site will be found in one of the following three scenarios:
  a. If the restriction site occurs within the TVS delete any nucleotide sequence that occurs after the cut site.
    For example, if using restriction enzyme BstEII, which cuts G/GTNACC, keep only the first G in the word document and remove everything past the cut site.
  b. If the restriction site occurs a few bases before the TVS is encoded in the vector, trim off the entire TVSS or TVSA encoding nucleotide sequence off the new nanobody in the word document as the TVS site is built into the plasmid.
    In this scenario nucleotides that cooperate with the restriction site of the vector may need to be inserted after trimming the TVSS or TVSA. For example, in this protocol the unique restriction site is BstEII that cuts G/GTNACC and this occurs before the TVSS, which is built into the vector. To maintain the frame and the cut site, two bases are added at the end of the new nanobody sequence, and a CA was put at the end of the trimmed sequence. The inserted bases should not translate to amino acids that can interfere with protein synthesis.
    Here is an example of the process: If the nanobody ends with an LVTVSA, represented by nucleotide sequence CTGGTCACTGTCTCTGCG and the restriction site of the vector is G/GTCACCGTCTCCTCA where the amino acids built into the vector are VTVSS. Trim off the TVSA leaving behind only CTGGTC from the original sequence. If a vector restriction site is added to the end without inserting bases, it will result in a frameshift where CTGGTCG/GTCACCGTCTCCTCA translates to LVGHRLL. To amend this, add two bases before the restriction site, in this case a CA was added to make CTGGTC **CA** G/GTCACCGTCTCCTCA translating to LVQVTVSS in the vector. This allows for preservation of the frame of the nanobody end signature translation as well as the restriction site by incorporating relatively innocuous amino acids.
  c. If the restriction site is found after the TVSS signature in the vector, copy and paste all the bases that are present between the end of the TVSS sequence in the vector up to the cut site of the restriction enzyme in the ApE file to the end of the sequence in the word document.
20 Once the nanobody nucleotide sequence has been trimmed/modified in the word document, go back to the ApE file and highlight and copy all the bases that are to the right of the first restriction site that was identified in steps 7 to 8 of this protocol up to (and including) the bases encoding for the MA that comes directly before the nanobody sequence in the vector. Paste these directly in front of the trimmed nucleotide sequence in the word document.
  The nucleotide sequence in the word document should now have the entire insertion sequence from the right of the first restriction site to the left of the second restriction site. Note on the word document that this is the “vector insertion sequence.”
21 Highlight and copy the entire vector insertion sequence from the word document, then go to the whole vector sequence in ApE and highlight everything between the two restriction sites, delete it, and replace it by pasting the insertion sequence. Save this ApE file as the new nanobody expression vector. This will be used as a reference later to confirm the cloning reaction is correct after sequencing.
22 In the new ApE file, use a cursor to highlight from the ATG that starts the PelB sequence all the way to ∼50 nt beyond the second restriction site. Hit “control T” or “command T” to use the translation tool to confirm that the new nanobody translates in frame. Look for the starting nanobody signature to be present in the translation as well as the TVSS or TVSA. Beyond the TVSS or TVSA, a stop codon in the translation should be visible.
  Any tags to be fused to the nanobody (e.g., epitope, affinity, or fluorescent tags) should be translated between the TVSS or TVSA and the stop codon. In many cases this will be a His_6_‐tag, but modifications will vary depending on the expression construct. Basic Protocol 11 operates under the assumption that a His_6_‐tagged nanobody is being used.
23 If everything looks correct in the translation, use the ApE sequence to create a geneblock for purchasing. Use the cursor to highlight from 20 to 30 nt before the first restriction enzyme site to 20 to 30 nt after the second restriction enzyme site and using copy/paste to insert this into the word document. Indicate in the document that this is the geneblock that will be ordered from the vendor.
  This is a block that has overhangs of 20 to 30 bp on each side of the restriction sites. These numbers are ideal for Gibson Assembly; however, the block needs to be a minimum of 300 bp to be produced synthetically by Twist and other vendors. If the nanobody sequence is shorter than prototypical nanobodies, extend each end of the block evenly creating longer overhangs of up 50 bases on each side.
  Highlight the expression vector overhangs that facilitate cloning of the geneblock in the word document. This is useful for making future nanobody geneblock constructs. A brief description of the steps taken to create a new expression vector with each new nanobody sequence in the word document will serve as a guide.
  We order our geneblocks from Twist Bioscience (see Internet Resources), but any vendor that provides this service can be used. For ordering via Twist, we order Gene Fragments without adapters, dried down in 2‐ml tubes, capped at 1 µg per tube.

#### Linearizing pHEN (or other expression construct) for cloning

24 Set up the following double digestion with BstEII‐HF and PstI in a 1.5‐ml tube on ice:
  10 µl of 10× CutSmart buffer
  2.5 µg pHEN DNA
  2.5 µl BstEII‐HF
  2.5 µl PstI
  84 µl nuclease‐free water.
    Restriction enzymes should always be kept on ice when setting up a digest. The reagents discussed here are specific to this protocol, but a different vector and/or different restriction enzymes can be used. Regardless, the procedure should remain the same.
25 Incubate overnight in a 37°C heat block or water bath.
26 Run the digest on a 1% agarose gel until bands are clearly separated as described in Basic Protocol 5: Gel purification, using loading dye and DNA ladder.
27 Cut out the linearized plasmid and gel purify using the Zymoclean Gel DNA Recovery kit as described in Basic Protocol 5.
28 Quantify linearized plasmid DNA using a Nanodrop. Store samples at –20°C.

#### Geneblock resuspension for cloning

29 Spin down tubes containing the geneblocks from the vendor 30 s at 2000 × *g*, room temperature.
  If ordered from Twist, geneblocks typically come as 1000 ng dried, but occasionally the yield is lower. Always confirm and check the tube label before preparing.
30 Add 100 µl of nuclease‐free water to the tube if starting with 1000 ng of geneblock. The final working concentration should be 10 ng/µl. If the yield of the dried geneblock is lower, adjust water accordingly.
31 Briefly vortex (∼5 s) and spin down tubes 30 s at 2000 × *g*, room temperature.
32 Incubate at 50°C for 20 min.
33 Briefly vortex and spin down 30 s at 2000 × *g*, room temperature.
34 Note the final concentration of the geneblock on the tube and use immediately or store at –20°C.

#### Gibson Assembly: Cloning the nanobody sequence into an expression vector

35 Thaw 2× Gibson Assembly master mix on ice and spin down the tube 30 s at 2000 × *g*, room temperature.
36 Set up the following reaction in a 1.5‐ml tube:
  10 µl of 2× Gibson Assembly master mix
  20 ng Geneblock
  7 to 10 ng of linearized pHEN (or other expression vector)
  Nuclease‐free water to bring the total reaction volume to 20 µl.
37 Incubate reaction at 50°C for 1 hr.
38 Store samples on ice or at –20°C until ready for transformation.

#### Transformation of cloning reaction into DH5α E. coli

39 Prewarm the SOC medium and the LB agar plates with carbenicillin to 37°C.
40 Thaw 250 µl of chemically competent DH5α cells on ice.
41 Divide the cells equally into two 1.5‐ml tubes (one for a negative control and one for the cloning reaction).
  Ensure the competent cells come from the same pool to control for contamination.
42 Add 3 µl of Gibson Assembly reaction to one tube of competent cells.
43 Incubate on ice for 30 min, flicking the tube occasionally (do not vortex).
44 Heat shock by incubating both tubes in a 42°C water bath for 30 s.
  Heat shock is a method of transformation in which a rapid change in temperature (from low to high) creates pores in the bacterial membrane, allowing plasmid DNA to enter the cell.
45 Immediately incubate on ice for 2 min.
46 Add 500 µl of pre‐warmed SOC medium to each tube and transfer the mix to sterile 5‐ml tubes.
47 Incubate tubes, shaking (∼200 rpm), at 37°C for 1 hr.
48 Plate 125 µl of negative control cells on a pre‐warmed LB agar plate with carbenicillin.
49 Plate 125 µl of cloning transformation on each of five pre‐warmed LB agar plates with carbenicillin.
50 Incubate plates at 37°C overnight.
  The colony density that is seen on the plates the following morning can be variable. A lawn of colonies would not be expected but anticipate at least 10 to 100 colonies per plate, sometimes more. It only takes one colony with the right sequence; do not be discouraged if there are only a few colonies on the plates.
51 Pick 3 to 6 of the resulting colonies and grow them overnight in a 2.5 ml culture of LB growth medium with carbenicillin, shaking (∼200 rpm), at 37°C.
52 Make glycerol stocks and perform plasmid mini preps as described in Basic Protocol 9, but do not incorporate tetracycline into the growth medium as these cells should only harbor resistance to carbenicillin.
  a. Have a designated –20°C freezer box to keep nanobody expression plasmids.
  b. Remove mini preps with sequences that are not a match.

#### Whole plasmid sequencing of the prepped DNA from the cloning reaction and interpretation

53 Send three of the mini‐prepped plasmids to Plasmidsaurus (see Internet Resources) or another vendor that performs whole‐plasmid sequencing for whole plasmid sequencing per the vendor instructions. After sequencing results are returned, open the FASTA files in UGENE as follows.
54 Click on “File” and select “Open” from the drop‐down menu.
55 Select multiple sequence files as well as the ApE reference sequence file for the nanobody of interest and click “Open”.
56 The “Multiple Sequence Reading Mode” window will pop up.
57 Click the “Join sequences into alignment” radio button, then click “OK” to see the multiple alignment of the sequences.
  Place the nanobody reference sequence at the top. Shift the position of any of the sequences up or down by clicking on the name to select it, then dragging it wherever needed. In our case the returned sequences and the reference sequence are offset and need to be adjusted to compare the reference to the returned sequence. If the returned sequence is already aligned with the reference sequence, skip steps 58 to 61 and go to step 62.
58 On the returned sequence, highlight the first 10 to 20 bases and use the search function to locate them in the reference sequence. Starting with the first base, shift the returned sequences to the right until the first 10 to 20 bases are in‐line with the reference sequence.
59 Scroll right to the very end. The reference sequence should now have an overhang.
60 Copy the overhang sequence and scroll back to the beginning of the alignment.
61 Paste the overhang below the rest of the sequences. This overhang should be a match for the starting section of the reference sequence.
62 Slowly scroll to the right and check that the returned sequences match the reference sequence exactly.
  Check every nucleotide of the sequence and confirm that there are no point mutations that could ultimately prevent expression of the nanobody. Look at the amino acid nanobody sequence alignment to confirm it is an exact match as well.
63 If one or more of the returned sequences are a match, note that in the glycerol stock list. If there are multiple matches, denote which one(s) will be transformed into WK6 *E. coli* to produce the nanobody.

## Basic Protocol 11 NANOBODY OVER‐EXPRESSION AND PURIFICATION

**Figure 10:**
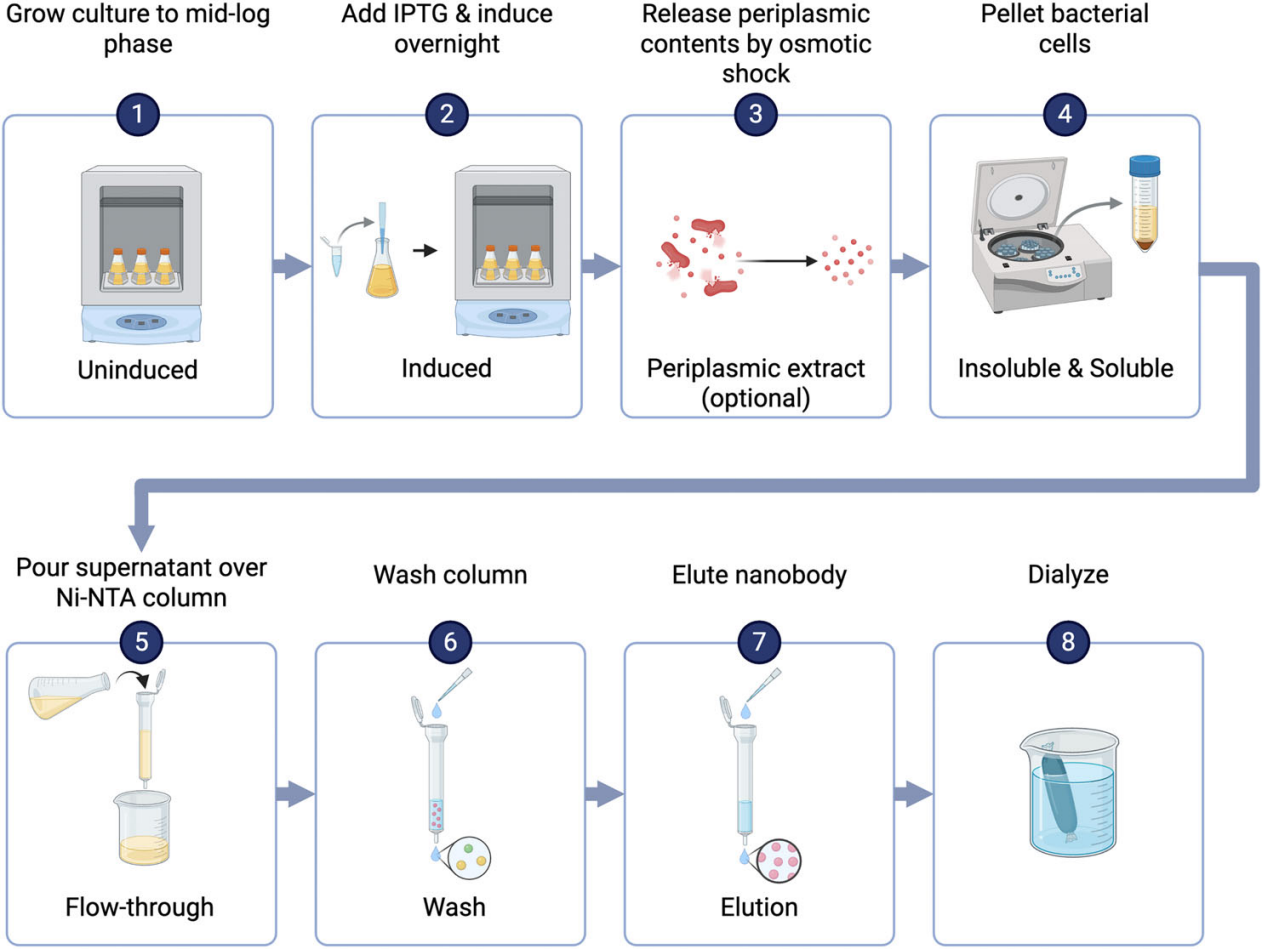
A graphical overview depicting key stages in the purification of nanobodies (Basic Protocol 11). *E. coli* cultures are grown and induced for expression of nanobodies, which are subsequently purified for downstream applications.

In Basic Protocol 11, the candidate nanobody‐encoding expression plasmids created in Basic Protocol 10 will be transformed into WK6 *E. coli* and nanobody expression will be induced, followed by subsequent nanobody purification (Fig. 10). pHEN directs nanobody accumulation to the periplasm via a PelB leader tag. The periplasm is an oxidizing compartment in the *E. coli* that is permissive for soluble folding and precise disulfide bond pairing/formation (Glockshuber et al., 1992). Nanobody accumulation in the periplasm also simplifies purification, as nanobodies can be released from the periplasm by an osmotic shock treatment. This approach yields cleaner preparations than traditional *E. coli* lysis methods (Karyolaimos & de Gier, 2021). Isolation of His_6_‐tagged nanobodies from *E. coli* lysates is achieved through affinity purification over Ni‐NTA agarose beads. The purified nanobody is eluted from the beads using high concentrations of imidazole and subsequently dialyzed against an imidazole‐free buffer to reduce imidazole concentrations <10 µM. Finally, analysis of fractions collected during the purification process will provide information about the quality of the nanobody preparation and inform potential protocol optimizations. At the end of this protocol series, ready‐to‐use purified nanobodies will be available in mg quantities that will allow for several downstream applications (e.g., validation, tagging, functional assays).

### Materials

LB growth medium with carbenicillin and streptomycin (see recipe)
SOC medium (see recipe)
*E. coli* WK6 competent cells (ATCC, cat. no. 47078)
Nanobody expression construct that was cloned in Basic Protocol 10
Terrific broth (TB) (see recipe)
Carbenicillin 1000× stock solution (see recipe)
Streptomycin 1000× stock solution (see recipe)
1 M IPTG (see recipe)
1× TES (see recipe)
0.25× TES (see recipe)
Dialysis buffer (see recipe)
PureCube Ni‐NTA agarose (VWR, cat. no. 77393‐438)
Nanobody wash buffer (see recipe)
Nanobody elution buffer (see recipe)
Laemmli buffer (see recipe)
1× PBS (see recipe)
15% SDS‐PAGE acrylamide gel (see recipe)
Precision Plus Protein Kaleidoscope prestained protein standard (Bio‐Rad, cat. no. 1610375)
1× SDS‐PAGE running buffer
dH_2_O
Coomassie staining solution (see recipe)
Coomassie destaining solution (see recipe)
Water Bath
Ice container with ice
10‐, 20‐, 200‐, and 1000‐µl filtered pipette tips and single channel pipettes
1.5‐ml microcentrifuge tubes
5‐ml microtubes (Eppendorf, cat. no. 14‐282‐300)
Shaking incubator that can accommodate 2‐L flasks
2‐L baffled flasks (Pyrex, cat. no. 4446‐2L)
Cuvette spectrophotometer at 600 nm
Disposable cuvettes, 1.5‐ml (Fisherbrand, cat. no. 14‐955‐127)
Refrigerated centrifuge capable of 7000 × *g* with rotors for 250‐ or 500‐ml bottles, 4°C
250‐ or 500‐ml centrifuge bottles compatible with centrifuge rotors
Lab balance
Benchtop rocker, to be used at 4°C
Cold room at 4°C
5‐L beaker
Econo‐Pac chromatography columns (Bio Rad Laboratories, cat. no. 7321010)
5‐, 10‐, and 25‐ml serological pipettes
Pipette controller or pipette gun
Dialysis tubing closures: Spectra/Por standard type, 55‐mm (Spectrum, cat. no. 132737)
Stir plate
Magnetic stir bars
Criterion Cell (Bio‐Rad, cat. no. 1656001)
Beadsmith case (Amazon, cat. no. B08633RNLW)
Orbital rocker (Genesee Scientific, cat. no. 31‐438)
Flatbed color scanner (any compatible model will suffice)

#### Nanobody expression and purification

##### Day 1: Transformation of pHEN into WK6 E. coli

1 Prewarm LB medium with carbenicillin and streptomycin to 37°C; prewarm SOC medium to 37°C.
2 Thaw 200 µl of WK6 *E. coli* on ice. Divide the cells equally into two 1.5‐ml tubes by pipetting, one for a negative control and one for the cloning reaction.
3 Add 50 ng of sequence‐verified nanobody expression construct to one tube of WK6 *E. coli* (the other tube is the negative control).
4 Incubate on ice for 30 min, flicking the tube occasionally (do not vortex).
5 Heat shock the cells by incubating both tubes at 42°C for 30 s.
6 Immediately incubate on ice for 2 min.
7 Add 500 µl of pre‐warmed SOC medium to each tube and transfer the mix to sterile 5‐ml tubes.
8 Incubate the tubes, shaking (∼200 rpm), at 37°C for 1 hr.
9 Pipette 200 µl of negative control into 10 ml of pre‐warmed LB medium with carbenicillin and streptomycin.
10 Pipette the entire transformation reaction into a minimum of 10 ml of pre‐warmed LB containing carbenicillin and streptomycin per L of nanobody to be prepped (e.g., to prepare 3 L of nanobody, add the entire transformation to 30 ml of LB with antibiotics).
  Streptomycin is being used to select for only WK6 E. coli cells, which are resistant to streptomycin.
11 Incubate cultures with shaking at 37°C overnight.
  Prepare an appropriate amount of TB, usually between 1 to 4 L per nanobody to be used in the morning on Day 2. Prepare the broth in 2‐L baffled flasks using 1 L per flask.

###### Day 2: Growth of TB culture and induction

12 Remove 1 ml of sterile TB to use as a blank for the spectrophotometer.
13 Add 1 ml each of 1000× carbenicillin and streptomycin to TB and inoculate 1:100 with overnight culture.
14 Incubate with shaking (∼150 rpm) at 37°C until the culture reaches an OD600 between 0.6 to 0.8.
  This takes 1.5 to 3 hr. Set the OD600 to zero with the TB blank before measuring the OD600 of the growing culture using disposable cuvettes and a spectrophotometer. Measure the culture OD600 after 1 hr of incubation, then every 30 min after that until the desired OD600 has been reached.
15 Once the culture reaches the appropriate cell density, remove 1 ml to keep as an “uninduced” fraction. Store this and all subsequent fractions at 4°C.
  Throughout this protocol, fractions will be removed for assessment via SDS‐PAGE gel to confirm protein expression, lysis, and proper purification.
16 Induce protein expression by adding 1 M IPTG to a final concentration of 1 mM.
17 Incubate cultures with shaking at 37°C.
  Optimal induction conditions must be empirically determined for each nanobody. See Troubleshooting section below.

###### Day 3: Cell collection and lysis

18 Pre‐cool a centrifuge and a rotor that accommodates 500‐ml centrifuge bottles to 4°C.
  Use a refrigerated centrifuge that has rotors for 250‐ and 500‐ml centrifuge bottles. Use 500‐ml bottles, but for smaller culture volumes, use 250‐ml bottles.
19 Collect the induced TB culture and remove 1 ml to keep as an “induced” fraction.
20 Split ∼1 L of induced TB equally between four 500‐ml centrifuge bottles.
  Use a lab balance to confirm each bottle weighs the same amount.
21 Spin down the TB culture 15 min at 4500 × *g*, 4°C, in the pre‐cooled centrifuge.
  Multiple spins using the same four centrifuge bottles will be required due to the large volume of induced culture. Always ensuring that the bottles are balanced, repeat this procedure until all the culture has been pelleted. Do not fill the bottles more than ¾ full for each spin.
22 Discard supernatant.
23 Resuspend pellets in 40 to 100 ml of 1× TES buffer per L of culture.
  Keep the resuspension in the same centrifuge bottle the culture was pelleted in. 40 ml is a starting suggestion, but in some cases, purification will benefit from a larger volume.
24 Once the pellets are completely resuspended, incubate with rocking at 4°C for 1 hr.
25 Add two volumes of 0.25× TES buffer relative to the initial resuspension volume.
  For example, if the pellet was resuspended in 40 ml of 1× TES, add 80 ml of 0.25× TES. Maintain this ratio. Balance the centrifuge bottles at this point as was done for the induced spins.
26 Incubate with rocking at 4°C overnight in a cold room.

###### Day 4: Protein isolation, purification, and dialysis

27 Prepare two 5‐L beakers each with 4 L of dialysis buffer (see recipe) and store at 4°C until ready for use.
  IMPORTANT: Before beginning dialysis, determine what the final use of the protein will be and use an appropriate dialysis buffer that is compatible with desired downstream applications.
28 Pre‐cool a centrifuge and a rotor that accommodates 500‐ml centrifuge bottles to 4°C in preparation of pelleting the culture that incubated at 4°C overnight.
  At this point, the periplasmic contents should be liberated due to the action of the osmotic shock buffer, meaning that the expressed nanobodies are released into the supernatant.
29 Optionally, take 100 µl as a “periplasmic extract” fraction from the overnight osmotic shock culture.
  The soluble and insoluble fractions will be separated in the next steps of this protocol, and a sample of each fraction will be collected. The “periplasmic extract” is not required for analysis but can be a representation of the total nanobody abundance in the culture before separation.
30 Spin down the osmotic shock culture (still in the two 500‐ml centrifuge bottles) for 15 min at 6200 × *g*, 4°C.
  The goal after this step is to produce a relatively low viscosity, transparent supernatant.
31 Chill a bottle on ice that can hold the total volume of supernatant.
32 While the cultures are pelleting, prepare the chromatography columns. Perform all steps in a 4°C cold room.
33 Break off the tip of the chromatography column and place it in a rack that allows a 5‐L beaker to be placed under it.
34 Gently shake the Ni‐NTA agarose beads until they are well‐mixed.
35 Using a serological pipette, add 2.5 ml of bead slurry into the column.
36 Equilibrate the beads by washing with ∼50 ml of 0.25× TES.
  The beads are stored in 30% ethanol; this needs to be removed by washing to create an even interface between the fluid and the beads.
37 Cap the column with the yellow tip cap before all liquid has flowed through. Keep the beads submerged to prevent the beads and the sample from drying, which would negatively impact the elution as well as damage the beads, preventing them from being able to be recharged for future use.
38 Collect cultures from the centrifuge and transfer the supernatant to the pre‐chilled bottle. Remove 100 µl as a “soluble” fraction.
39 Before discarding the pellets, remove a small amount as an “insoluble” fraction.
  Do this by poking the pellet with a toothpick or pipette tip and wiping the cells on the inside of a 1.5‐ml tube. Only a very small amount is needed.
40 Pour the supernatant over the prepared column. Time will vary depending on viscosity and total volume of the supernatant. Do this gently to avoid disturbing the bead surface.
41 Take 100 µl of the flow‐through as a “flow‐through” fraction. Take this fraction after the first 25 ml have passed through the column.
42 After all the supernatant has passed through the column, wash the column with 100 ml of nanobody wash buffer.
  The low imidazole concentration of the wash buffer will remove any unwanted proteins stuck to the beads, either physically or via ionic interactions.
43 Save 100 µl of the wash as a “wash” fraction. Take this fraction after 50 ml of wash buffer have passed through the column. Enough wash buffer needs to pass over the beads to get a representative sample of the wash.
44 Allow all the wash buffer to flow out of the column, then cap the tip.
45 Elute nanobody by adding 1 to 5 ml elution buffer to the column. Agitate the beads by pipetting them up and down in elution buffer with a 5‐ml serological pipette.
  A minimum elution volume is needed where the beads will be completely submerged for the incubation. Lower volumes can concentrate the sample but can also over‐concentrate it, causing it to crash out of solution. Start with a 2.5 ml elution buffer addition, incubation, and collection followed by a second 2.5 ml elution buffer addition, incubation, and collection on the same beads the first time a new nanobody is being purified. We then use that to assess optimal elution incubation volumes and timing for future purifications of the same nanobody.
46 Incubate in elution buffer for 15 min, then collect eluate in a 5‐ml tube.
47 Remove 30 µl as an “elution” fraction.
48 To save the beads for recharging, cap the tip of the column, add 2 to 3 ml of water or 30% ethanol, or enough to submerge the beads to prevent them from drying out, secure the lid, and store at 4°C.
  By the end of this procedure, the following fractions should have been obtained: (1) uninduced, (2) induced, (3) total lysate (optional), (4) soluble, (5) insoluble, (6) flowthrough, (7) wash, and (8) elution.

###### Setting up dialysis

49 Cut a piece of snakeskin dialysis tubing ∼75 mm in length. This is dependent on the volume of solution to be dialyzed.
  It is important to regard the age of this tubing; once it has been opened it can become more brittle over time, increasing risk of tears and pinholes. Always store in a cool, dry place and inspect the integrity of the tubing before use.
50 Thoroughly wet the dialysis tubing in the dialysis buffer.
  Snakeskin dialysis tubing can be prepared dry. This will make it clear when checking for leaks, but it increases the chances of tearing the tubing during prep. The pre‐wetting step minimizes the potential for tears in the tubing.
51 Flatten using your fingers and use a clip to close one end of the tubing, creating a small pouch.
52 Carefully hold the pouch and pipette the nanobody elution inside.
  Do this gently and slowly to avoid introducing bubbles.
53 Leaving a small overhang of dialysis tubing, clip the open end closed and confirm both ends are secure.
  You will need to be able to grip the dialysis tubing to remove the clip later. Confirm the overhang is extended enough to grip without using an excessive amount of tubing.
54 Float the pouch in 4 L of dialysis buffer, slowly stirring at room temperature for 3 to 4 hr, then transfer the entire pouch assembly into fresh 4 L of dialysis buffer and dialyze at 4°C overnight.
  This should have very light stirring. If a vortex forms, it is too fast and can rip the dialysis tubing. If the pouch sinks, retrieve it, confirm that the tubing is intact, and resume stirring. Dialyzing nanobodies at 4°C in the presence of high imidazole concentrations can cause them to precipitate. To prevent this, dialyze at room temperature for a few hours to reduce the imidazole concentration, then dialyze overnight at 4°C.
55 Recover the dialyzed nanobody by carefully opening one end of the pouch and pipetting out the solution into a sterile tube.
56 Dialyzed nanobodies can be used immediately or stored at –80°C with the addition of 15% to 20% glycerol as a cryoprotectant. Nanobodies will remain intact without the addition of glycerol, but this runs the risk of cleaving detection tags.

#### Validation of purified nanobody using SDS‐PAGE and Coomassie staining

Just as protein quality was assessed in Basic Protocol 2 using SDS‐PAGE and Coomassie staining, this method can also be applied here, with minor modifications, to evaluate the efficiency of the nanobody purification.

**Figure 11:**
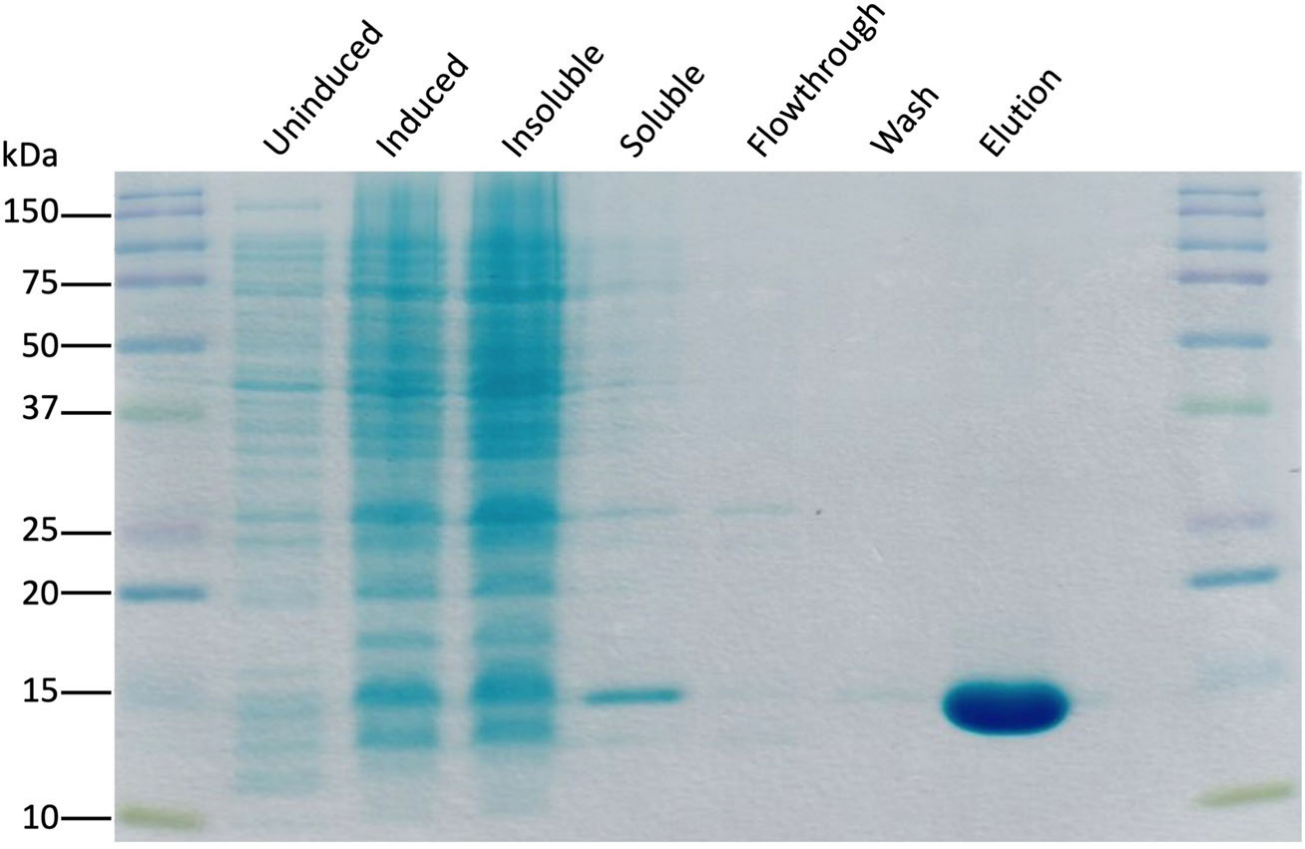
Coomassie‐stained SDS‐PAGE gel showing the fractions taken during the nanobody purification process. The ∼15 kDa nanobody band appears in the induced fraction and is still present in the soluble fraction. Very little protein is seen in the flowthrough or wash fractions. The elution fraction shows a strong single band at the expected size. Created in BioRender. K. Mitchell (2026).

57 To prepare fractions for loading onto an SDS‐PAGE gel, add 6× Laemmli buffer to a final concentration of 1× in all liquid samples. All solid samples should be resuspended in 1× PBS (see recipe) before adding 6× Laemmli buffer.
58 Boil sample for 5 min, then vortex and quickly spin down.
59 Load 20 µl of each sample into one lane of a 15% SDS‐PAGE acrylamide gel and load 10 µl of Precision Plus Protein Kaleidoscope prestained protein standard into another well. Fill any empty wells with 20 µl of 1× Laemmli buffer.
  Samples should be loaded in the following order: uninduced, induced, total lysate (if taken), insoluble, soluble, flowthrough, wash, and elution.
60 Run the 15% SDS‐PAGE acrylamide gel in 1× SDS‐PAGE running buffer for 65 min at 150 V in a Criterion Cell.
61 Remove the SDS‐PAGE acrylamide gel from its cassette and gently rinse with deionized water.
62 Submerge the gel in Coomassie staining solution and gently rock overnight at room temperature.
  Any container that is approved for gel staining will work, but Beadsmith boxes are preferred as they seem to provide the best seal, minimizing evaporation of reagents.
63 Remove Coomassie staining solution and rock the gel in Coomassie destaining solution, replacing the destaining solution every hour, for ∼4 hr at room temperature or until clear resolution of protein bands can be seen.
64 Scan the gel on a flatbed scanner and evaluate the efficiency of the purification.
  The nanobody band (∼15 kDa) should appear in the induced fraction and persist in the total lysate, insoluble, and soluble fractions. Ideally, there should be very little to no nanobody in the flowthrough and wash fractions, while the highest concentration of nanobody should be in the elution fraction, which should also be relatively free of other proteins (Fig. 11).

## REAGENTS AND SOLUTIONS

### 2xYT growth medium

100 ml dH_2_O
3.1 g Difco 2x yeast extract tryptone (2xYT) dehydrated growth medium (Fisher, cat. no. DF0440‐17)
Mix until dissolved
Autoclave at 121°C for 30 min (liquid cycle)
Allow to cool to ≤55°C
Add 100 µl each of antibiotic 1000× stock solution, if required
If preparing as a stock, store up to 3 months at 4°C in 50‐ to 100‐ml aliquots

### 2xYT/0.1% glucose growth medium

100 ml dH_2_O
3.1 g Difco 2x yeast extract tryptone (2xYT) dehydrated growth medium (Fisher, cat. no. DF0440‐17)
Mix until dissolved
Autoclave at 121°C for 30 min (liquid cycle)
Allow to cool to ≤55°C
Add 500 µl of 20% (w/v) glucose solution (see recipe)
Add 100 µl each of antibiotic 1000× stock solution, if required

### 2xYT/2% glucose agar plates

900 ml dH_2_O
Add autoclave safe stir bar
31 g Difco 2x yeast extract tryptone (2xYT) dehydrated growth medium (Fisher, cat. no. DF0440‐17)
Mix until dissolved
Add 15 g Difco agar (BD, cat. no. 214010), stir to incorporate (will not dissolve), keep stir bar in flask
Autoclave at 121°C for 30 min (liquid cycle)
Allow to cool to ≤55°C
Add 100 ml of 20% (w/v) glucose solution (see recipe)
Add 1 ml each of antibiotic 1000× stock solution, if required
Stir to combine
Pour into 15‐ to 20‐mm glass or plastic Petri dishes, 15 to 20 ml per dish
Allow to solidify
Store upside down up to 2 months at 4°C
*1 L makes ∼50 plates*.

### 2xYT/2% glucose growth medium

90 ml dH_2_O
3.1 g Difco 2x yeast extract tryptone (2xYT) dehydrated growth medium (Fisher, cat. no. DF0440‐17)
Mix until dissolved
Autoclave at 121°C for 30 min (liquid cycle)
Allow to cool to ≤55°C
Add 10 ml of 20% (w/v) glucose solution (see recipe)
Add 100 µl each of antibiotic 1000× stock solution, if required
If preparing as a stock, store up to 3 months at 4°C in 50‐ to 100‐ml aliquots

### Agarose gel

To a 200‐ml Erlenmeyer flask, add 1 g or 1.8 g (for 1% or 1.8% gel, respectively) of Agarose Apex Quick Dissolve LE Agarose, Ultra‐Pure, 500 g/Unit (Genesee Scientific, cat. no. 20‐102QD) and 100 ml of 1× TAE buffer (see recipe). Wet a paper towel with deionized water and plug the flask with it, but not too tightly where a seal is created. Microwave the flask in 30‐s increments, pulling out the flask and swirling it to mix in between rounds, being sure to point the flask away from face and other people; molten agarose can cause severe burns and if the wet towel plug is too tight the pressure from the agarose boiling can expel it. Repeat the microwave step for three to four rounds or until the agarose is fully dissolved and clear as water. Cool the flask on the benchtop until it reaches 50° to 60°C or is not too hot to touch and is still liquid. Pipette in 5 µl of 1% ethidium bromide (Fisher, cat. no. BP1302‐10). Swirl the flask until the ethidium bromide distributes and is no longer visible. Pour gel into gel casting tray with proper combs already in place. Prepare fresh.

### APS solution, 10% (w/v)

0.3 g ammonium persulfate (APS) (Fisher, cat. no. AC401165000)
3 ml dH_2_O
Mix in a 15‐ml conical tube (Fisher, cat. no. 352097)
Store up to 1 week at 4°C

### BSA solution, 1% (w/v)

8 ml of 1× PBS or 8 ml of 1× PBST (0.1% Tween 20), depending on experimental requirements
2 ml of 5% (w/v) BSA solution (see recipe)
Store up to 1 week at 4°C

### BSA solution, 5% (w/v)

20 ml of 1× PBS (see recipe)
1 g bovine serum albumin (BSA) (5% w/v final; Fisher, cat. no. BP1605‐100)
Mix until dissolved
Aliquot and store up to 1 year at –20°C

### Carbenicillin stock solution, 1000×

10 ml dH_2_O
1 g carbenicillin disodium salt (100 mg/ml final; Fisher, cat. no. BP26485)
Mix until dissolved
Filter with a Steriflip 0.22‐µm PES filtration system (MilliporeSigma, cat. no. SCGP00525)
Aliquot and store up to 1 year at –20°C

### Coomassie destaining solution

400 ml dH_2_O
500 ml methanol (50% v/v final; Fisher, cat. no. A433P)
In a fume hood, add 100 ml glacial acetic acid (10% v/v final; Fisher, cat. no. A38‐212)
Store up to 6 months at room temperature

### Coomassie staining solution (250 ml)

0.25 g Coomassie Brilliant Blue G‐250 (0.1% w/v final; Fisher, cat. no. PI20279)
125 ml methanol (50% v/v final; Fisher, cat. no. A433P)
Stir with a magnetic stir plate for a few minutes
Add 100 ml deionized water
Add 25 ml glacial acetic acid (10% v/v final; Fisher, cat. no. A38‐212)
Fold a piece of filter paper into quadrants and open positioning 3 layers on one side and one on the other
Wet cone with deionized water and place into an appropriately sized funnel
Place funnel into 250‐ml bottle
Pour staining solution into cone and allow the drip through via gravity flow
Store up to 1 year at room temperature
  Staining solution can be reused; pigment will decrease over time.

### Dialysis buffer

3.5 L dH_2_O
24.23 g Tris base (50 mM final; Fisher, cat. no. BP152‐1)
35.06 g NaCl (150 mM final; Fisher, cat. no. BP358‐212)
Adjust to pH 7.5 with dilute HCl
Adjust volume to 4 L with deionized water
Store up to 1 week at 4°C

### Ethanol, 70% (v/v)

*For nucleic acid work, add the following to a 50‐ml conical tube*:
35 ml of 100% ethanol (Fisher, cat. no. 04‐355‐222)
15 ml UltraPure nuclease‐free water (Invitrogen, cat. no. 10977015)
Mix by inverting the tube
Store up to 6 months at room temperature
*For basic laboratory decontamination, add the following to a carboy that can hold at least 6 L*:
1 gallon (∼3.79 L) of 100% ethanol (Fisher, cat. no. 04‐355‐223)
1.62 L dH_2_O
Decant into spray bottles as needed
Store up to 6 months at room temperature

### Glucose solution, 20% (w/v)

100 ml dH_2_O
20 g dextrose (20% w/v final; Fisher, cat. no. D16‐1)
Stir until dissolved
Filter with a 0.22‐µm PES bottle‐top filter (Fisher, cat. no. FB12566510)
Store up to 2 months at room temperature

### Glucose solution, 50% (w/v)

100 ml dH_2_O
50 g dextrose (50% w/v final; Fisher, cat. no. D16‐1)
Stir until dissolved
Filter with a 0.22‐µm PES bottle‐top filter (Fisher, cat. no. FB12566510)
Store up to 2 months at room temperature

### Glycerol solution, 50% (v/v)

250 ml dH_2_O
250 ml glycerol (Fisher, cat. no. 77510‐264)
Mix to combine
Autoclave at 121°C for 30 min (liquid cycle)
Aliquot into 50‐ml conical tubes under sterile conditions
Store up to 1 year at room temperature

### Glycine solution, 0.2 M, pH 2.2

70 ml dH_2_O
1.5 g glycine (0.2 M final; Fisher, cat. no. BP3815)
Mix until fully dissolved
Adjust to pH 2.2 with 1 M HCl
Adjust volume to 100 ml with deionized water
Store up to 1 year at room temperature

### Hydrochloric acid, 2 M (dilute for pH adjustments)

80 ml dH_2_O
20 ml of 10 N HCl (Fisher, cat. no. SA49)
Store in appropriate corrosives storage for up to 1 year at room temperature
  Prepare in different ratios as needed. HCl is extremely corrosive; wear appropriate PPE, prepare in a fume hood, and always add acid to water.

### Hydrochloric acid, 1 M

In a glass graduated cylinder add:
90 ml dH_2_O
10 ml pf 10 N HCl solution (Fisher, cat. no. SA49)
Pour into a glass bottle for mixing and storage, no stir bar required
Rinse the glassware and outside of the stock bottle thoroughly before storage or glass wash
Store in appropriate corrosives storage for up to 1 year at room temperature
  HCl is extremely corrosive; wear appropriate PPE, prepare in a fume hood, and always add acid to water.

### Imidazole solution, 5 M

40 ml dH_2_O
17.02 g imidazole (Fisher, cat. no. MP21020331)
Stir to dissolve
Add deionized water to 50 ml
Filter with a Steriflip 0.22‐µm PES filtration system (MilliporeSigma, cat. no. SCGP00525)
Aliquot into 1 ml
Store up to 1 year at –20°C protected from light

### IPTG solution, 1 M

8 ml dH_2_O
2.38 g isopropyl‐β‐D‐thiogalactopyranoside (IPTG) (1 M final; Fisher, cat. no. BP1755‐10)
Mix until dissolved
Filter with a Steriflip 0.22‐µm PES filtration system (MilliporeSigma, cat. no. SCGP00525)
Aliquot and store up to 1 year at –20°C

### KCl, 1 M

70 ml dH_2_O
7.45 g potassium chloride (Fisher, cat. no. BP366)
Mix until fully dissolved
Adjust volume to 100 ml with deionized water
Store up to 1 year at room temperature

### Laemmli buffer

Place 6× Laemmli buffer (Fisher, cat. no. 04‐822‐261) in a 37°C incubator for ∼30 min prior to use
*For 2× Laemmli buffer*:
4 ml of 6× Laemmli buffer
8 ml dH_2_O
Dilute experimental samples 1:1 for a final concentration of 1×
  This 2× stock provides a Laemmli solution that is not prone to precipitation that can be stored long term at room temperature and used for multiple experiments. It has a disadvantage of doubling the sample loading volume and should be taken into consideration before adding to the sample as this could exceed the loading capacity of the SDS‐PAGE gel. If exceeding the gel loading capacity with the 2× buffer, then use the 6× to 1× dilution formula.
*For 1× Laemmli buffer (1× in‐sample dilution)*:

Divide the total volume of the sample by 5 to determine the volume of 6× Laemmli buffer needed. Add the calculated volume of pre‐warmed 6× Laemmli buffer directly to the sample. For example, if there is a 25 µl of sample to load, add 5 µl of 6× buffer (25 ÷ 5 = 5) giving a final total volume of 30 µl resulting in a 1× dilution that can be confirmed with C_1_V_1_ = C_2_V_2_. (6×)(5 µl) = (1×)(30 µl).

### LB agar plates

1 L dH_2_O
25 g LB powder, Miller (Fisher, cat. no. BP1426‐500)
Stir until fully dissolved
Add 15 g Difco agar (BD, cat. no. 214010), stir to incorporate (will not dissolve), keep stir bar in flask
Autoclave at 121°C for 30 min (liquid cycle)
Allow to cool to ≤55°C before adding any required antibiotics
Add 1 ml each of antibiotic 1000× stock solution, if required
Stir to combine
Pour or pipette 15 to 20 ml solution into Petri dishes
Allow to solidify
Store upside down up to 3 months at 4°C
  1 L makes ∼50 plates.

### LB growth medium

1 L dH_2_O
25 g LB powder, Miller (Fisher, cat. no. BP1426‐500)
Stir until fully dissolved
Autoclave at 121°C for 30 min (liquid cycle)
Allow to cool to ≤55°C before adding any required antibiotics
Add 1 ml each of antibiotic 1000× stock solution, if required
Store autoclaved medium up to 3 months at 4°C

### Milk in PBST, 4%

90 ml PBS (see recipe)
1 ml Tween 20 (1% v/v final; Thermo, cat. no. AAJ20605AP)
4 g dry milk powder (Dot Scientific, cat. no. DSM17200‐1000)
Stir to combine
Bring up to 100 ml with PBS
Store up to 1 week at 4°C

### Milk in TBST, 5%

Add to a 50‐ml conical tube:
2.5 g dry milk powder (Dot Scientific, cat. no. DSM17200‐1000)
Fill to 50 ml with 1× TBST (see recipe)
Close tube and shake until all the milk powder has dissolved
Store up to 1 week at 4°C

### NaCl solution, 5 M

750 ml dH_2_O
292.2 g sodium chloride (NaCl) (Fisher, cat. no. BP358‐212)
Stir until dissolved
Bring up to 1 L with deionized water
Autoclave at 121°C for 30 min (liquid cycle)
Store up to 1 year at room temperature

### Nanobody elution buffer

8.4 ml dH_2_O
500 µl of 1 M Tris solution, pH 7.5 (50 mM final; see recipe)
300 µl of 5 M NaCl solution (150 mM final; see recipe)
800 µl of 5 M imidazole solution (400 mM final; see recipe)
Prepare fresh, keep at 4°C

### Nanobody wash buffer

1 L of 0.25× TES buffer (see recipe)
2 ml of 5 M imidazole solution (10 mM final; see recipe)
Prepare fresh, keep at 4°C

### NEB 100 bp ladder working stock

100 bp ladder (New England Biolabs, cat. no. N3231S)
167 µl of 6× gel loading dye, purple, no SDS (packed with product) to the 100‐µl tube of 100 bp ladder
Add 733 µl dH_2_O to the mixture for a final volume of 1000 µl
Load 10 µl of stock per ladder well of agarose gel
Aliquot, store up to 1 year at –20°C

### PBS, 1×

100 ml of 10× phosphate‐buffered saline (PBS), pH 7.4 (Invitrogen, cat. no. 70011069)
900 ml dH_2_O
Filter with a 0.22‐µm PES bottle‐top filter (Fisher, cat. no. FB12566510)
Store up to 6 months at room temperature

### PBST (0.1% Tween 20), 1×

1 L of 1× PBS (see recipe)
1 ml Tween 20 (0.1% v/v final; Thermo, cat. no. AAJ20605AP)
Stir to combine
Store up to 6 months at room temperature

### PBST (1% Tween 20), 1×

990 ml of 1× PBS (see recipe)
10 ml Tween 20 (1% v/v final; Thermo, cat. no. AAJ20605AP)
Stir to combine
Store up to 6 months at room temperature

### PEG (20% w/v) in 2.5 M NaCl solution

200 ml dH_2_O
100 g polyethylene glycol 6000 (20% w/v final; MilliporeSigma, cat. no. 89510‐1KG‐F)
Stir until dissolved
Add 250 ml of 5 M NaCl solution (see recipe)
Adjust volume to 500 ml with deionized water
Filter using a 0.22‐µm PES bottle‐top filter unit (Fisher, cat. no. FB12566510)
Store up to 6 months at 4°C

### Ponceau S solution

400 ml dH_2_O
25 ml glacial acetic acid (5% v/v final; Fisher, cat. no. A38‐212)
0.5 g Ponceau S (0.1% w/v final; Fisher, cat. no. 161470250)
Stir until dissolved
Adjust volume to 500 ml using deionized water
Store up to 1 year at room temperature protected from light

### Primer dilutions

1 tube of dried‐down primer from IDT will become the concentrated stock. Briefly spin down the dry primer in a benchtop microcentrifuge to ensure contents reach the bottom of the tube. Add an appropriate amount of ultra‐pure water (Invitrogen, cat. no. 10977023) to the tube to create a 100 µM concentrated stock. Check the side of the tube to determine water to add. The formula for resuspension is (nmol × 10) = µl of water to add for a 100 µM stock. For example, if the tube says 23.8 nmol then 238 µl of water would be added. Vortex. Heat the resuspension at 50°C for 20 min with shaking. In a separate microcentrifuge tube, combine 90 µl of water with 10 µl of the concentrated primer stock. This will be the 10 µM working stock. Store both the concentrated stock and the working stock up to 1 year at –20°C.

### Proteinase K stock solution, 10 mg/ml

100 mg of proteinase K (Invitrogen, cat. no. 25530015)
10 ml dH_2_O (add to the bottle of proteinase K powder)
Recap and shake to mix
Aliquot and store up to 1 year at –20°C

### SDS‐PAGE acrylamide gel

Criterion midi cassette, 12‐well or 18‐well (Bio‐Rad, cat. nos. 3459901 and 3459902)
*For 12% resolving gel (25 ml makes 2 gels)*:
10.75 ml dH_2_O
7.5 ml of 40% acrylamide stock solution (acrylamide/bis‐acrylamide, 37.5:1, 40% solution; Fisher, cat. no. BP1410‐1)
6.25 ml of 1.5 M Tris solution, pH 8.8 (0.375 M final; see recipe)
50 µl of 10% SDS solution (0.1% w/v final; see Current Protocols, 2006)
250 µl of 10% APS solution (0.1% w/v final; see recipe)
10 µl TEMED (0.04% v/v final; Fisher, cat. no. BP150‐100)
Vortex to mix thoroughly
Pour into cassette, leaving ∼0.5 cm space below end of well comb for stacking gel
Gently fill rest of cassette with deionized water
After gel is polymerized, discard water
Carefully dry cassette with strip of filter paper; avoid touching the gel
*For 15% resolving gel (25 ml makes 2 gels)*:
8.88 ml dH_2_O
9.38 ml 40% acrylamide stock solution
6.25 ml of 1.5 M Tris solution, pH 8.8 (0.375 M final)
250 µl of 10% SDS solution (0.1% w/v final)
250 µl of 10% APS solution (0.1% w/v final)
10 µl TEMED (0.04% v/v final; Fisher, cat. no. BP150‐100)
Vortex to mix thoroughly
Pour into cassette, leaving ∼0.5 cm space below end of well comb for stacking gel
Gently fill rest of cassette with deionized water
After gel is polymerized, discard water
Carefully dry cassette with strip of filter paper; avoid touching the gel
*For 5% stacking gel (10 ml makes 2 gels)*:
7.3 ml dH_2_O
1.25 ml of 40% acrylamide solution (5% w/v final)
1.25 ml of 1 M Tris, pH 6.8 (0.125 M final)
200 µl of 10% SDS solution (0.1% w/v final)
200 µl of 10% APS solution (0.1% w/v final)
10 µl TEMED (0.04% v/v final; Fisher, cat. no. BP150‐100)
Vortex to mix thoroughly
Pour on top of resolving gel, filling to top
Add ladder comb, gently tapping cassette to remove any bubbles
After gel polymerizes, remove gently ladder comb only when cassette is submerged in 1× SDS‐PAGE running buffer
If not using immediately, wrap gel with wet paper towel, then plastic wrap, and store up to 1 week at 4°C
Inspect for desiccation, separation, or contamination before use

### SDS‐PAGE running buffer, 1×

100 ml of 10× SDS‐PAGE running buffer (see recipe)
900 ml dH_2_O
Store up to 1 year at room temperature
  Working concentrations of 1×: 0.1% SDS, 192 mM glycine, and 25 mM Tris.

### SDS‐PAGE running buffer, 10×

700 ml dH_2_O
10 g SDS micropellets (1% w/v final; Fisher, cat. no. BP8200500)
144 g glycine (1.92 M final; Fisher, cat. no. BP3815)
30.2 g Tris base (250 mM final; Fisher, cat. no. BP152‐1)
Stir until fully dissolved
Adjust volume to 1 L with deionized water
Store up to 1 year at room temperature

### SOC medium

800 ml dH_2_O
5 g Bacto yeast extract (0.5% w/v final; Fisher, cat. no. DF0127‐17‐9)
20 g tryptone (2% w/v final; Fisher, cat. no. BP1421‐500)
0.5 g NaCl (10 mM final; Fisher, cat. no. BP358‐212)
2.5 ml of 1 M KCl (2.5 mM final; Fisher, cat. no. BP366‐500)
Stir to dissolve components
Adjust pH to 7.0 using 1 M NaOH (Fisher, cat. no. BP359‐212)
Adjust volume to 972 ml using deionized water
Autoclave at 121°C for 30 min (liquid cycle)
Cool to room temperature and add:
20 ml of sterile 1 M MgCl_2_ (20 mM final; MilliporeSigma, cat. no. M8266‐100G)
8 ml of 50% (w/v) glucose solution (0.4% w/v final; see recipe)
Mix until combined
Aliquot into 50‐ml conical tubes and store up to 1 year at –20°C

### Streptomycin stock solution, 1000×

10 ml dH_2_O
1 g streptomycin sulfate salt (100 mg/ml final; MilliporeSigma, cat. no. S9137‐25G)
Mix until dissolved
Filter with a Steriflip 0.22‐µm PES filtration system (MilliporeSigma, cat. no. SCGP00525)
Aliquot and store up to 1 year at –20°C

### TAE buffer, 1×

20 ml of 50× TAE buffer (see recipe)
980 ml dH_2_O
Store up to 1 year at room temperature
  Working concentrations of 1×: 40 mM Tris, 1 mM EDTA, and 20 mM acetic acid.

### TAE buffer, 50×

600 ml dH_2_O
242 g Tris base (2 M final; Fisher, cat. no. BP152‐1)
100 ml of 0.5 M EDTA, pH 8.0 (0.05 M final; Fisher, cat. no. BP24821)
57.1 ml glacial acetic acid (1 M final; Fisher, cat. no. A38‐212)
Stir until fully dissolved
Adjust volume to 1 L with deionized water
Filter with a 0.22‐µm PES bottle‐top filter unit (Fisher, cat. no. FB12566510)
Store up to 1 year at 4°C

### Tail digestion buffer (TDB)

To a 50‐ml conical tube, add:
125 µl of 20% (v/v) Triton (Fisher, cat. no. A16046AE)
250 µl of 1 M Tris, pH 9.0 (see recipe)
1250 µl of 1 M KCl (see recipe)
23.375 ml dH_2_O
Filter with a Steriflip 0.22‐µm PES filtration system (MilliporeSigma, cat. No. SCGP00525)
  This portion of the buffer can be stored long term at room temperature but is prone to contamination.
Addition of proteinase K to TDB:
10 ml TDB
25 µl of 10 mg/ml proteinase K (0.25 mg/ml final; see recipe)
Prepare fresh

### TBST, 1×

100 ml of 10× TBST (see recipe)
900 ml dH_2_O
Store up to 1 year at 4°C
  Working concentrations of 1×: 137 mM NaCl, 20 mM Tris, and 0.01% Tween 20.

### TBST, 10×

800 ml dH_2_O
80 g NaCl (1.37 M final; Fisher, cat. no. BP358‐212)
24.2 g Tris base (200 mM final; Fisher, cat. no. BP152‐1)
Adjust to pH 7.6 with 1 M HCl
Add 1 ml Tween 20 (0.1% final; Thermo, cat. no. AAJ20605AP)
Store up to 1 year at 4°C

### TB growth medium

900 ml dH_2_O
47 g Terrific broth (TB) powder (Invitrogen, cat. No 22711022)
Stir until fully dissolved
Add 8 ml of 50% (v/v) glycerol solution (see recipe)
Adjust volume to 1 L
Autoclave at 121°C for 30 min (liquid cycle)
Allow to cool to ≤55°C before adding any required antibiotics
Add 1 ml each of antibiotic 1000× stock solution, if required
If preparing as a stock, store up to 3 months at 4°C in 50‐ to 100‐ml aliquots

### TB/10 mM IPTG growth medium

90 ml dH_2_O
4.7 g Terrific broth (TB) powder (Invitrogen, cat. No 22711022)
Stir until fully dissolved
Add 800 µl of 50% (v/v) glycerol solution (see recipe)
Adjust volume to 100 ml
Autoclave at 121°C for 30 min (liquid cycle)
Allow to cool to ≤55°C before adding required antibiotics
Add 100 µl each of antibiotic 1000× stock solution, if required
Add 1 ml of 1 M IPTG solution (see recipe)
Prepare fresh before each use

### TB/2% glucose growth medium

800 ml dH_2_O
47 g Terrific broth (TB) powder (Invitrogen, cat. No 22711022)
Stir until fully dissolved
Add 8 ml of 50% (v/v) glycerol solution (see recipe)
Adjust volume to 900 ml
Autoclave at 121°C for 30 min (liquid cycle)
Allow to cool to ≤55°C
Add 100 ml of 20% (w/v) glucose solution (see recipe)
Add 1 ml each of antibiotic 1000× stock solution, if required
Stir to combine
If preparing as a stock, store up to 3 months at 4°C in 50‐ to 100‐ml aliquots

### TES buffer, 0.25×

250 ml of 1× TES buffer (see recipe)
750 ml dH_2_O
Store up to 1 year at 4°C
  Working concentrations of 0.25×: 125 mM sucrose, 50 mM Tris, and 0.1625 mM EDTA.

### TES buffer, 1×

700 ml dH_2_O
171.2 g sucrose (0.5 M final; Fisher, cat. no. S5‐3)
200 ml of 1 M Tris solution, pH 8.0 (0.2 M final, see recipe)
1.3 ml of 0.5 M EDTA, pH 8.0 (0.65 mM final; Fisher, cat. no. BP24821)
Adjust volume to 1 L with deionized water
Store up to 1 year at 4°C

### Tetracycline stock solution, 1000×

10 ml of 100% ethanol (Fisher, cat. no. 04‐355‐223)
0.125 g tetracycline hydrochloride (12.5 mg/ml final; Corning, cat. no. 61242RG)
Mix until dissolved
Aliquot and store protected from light up to 1 year at –20°C

### Transfer buffer, 1×

100 ml of 10× transfer buffer (see recipe)
700 ml dH_2_O
200 ml methanol (Fisher, cat. no. A433P) (add methanol last to prevent buffer precipitation)
Prepare fresh
  Working concentrations of 1×: 192 mM glycine, 25 mM Tris, and 20% (v/v) methanol.

### Transfer buffer, 10×

800 ml dH_2_O
144 g glycine (1.92 M final; Fisher, cat. no. BP3815)
30.2 g Tris base (250 mM final; Fisher, cat. no. BP152‐1)
Stir until fully dissolved
Adjust volume to 1 L with deionized water
Store up to 1 year at 4°C

### Tris, 1 M solution

75 ml dH_2_O
12.11 g Tris base (Fisher, cat. no. BP152‐1)
Adjust pH according to experimental requirements with 1 M HCl
Adjust volume to 100 ml with deionized water
Store up to 1 year at room temperature

### Tris, 1.5 M solution

70 ml dH_2_O
18.2 g Tris base (Fisher, cat. no. BP152‐1)
Adjust pH according to experimental requirements with 1 M HCl
Adjust volume to 100 ml with deionized water
Store up to 1 year at room temperature

### VHH‐HRP secondary antibody cocktail

Rabbit anti‐camelid VHH HRP‐conjugated antibody (GenScript, cat. no. A01861200)
Goat anti‐alpaca IgG VHH domain HRP‐conjugated antibody (Jackson ImmunoResearch, cat. no. 128‐035‐232)
Goat anti‐llama IgG VHH domain HRP‐conjugated antibody (Fisher, cat. no. 50‐271‐524)
*For ELISA*:
9.8 ml of 1× PBS (see recipe)
1 µl of each HRP‐conjugated antibody (final dilution of each antibody should be 1:10,000)
200 µl of 5% (w/v) BSA solution (see recipe)
Prepare fresh; do not store
*For western blot*:
10 ml of 5% milk/TBST (see recipe), added to a 15‐ml conical tube
Spike in 2 µl of each HRP‐conjugated antibody (final dilution of each antibody should be 1:5000)
Rock at room temperature for a few minutes prior to use
Prepare fresh; do not store

## COMMENTARY

### Critical Parameters

#### Animal work

Secure institutional approval for all proposed animal work and obtain appropriate training prior to project initiation.

#### Antigen selection and validation

Antigen selection and quality are the primary determinants of success in generating an antigen‐specific nanobody. First, consider antigen immunogenicity. Key factors include foreignness to the mouse (mouse or highly conserved mammalian proteins are often weakly immunogenic) and molecular size/complexity (small proteins/peptides typically require conjugation to a carrier, e.g., KLH and use of adjuvant). Antigen aggregation, post‐translational modifications, and the potential presence of endotoxins in antigen preparations originating from *E. coli* over‐expression cultures may increase apparent immunogenicity but can drive undesirable off‐target responses. Immunogenicity prediction tools can inform antigen design.

Next, obtain and validate the antigen, most commonly a purified protein. It is imperative to rigorously assess the quality of each antigen prior to immunization; do not rely on quality assurance provided by a vendor or collaborator. Upon receipt, aliquot and assess antigen within a few days by SDS‐PAGE/Coomassie staining to check for degradation, aggregation, or truncation. Consider additional, antigen‐specific validation (e.g., activity assays) to confirm functionality and avoid wasting animals and downstream effort.

#### Nucleic acid preparation, amplification, digestion, ligation

Basic Protocols 3 to 5 are derived from well‐established, relatively standard, and broadly used molecular biology procedures. Most are directly implemented from routine kits with step modifications to align them with the pipeline presented in this article. No major considerations to address.

#### Transformation via electroporation

If unfamiliar with the process, we recommend conducting a test transformation with a phagemid positive control and an aliquot of TG1 *E. coli* prior to library preparation (Basic Protocol 6) to optimize conditions for transformation and ensure functionality of the electroporator being used.

#### Working with phages

VCSM‐derived bacteriophages do not pose a bodily risk to the researcher, but they will readily infect and contaminate *E. coli* samples. Unwanted phage contamination can confound experiments and ruin stocks. Conclude all phage work before beginning any other bacterial work and disinfect all materials thoroughly with 10% bleach before autoclaving or washing. Do not share incubators when working with phages. Always use filtered pipette tips when working with phages. Use Parafilm to wrap lids when centrifuging. The phages used in this protocol require an F+ bacterial host; using phage‐resistant F– bacteria in other experiments may mitigate the risk and impact of uncontrolled infection.

#### Single colony ELISA

Basic Protocol 8 has straightforward steps but can yield unpredictable results for a variety of reasons and might require antigen‐specific optimizations. For example, the timing of the IPTG induction might need to be adjusted. Setting up several identical deep well plates from the starter culture and inducing them at different time points will elucidate the best expression conditions for each nanobody candidate. Coating the ELISA plates using purified antigen at 1 µg/ml is recommended, but some antigens may require higher concentrations. Once a reliable positive control has been identified, use it consistently to verify reagents are working properly.

#### Sequence enrichment and cloning

The most critical parameter that should be accounted for when selecting candidate nanobody sequences for testing and expression is the novelty of the sequence(s). Beware of promiscuous binders; if a candidate nanobody shows enrichment in multiple pannings against distinct antigens, exclude it from further considerations. Similarly, if a purified nanobody binds to multiple proteins in validation tests, de‐prioritize it. Keep detailed records of all candidate nanobody sequences across all nanobody development campaigns and query new nanobody sequences against previously identified nanobody sequences to confirm uniqueness and specificity of each nanobody. During geneblock design, the codon usage of the VHH gene can be optimized to match the expression host (bacteria or yeast), or it can be used to generate a humanized version of the nanobody.

#### Nanobody expression and purification

The nanobody expression and purification protocol will require parameter optimization on a nanobody‐by‐nanobody basis. Some nanobodies purify in mg quantities per liter of *E. coli* culture following the protocol outlined in this article If nanobody yield is low (<1 mg/L *E. coli* culture), consider adjusting IPTG concentration, induction temperature, and length of induction. However, in some cases, nanobody yields will remain low despite using optimized conditions. Keep detailed accounts of the steps taken each time a nanobody is induced and purified for repeatability in the future.

### Troubleshooting

Potential problems associated with the protocols and their solutions are described in Table 8.

**Table 8:** Troubleshooting Guide for High‐Throughput Isolation of Nanomouse‐Derived VHH Domains

Basic Protocol section	Problem	Possible cause	Solution
Basic Protocol 1: Breeding (colony generation)	Low pup yield/poor breeding performance	Close relatedness (littermates/closely related breeders); homozygous dam; homozygous × homozygous pairing	Prefer heterozygous pairs or heterozygous (female) × homozygous; avoid littermate/closely related pairings; avoid homozygous dams and homozygous × homozygous when possible
Basic Protocol 1: Genotyping (agarose gel)	Poor band separation for close‐sized products	Gel conditions not optimized; insufficient run time	Prefer smaller gels (e.g., 50 ml) for cleaner images; extend run beyond 60 min and check every 5 min until separation is achieved; use TBE buffer if TAE is not sufficient for separation of small products, alternatively, 5% acrylamide gels run in TBE in a vertical apparatus can be used to separate PCR fragments around 300 bp at very high resolution
Basic Protocol 1: Genotyping (PCR/gel)	Heterozygous doublet not observed	Primer degradation/instability; PCR setup issue	If wild‐typle and homozygous controls are correct, make fresh primer dilutions and repeat PCR; order new primers if needed
	High background/smearing	Dirty tail lysate; too much template DNA	Clean up tail lysate DNA; titrate DNA input; dilute crude lysate before PCR
Basic Protocol 2: Target/antigen	No/poor nanobody generation	Antigen predicted to be weakly immunogenic (highly conserved/mouse protein; small/simple antigen)	Reassess immunogenicity (e.g., IEDB Analysis Resource); consider carrier conjugation (e.g., KLH) for small antigens and use of adjuvant
	Off‐target or misleading immune responses	Antigen aggregation, glycosylation/other PTMs, or endotoxin increases apparent immunogenicity	Validate antigen quality; minimize aggregation/endotoxin where possible; interpret responses cautiously if these factors are present
Basic Protocol 2: Protein acquisition/QC	Antigen arrives degraded/aggregated/truncated/inactive	Vendor QC insufficient; shipping/handling; lot variability	Aliquot on receipt and assess within days by SDS‐PAGE/Coomassie; when feasible, produce in‐house or via an institutional core; add antigen‐specific activity assays as appropriate
Basic Protocol 2: Protein quantification	Measured concentration seems high, but gel looks weak/dirty	Co‐purifying host proteins inflate spectrophotometric/assay‐based concentration	Use Coomassie staining (with BSA standards) to assess purity/relative amount; treat “high concentration” with caution if purity is low
Basic Protocols 2: Protein compliance	Protein cannot be used in animals	Protein not approved for animal use under procurement rules	Confirm animal‐use approval and procurement compliance for each protein source before use
Basic Protocol 2: Terminal blood collection	Low blood yield during cardiac puncture	Vein nicked during chest opening, pooling/clotting	Use careful dissection to avoid nicking vessels; proceed as low as ∼100 µl if needed (downstream processing can still succeed)
Basic Protocol 2: Optional sera western	Multiple bands/difficult interpretation	Assay is suggestive; non‐reducing conditions; HcAb dimer/HC species expected	Interpret cautiously; expect strongest signal at 90‐100 kDa and additional 35‐45 kDa bands under non‐reducing conditions; an IP with Protein A beads may provide a cleaner signal but has not been tested in this serum blot assay
Basic Protocol 3: RNA	Low RNA yield	RNA degradation during purification process	Ensure that RNA is always kept on ice and all surfaces have been treated with RNase‐Zap
	High DNA contamination, no RNA	Wrong layer was taken during acid‐phenol‐chloroform extraction	DNA goes in the organic phase when phenol is acidic; this is why TE‐phenol (pH 8.0) is used to extract DNA from samples because phenol at pH 8.0 allows the DNA to remain in the aqueous phase
Basic Protocol 4: cDNA	Low cDNA yield	mRNA degradation prior to reverse transcription; incorrect thermocycler program; reverse transcriptase has degraded	If issue persists across RNA samples, try replacing the qScript; if still unresolved, increase application of RNase‐Zap; check that the correct program was entered on thermocycler
Basic Protocol 4: PCR/gel	Smears instead of discrete, albeit fuzzy bands in VHH gel	Primers are degraded	If problem occurs across a full set of reverse primers or consistently with the same forward primer, replace that primer
Basic Protocol 6: Electroporation	No colonies after electroporation	Electroporator not functioning/poor settings; degraded competent TG1; equipment rarely used/shared resource	Perform test transformations using a phagemid‐plasmid positive control and TG1 aliquot to validate conditions; service/replace electroporator if tests repeatedly fail
Basic Protocol 6: Electroporation	Arc during electroporation	Moisture on the cuvette short circuiting the electrical impulse, too many salts in the DNA buffer increasing conductivity	Wipe off any moisture with a Kimwipe, if error still occurs, dilute and clean DNA in water
Basic Protocol 6: TG1 library	Uncountable lawns; cannot estimate library complexity	Antibiotic selection failure	Proceed to Basic Protocol 7; reapply antibiotic selection prior to panning
	Very low CFU	Low transformation efficiency and/or suboptimal electroporation conditions	Validate electroporation with positive‐control transformation; optimize conditions as needed
Basic Protocol 7: Phage pelleting	Unfamiliar pellet appearance	Normal “feathery” phage pellet that can streak up tube wall	Treat as expected pellet morphology; proceed with care to avoid disturbing pellet
Basic Protocol 7: Post‐panning	Few colonies post‐panning	Low recovery; stock quality issues (ER2738 and/or phage stock)	If rare low‐yield plates occur, pick all colonies for ELISA; if repeated, re‐propagate competent ER2738 stock, phage stock, or both
	No growth on any plates	Incorrect antibiotics, incorrect cell type, cells were killed, glycine was not neutralized before adding to cells	Verify antibiotics are correct, verify cell type is correct, follow steps carefully when acid‐eluting phages
	Lawns on all growth plates	Antibiotic failure	Verify activity of relevant antibiotics, make new plates
Basic Protocol 8: ELISA screening	No hits from ELISA Plates	Weakly immunogenic antigen; assay/detection failure	Include a positive control (purified nanobody or nanobody‐expressing bacterium) to verify colorimetric readout; consider alternative screening methods if ELISA is unsuitable
Basic Protocol 8: ELISA readout	Entire plate shows identical values	Technical failure (assay setup) or plate reader malfunction	Troubleshoot assay setup and verify plate reader function; confirm controls behave as expected
Basic Protocol 8: ELISA throughput	No hits despite working controls	True negative campaign or insufficient screening depth	Cap single‐colony ELISA at 10 × 96‐well plates; sequence TG1 and panning libraries to look for enriched sequences
Basic Protocol 9: Post‐ELISA candidate selection	Strong/enriched hits later prove off target (“sticky”)	Promiscuous binders recurring across antigens	Verify novelty by querying prior sequence records; test candidates against non‐target proteins; maintain a searchable sequence database for future campaigns
Basic protocol 9: Sequence alignment and selection for cloning	Truncated nanobody amino acid sequence.	Premature stop codons	Confirm by looking at the chromatogram that the sequencing result is clean and real; if it is real, it will likely not be a good candidate for expression/purification/cloning; if the result is due to a messy chromatogram, send for sequencing again and reevaluate
Basic Protocol 10: Geneblock design	Insert translates out‐of‐frame	Incorrect overlap/orientation or reading frame	Verify in‐frame translation in silico (e.g., ApE; see Internet Resources) before cloning
	Gene fragments are too short for vendor to create	Nanobody is a shorter sequence than prototypical nanobodies	Extend the overhangs evenly on each size of the fragment until the minimum length has been met
	Gene fragments fail vendor complexity tests	Gene fragment contains repetitive structures, strong homology, high/low GC content	Start with a simple codon optimization of the insert (do not optimize the overhangs), if this does not resolve the issue, consult with the vendor; if it is too complex for the vendor to synthesize, then it is likely not a good candidate to move forward with
Basic protocol 10: Transformation of cloning reaction into DH5α *E. coli*	No transformant colonies on the plates after overnight incubation	Slow growth at 37°C	Leave the plates out at room temperature for 48‐72 hr and check again, if there are still no colonies, use a PCR cleanup kit on the cloning reaction and try the transformation again; if the second attempt at transformation does not work, try changing the molar ratios of the geneblocks vs cloning vector (see the NEB website for the Gibson Assembly protocol for suggestions)
Basic protocol 10: Gibson Assembly	High rate of sequence errors (e.g., mutations, insertions, deletions, and/or frameshifts)	Cloning/assembly artifacts; colony‐to‐colony variation	Confirm by whole‐plasmid sequencing; sequence multiple colonies per construct (e.g., 3) to increase chance of a perfect clone; repeat/adjust assembly if persistent
	Many colonies screened without a perfect clone	Suboptimal assembly inputs/conditions; reaction impurities	Repeat assembly and/or adjust input ratios; clean and concentrate the assembly reaction prior to transformation
Basic Protocol 11: Expression and purification	Little/no expression in purification lane	Induction did not occur; incorrect construct/sequence; suboptimal induction conditions	Compare uninduced vs induced; look for ∼15 kDa band; verify construct/sequence; optimize OD at induction, IPTG concentration, temperature, and induction time
	Expression present but mainly insoluble	Lysis inefficiency or periplasmic secretion issues	Increase TES volume during initial resuspension; reassess lysis/secretion conditions
	Aggregation/degradation during expression	Excessive induction causing secretion overload, precursor accumulation, mispaired disulfides	Reduce expression intensity (lower IPTG, lower temperature, shorter induction); sonicate insoluble fraction if needed (may reduce purity)
	Low yield despite apparent correct expression	Culture volume too small for low‐expressing clone	Scale up culture volume to increase recovered nanobody
Basic Protocol 11: Expression and purification/ dialysis	Quantification inconsistent with gel or unexpectedly low after dialysis	Co‐purified contaminants inflate spectrophotometric/assay‐based concentration; protein loss during dialysis (e.g., pinhole/tear)	Do not rely on quantification alone; confirm by SDS‐PAGE/Coomassie (pre‐ or post‐dialysis); quantify after dialysis; inspect/verify dialysis tubing integrity
Basic Protocol 11: Expression and purification/post‐dialysis	Precipitation after dialysis	Buffer not compatible (pH/salt); imidazole‐associated aggregation, protein too concentrated	Optimize buffer (pH/salt); dilute eluate immediately into a larger volume of imidazole‐free buffer to reduce aggregation; increase volume of elution buffer during purification; begin dialysis at room temperature before moving to 4°C

### Understanding Results

In our nanomouse colony, heterozygous breeding pairs produce 8 to 10 pups per litter at Mendelian ratios for up to 7 months. Heterozygous × homozygous pairs produce 6 to 8 pups per litter at Mendelian ratios for up to 6 months when the heterozygous animal is the female, improving the odds of obtaining experimental pups. This strategy is less successful when breeders are littermates or closely related. We have had poor results with homozygous × homozygous pairs or when the dam is homozygous; these pairings typically yield infrequent, small litters (often 1 to 3 pups) with low pup survival.

Figure 1 shows a representative agarose gel image. Always include a heterozygous control to confirm both bands are detectable. Run the gel until the two bands are separated. If the heterozygous doublet is absent but wild‐type and homozygous controls look correct, make fresh primer dilutions, and repeat PCR; if needed, order new primers. If background is high, clean up the tail lysate and titrate DNA input, or dilute the crude lysate before PCR. The gel run in this figure was prepared and run in 1× TAE; if resolution is challenging, TBE is a preferable option for resolving smaller bands.

Figure 2 shows a representative Coomassie‐stained polyacrylamide gel loaded with a relatively clean, non‐degraded, and non‐aggregated protein. The BSA dilutions are useful for comparison of expected intensity of the stain based on µg of protein loaded. Proteins that are not very pure or copurify with host proteins, e.g., will quantitate at a higher concentration than they may appear on the Coomassie stain.

Female mice are preferred for immunization campaigns because they fit well in the restrainer for tail bleeding and can be co‐housed as non‐littermates, reducing housing costs. We have not observed any sex‐based differences in immune response. Tail‐bleed serum yield is variable (10 to 75 µl), since optional serum westerns require ≤3 µl, collection is not optimized, and restrainer time is limited to ≤7 min to minimize distress. For terminal collection, the goal is ∼600 µl whole blood (100 µl for serum, 500 µl for RNA extraction), as larger volumes have not improved RNA yield; even ∼100 µl can be sufficient. Cardiac puncture complications most often result from inadvertently nicking a vein during chest opening, leading to pooling/clotting. Spleen and bone marrow RNA yields typically exceed protocol needs; fat/adherent tissue can be trimmed liberally, and bone marrow is collected from femurs only.

The optional sera western often produces complex banding and should be interpreted cautiously, as it is suggestive rather than conclusive. Probing membranes containing serum from immunized nanomice with anti‐camelid HRP secondary antibodies frequently reveals multiple bands, whereas wild‐type mouse serum shows no signal (Fig. 3B). This indicates an ongoing immune response in the nanomouse to eliminate the used antigen. Nanomouse design impairs light‐chain binding via CH1 deletion (Xu et al., 2021), yielding secreted heavy chain–only antibodies (HcAbs) that dimerize via the hinge: the expected dimer is 90 to 100 kDa, and the CH1‐deleted heavy chain should run 35 to 45 kDa (vs 45 to 50 kDa for conventional murine IgG heavy chain) (Muyldermans, 2013). Because samples are not fully reducing (non‐reducing Laemmli; no β‐ME/DTT), signal is typically strongest at 90 to 100 kDa, with additional 35 to 45 kDa bands likely arising from partial reduction during brief boiling. Signal intensity does not predict future success in nanobody discovery. Instead, combining phage libraries from animals immunized with the same antigens has proven effective. High‐intensity sera can serve as a useful pilot reagent for FACS, immunofluorescence, or western proof‐of‐concept to assess antigen reactivity, but it does not identify sequence.

The RNA extraction methods use established kits and typically yield total RNA of 15 to 50 µg from blood, 300 to 750 µg from pooled spleen, and 75 to 150 µg from pooled bone marrow. cDNA synthesis is straightforward, and the full cDNA is used for VHH‐specific PCR. VHH PCR products often look inconsistent, i.e., smears, nonspecific bands, or no visible bands (Fig. 5), which usually reflects immune repertoire variability rather than protocol failure, assuming reagents are intact and steps were followed. Occasionally, amplicons associated with a given reverse primer are weak or absent, likely because that motif is rare in the repertoire (e.g., the nanomouse signature TTVTSS); that primer should be refreshed regularly to rule out primer degradation. Band patterns and intensities represent the overall immune population and do not predict on target nanobody quality, making downstream screening (Basic Protocol 7) essential to identify enriched binders.

By the end of Basic Protocol 6, five TG1 libraries should be generated: one from each of the three blood samples, one from the pooled spleen sample, and one from the pooled bone marrow sample. Library yields are highly variable across tissue types (observed range: 3.5 × 10^3^ to 4.3 × 10^6^ CFU). CFU alone is not a reliable success metric; however, zero‐colony outcomes have not been observed. Occasional antibiotic failure can produce uncountable lawns that prevent complexity estimation; in these cases, Basic Protocol 7 can still proceed, as selection can be reapplied before panning. TG1 CFU counts are only weakly predictive of phage panning enrichment: very low CFU often leads to low panning yields, but high CFU does not guarantee strong enrichment. More rigorous OD–CFU analysis may be informative but has not been considered an efficient use of time given library‐to‐library variability.

Basic Protocol 7 is technically straightforward but requires strict phage‐handling practices to prevent laboratory contamination, which can disrupt cell cultures and is difficult to remediate. During phage pelleting, the pellet may appear “feathery” and streak up the tube wall. Post‐panning CFU expectations are variable: completely blank plates have not been observed, but rare low‐yield plates with only a few colonies should be exhaustively picked for ELISA screening, whereas robust growth typically yields discrete colonies only on dilution plates. Recurrent low colony recovery may warrant re‐propagation of competent ER2738 stock, phage stock, or both. A traditional overnight ampicillin/kanamycin CFU infection assay is described in the main text; however, an alternate rapid method can be used by lysing an aliquot of the virion suspension in 7 M urea/2 M NaCl to release single‐stranded DNA followed by an immediate OD260 measurement. A laboratory‐specific correlation curve between OD260 and functional CFU should be established to account for non‐infective particles.

Failure to obtain ELISA‐positive hits is not uncommon, and some campaigns do not yield binders, especially with weakly immunogenic antigens. Including a positive control (purified nanobody or nanobody‐expressing bacterium) helps distinguish true negatives from general assay failure. Completely signal uniformity across all wells of a given 96‐well plate may indicate a technical or plate reader issue, as some well‐to‐well variation is expected even on negative plates. If controls perform as expected but no hits are detected, consider alternative methods to detect enriched nanobody sequences, e.g., NGS. Failure to retrieve binders may also be attributed to potential antigen fold deformation or degradation during adsorption to a binding plate. To mitigate this, consider using a biotinylated antigen and streptavidin beads or streptavidin‐coated plate to maintain the proper folding of the antigen in phage biopanning.

After hit wells are identified in the ELISA, the corresponding sequences are determined, and candidates are prioritized for cloning, expression, and purification. Redundant/enriched sequences are generally favored, as repeated recovery indicates positive selection for a given nanobody sequence. When distinct nucleotide variants encode the same amino acid sequence, selection is based on abundance and/or ELISA binding strength. Strong, non‐redundant binders are also advanced. If many hits are weak, a representative set is purified and tested individually and in combination, since nanobodies can show additive or synergistic effects (Mast et al., 2021). Before cloning, sequences should be cross‐checked against prior pipeline outputs to exclude recurring “sticky” or promiscuous off‐target binders.

Confirm successful cloning of nanobody sequences into expression plasmids by whole‐plasmid sequencing. Sequencing 3 colonies per construct is usually sufficient to identify at least one correct plasmid. Rarely, >10 colonies must be screened to identify a perfect sequence.

Nanobody expression and purification protocols may require significant troubleshooting depending on the properties of your nanobody. Often, many candidates are identified, but only a subset express and purify well, and failures may reflect multiple variables. SDS‐PAGE/Coomassie is essential for diagnosing issues (Fig. 11 shows a typical successful result). If the purification lane is weak or blank, first confirm induction by comparing uninduced vs induced samples and looking for an enriched ∼15 kDa band; if absent, review construct/sequence and induction parameters (OD at induction, IPTG concentration, temperature, and induction time). If a ∼15 kDa band appears but is enriched in the insoluble fraction, this suggests lysis or periplasmic secretion problems; increasing TES volume during initial resuspension is often an effective first adjustment. Excessive induction can impede secretion, promote degradation, or cause mis‐paired disulfides and aggregation; mitigation typically involves reducing expression (lower IPTG, lower temperature, shorter induction), though sonication of insoluble material is possible at the cost of purity. If low expression persists without an identifiable technical issue, scaling up culture volume can increase yield.

Coomassie confirmation should not be replaced by protein quantification alone; quantify protein after dialysis, as co‐purified contaminants can inflate OD280 or other spectrophotometric assay‐based concentration measurements and losses can occur during dialysis (e.g., tubing defects). Running SDS‐PAGE/Coomassie after dialysis is also acceptable.

### Time Considerations

A summary of the duration of time required for each Basic Protocol can be found in Table 9.

**Table 9:** Comprehensive Timeline: Nanobody Production Pipeline

Protocol	Time requirements	Cumulative timeline
Basic Protocol 1: Nanomouse breeding, genotyping, and colony establishment	1‐2 weeks for mouse arrival/quarantine; 30 min/week for maintenance; 24‐48 hr for DNA extraction/genotyping; 19‐21 days for gestation; 14‐21 days for tail sampling from pups; 8‐12 weeks to breed; repeat for at least 1 generation	30‐42 weeks to prior to starting immunizations
Basic Protocol 2: Nanomouse immunization and tissue collection	9 weeks total duration (∼1 hr per round, typically 4‐5 rounds); 1 day for tissue collection and flash‐freezing	Weeks 1‐9
Basic Protocol 3: RNA extraction from nanomouse immune cells isolated from blood and tissues	1‐2 days for RNA extraction	Week 9 (+3 days)
Basic Protocol 4: Generation and amplification of VHH DNA from nanomouse cDNA	1 day for cDNA amplification; 1‐3 days for VHH specific PCR, visualization, and purification of PCR reaction	Week 10
Basic Protocol 5: Digestion of VHH DNA and ligation into a phagemid expression vector	1 day for digestion, 1‐3 days for gel purifications, 1 day for ligation and cleanup	Weeks 10‐11
Basic Protocol 6: Preparation of a screenable *E. coli* TG1‐based phagemid library	1‐2 weeks for 3‐5 tissue‐specific libraries targeting the same antigen	Weeks 12‐14
Basic Protocol 7: Antigen‐driven VHH selection using phage display	2‐3 weeks for multiple rounds of enrichment	Weeks 13‐17
Basic Protocol 8: Single colony VHH ELISA screening	1 week for ELISA testing	Weeks 14‐18
Basic Protocol 9: Sequencing of ELISA hits and candidate VHH sequence identification	1 week for culturing, sequencing, and evaluating VHH sequence to determine candidates to clone	Weeks 15‐19
Basic Protocol 10: Geneblock design of candidate VHH and Gibson Assembly into a nanobody expression vector	1 day for geneblock design; 1‐3 days for geneblocks to arrive; 1 day for cloning; 3‐5 days for transformation, culturing, and whole plasmid sequencing	Weeks 16‐20
Basic Protocol 11: Nanobody over‐expression and purification	1‐2 weeks for protein expression and final purification	Weeks 17‐21

The initial phase of the pipeline is dominated by animal preparation and the biological response time of the nanomouse. Following a 1‐ to 2‐week quarantine and setup period, it will take 30 to 42 weeks to establish a breeding colony (Basic Protocol 1) and 9 weeks for the immunization protocol (Basic Protocol 2) representing the most significant time investments. Because booster injections must be spaced exactly two weeks apart to allow for proper immune maturation, this stage serves as a fixed bottleneck in the schedule that cannot be accelerated.

Once immunization is complete, the workflow transitions to bench work. The process of tissue collection takes 1 day and the downstream molecular biology steps (Basic Protocols 3 to 5: RNA extraction, cDNA processing, VHH amplification) can be completed in ∼1 week. The TG1 library preparations for all tissues in an antigen‐targeting cohort of mice will take ∼2 weeks. The selection process via biopanning (Basic Protocol 7) typically requires 2 to 3 weeks of iterative enrichment, while subsequent ELISA screening and sequencing (Basic Protocol 8) take an additional week to identify successful candidates.

Identifying candidate sequences and cloning the into a pHEN vector via Gibson Assembly (Basic Protocols 9 and 1^0^) to the final stage of protein expression and purification (Basic Protocol 11) generally concludes within 1 to 4 weeks. In total, the cycle from initial mouse acquisition to a purified nanobody product spans 21 to 50 weeks (5 to 11 months). Researchers can optimize this timeline by preparing molecular biology reagents and performing vector validation in parallel during the lengthy immunization period.

### Author Contributions

**Tessa Casselman**: Conceptualization; methodology; validation; visualization; writing—original draft; writing—review and editing. **Asa Huffaker**: Conceptualization; data curation; methodology; validation; visualization; writing—original draft; writing—review and editing. **Kristie Mitchell**: Conceptualization; data curation; methodology; validation; visualization; writing—original draft; writing—review and editing. **Jamie Schnarrs**: Methodology; writing—review and editing. **Sylvia Ni**: Methodology; writing—review and editing. **Mary Skinner**: Conceptualization; data curation; methodology; project administration; supervision; validation; visualization; writing—original draft; writing—review and editing. **Matthias Truttmann**: Conceptualization; funding acquisition; methodology; project administration; supervision; writing—original draft; writing—review and editing.

### Conflict of Interest

Authors declare no competing interest.

## Data Availability

Data sharing is not applicable to this article as no new data were created or analyzed in this study.

## References

[cpz170432-bib-0001] Arbabi Ghahroudi, M. , Desmyter, A. , Wyns, L. , Hamers, R. , & Muyldermans, S. (1997). Selection and identification of single domain antibody fragments from camel heavy‐chain antibodies. FEBS Letters, 414(3), 521–526. 10.1016/s0014-5793(97)01062-4 9323027

[cpz170432-bib-0002] Barderas, R. , Shochat, S. , Martínez‐Torrecuadrada, J. , Altschuh, D. , Meloen, R. , & Ignacio Casal, J. (2006). A fast mutagenesis procedure to recover soluble and functional scFvs containing amber stop codons from synthetic and semisynthetic antibody libraries. Journal of Immunological Methods, 312(1–2), 182–189. 10.1016/j.jim.2006.03.005 16674972

[cpz170432-bib-0003] Conrath, K. E. , Lauwereys, M. , Galleni, M. , Matagne, A. , Frère, J.‐M. , Kinne, J. , Wyns, L. , & Muyldermans, S. (2001). Β‐lactamase inhibitors derived from single‐domain antibody fragments elicited in the *camelidae* . Antimicrobial Agents and Chemotherapy, 45(10), 2807–2812. 10.1128/aac.45.10.2807-2812.2001 11557473 PMC90735

[cpz170432-bib-0004] Current Protocols . (2006). Commonly Used Reagents. Current Protocols in Microbiology, 00, A.2A.1–A.2A.15. 10.1002/9780471729259.mca02as00

[cpz170432-bib-0005] Devasani, J. R. , Guntuku, G. , Sarabu, P. , Muthyala, M. K. , Palla, M. S. , & Subrahmanyam Volety, M. (2025). Integrative and emerging models in Antibody Research: A comprehensive review. Antibody Therapeutics, 8(4), 317–335. 10.1093/abt/tbaf018 41367414 PMC12683041

[cpz170432-bib-0006] Glockshuber, R. , Schmidt, T. , & Plueckthun, A. (1992). The disulfide bonds in antibody variable domains: Effects on stability, folding in vitro, and functional expression in escherichia coli. Biochemistry, 31(5), 1270–1279. 10.1021/bi00120a002 1736986

[cpz170432-bib-0007] Hamers‐Casterman, C. , Atarhouch, T. , Muyldermans, S. , Robinson, G. , Hammers, C. , Songa, E. B. , Bendahman, N. , & Hammers, R. (1993). Naturally occurring antibodies devoid of light chains. Nature, 363(6428), 446–448. 10.1038/363446a0 8502296

[cpz170432-bib-0008] Hoogenboom, H. R. , Griffiths, A. D. , Johnson, K. S. , Chiswell, D. J. , Hudson, P. , & Winter, G. (1991). Multi‐subunit proteins on the surface of filamentous phage: Methodologies for displaying antibody (FAB) heavy and light chains. Nucleic Acids Research, 19(15), 4133–4137. 10.1093/nar/19.15.4133 1908075 PMC328552

[cpz170432-bib-0009] Hoogenboom, H. R. , de Bruïne, A. P. , Hufton, S. E. , Hoet, R. M. , Arends, J.‐W. , & Roovers, R. C. (1998). Antibody phage display technology and its applications. Immunotechnology, 4(1), 1–20. 10.1016/s1380-2933(98)00007-4 9661810

[cpz170432-bib-0010] Karyolaimos, A. , & de Gier, J.‐W. (2021). Strategies to enhance periplasmic recombinant protein production yields in escherichia coli. Frontiers in Bioengineering and Biotechnology, 9, 797334. 10.3389/fbioe.2021.797334 34970535 PMC8712718

[cpz170432-bib-0011] Ledsgaard, L. , Kilstrup, M. , Karatt‐Vellatt, A. , McCafferty, J. , & Laustsen, A. H. (2018). Basics of Antibody Phage Display Technology. Toxins, 10(6), 236. 10.3390/toxins10060236 29890762 PMC6024766

[cpz170432-bib-0012] Mast, F. D. , Fridy, P. C. , Ketaren, N. E. , Wang, J. , Jacobs, E. Y. , Olivier, J. P. , Sanyal, T. , Molloy, K. R. , Schmidt, F. , Rutkowska, M. , Weisblum, Y. , Rich, L. M. , Vanderwall, E. R. , Dambrauskas, N. , Vigdorovich, V. , Keegan, S. , Jiler, J. B. , Stein, M. E. , Olinares, P. D. B. , … Rout, M. P. (2021). Highly synergistic combinations of nanobodies that target SARS‐COV‐2 and are resistant to escape. eLife, 10, 73027. 10.7554/elife.73027 PMC8651292 34874007

[cpz170432-bib-0013] McMahon, C. , Baier, A. S. , Pascolutti, R. , Wegrecki, M. , Zheng, S. , Ong, J. X. , Erlandson, S. C. , Hilger, D. , Rasmussen, S. G. , Ring, A. M. , Manglik, A. , & Kruse, A. C. (2018). Yeast surface display platform for rapid discovery of conformationally selective nanobodies. Nature Structural & Molecular Biology, 25(3), 289–296. 10.1038/s41594-018-0028-6 PMC5839991 29434346

[cpz170432-bib-0014] Modafferi, D. , de Medeiros Dantas, J. M. , & Dorval Courchesne, N.‐M. (2025). Maximizing yield, purity and throughput of M13 bacteriophage bioprocessing. Biochemical Engineering Journal, 224, 109890. 10.1016/j.bej.2025.109890

[cpz170432-bib-0015] Moutel, S. , Bery, N. , Bernard, V. , Keller, L. , Lemesre, E. , de Marco, A. , Ligat, L. , Rain, J.‐C. , Favre, G. , Olichon, A. , & Perez, F. (2016). Nali‐H1: A Universal Synthetic Library of humanized nanobodies providing highly functional antibodies and intrabodies. eLife, 5, 16228. 10.7554/elife.16228 PMC4985285 27434673

[cpz170432-bib-0016] Muyldermans, S. (2013). Nanobodies: Natural single‐domain antibodies. Annual Review of Biochemistry, 82(1), 775–797. 10.1146/annurev-biochem-063011-092449 23495938

[cpz170432-bib-0017] Muyldermans, S. (2020). A guide to: Generation and design of nanobodies. The FEBS Journal, 288(7), 2084–2102. 10.1111/febs.15515 32780549 PMC8048825

[cpz170432-bib-0018] Muyldermans, S. (2021). Applications of nanobodies. Annual Review of Animal Biosciences, 9(1), 401–421. 10.1146/annurev-animal-021419-083831 33233943

[cpz170432-bib-0019] Schmidt, F. I. , Lu, A. , Chen, J. W. , Ruan, J. , Tang, C. , Wu, H. , & Ploegh, H. L. (2016). A single domain antibody fragment that recognizes the adaptor ASC defines the role of ASC domains in inflammasome assembly. Journal of Experimental Medicine, 213(5), 771–790. 10.1084/jem.20151790 27069117 PMC4854733

[cpz170432-bib-0020] Sosa, B. A. , Demircioglu, F. E. , Chen, J. Z. , Ingram, J. , Ploegh, H. L. , & Schwartz, T. U. (2014). How lamina‐associated polypeptide 1 (LAP1) activates Torsin. eLife, 3, 3239. 10.7554/elife.03239 PMC4358337 25149450

[cpz170432-bib-0021] Truttmann, M. C. , Wu, Q. , Stiegeler, S. , Duarte, J. N. , Ingram, J. , & Ploegh, H. L. (2015). Hype‐specific nanobodies as tools to modulate hype‐mediated target ampylation. Journal of Biological Chemistry, 290(14), 9087–9100. 10.1074/jbc.m114.634287 25678711 PMC4424278

[cpz170432-bib-0022] Vieira, J. , & Messing, J. (1987). [1]production of single‐stranded plasmid DNA. Methods in Enzymology, 3–11. 10.1016/0076-6879(87)53044-0 3323803

[cpz170432-bib-0023] Vincke, C. , Gutiérrez, C. , Wernery, U. , Devoogdt, N. , Hassanzadeh‐Ghassabeh, G. , & Muyldermans, S. (2012). Generation of single domain antibody fragments derived from camelids and generation of manifold constructs. Methods in Molecular Biology, 145–176. 10.1007/978-1-61779-974-7_8 22907350

[cpz170432-bib-0024] Xu, J. , Xu, K. , Jung, S. , Conte, A. , Lieberman, J. , Muecksch, F. , Lorenzi, J. C. C. , Park, S. , Schmidt, F. , Wang, Z. , Huang, Y. , Luo, Y. , Nair, M. S. , Wang, P. , Schulz, J. E. , Tessarollo, L. , Bylund, T. , Chuang, G.‐Y. U. , Olia, A. S. , … Casellas, R. (2021). Nanobodies from camelid mice and llamas neutralize SARS‐COV‐2 variants. Nature, 595(7866), 278–282. 10.1038/s41586-021-03676-z 34098567 PMC8260353

[cpz170432-bib-0025] Zhu, Y. , Padgett, L. , Dinh, H. , Marcovecchio, P. , Wu, R. , Hinz, D. , Kim, C. , & Hedrick, C. (2019). Preparation of whole bone marrow for mass cytometry analysis of neutrophil‐lineage cells. Journal of Visualized Experiment s, (148). 10.3791/59617 PMC6726111 31282876

[cpz170432-bib-0026] Zimmermann, I. , Egloff, P. , Hutter, C. A. , Arnold, F. M. , Stohler, P. , Bocquet, N. , Hug, M. N. , Huber, S. , Siegrist, M. , Hetemann, L. , Gera, J. , Gmür, S. , Spies, P. , Gygax, D. , Geertsma, E. R. , Dawson, R. J. , & Seeger, M. A. (2018). Synthetic single domain antibodies for the conformational trapping of membrane proteins. eLife, 7, 34317. 10.7554/elife.34317 PMC5967865 29792401

[cpz170432-bib-0028] https://www.bio‐rad.com/en‐us/applications‐technologies/transfer‐conditions?ID=LUSPTIMNI

[cpz170432-bib-0030] https://documents.thermofisher.com/TFS‐Assets%2FLSG%2Fmanuals%2Fpurelink_pcr_man.pdf

[cpz170432-bib-0032] https://www.neb.com/en‐us/protocols/m13‐titer‐protocol

[cpz170432-bib-0034] https://www.qiagen.com/us/resources/download.aspx?id=22df6325‐9579‐4aa0‐819c‐788f73d81a09&lang=en

[cpz170432-bib-0036] https://ugene.net/

[cpz170432-bib-0038] www.twistbioscience.com

[cpz170432-bib-0040] https://jorgensen.biology.utah.edu/wayned/ape/

[cpz170432-bib-0042] https://www.neb.com/en‐us/protocols/gibson‐assembly‐protocol‐e5510?srsltid=AfmBOoos0yLoTL3‐Cye4nWRFhslacbmQ_vzcdPXZu0d8‐nCGSong1ptP

[cpz170432-bib-0044] https://plasmidsaurus.com/

